# Unusual Secondary Metabolites of the Aerial Parts of *Dionysia diapensifolia* Bioss. (Primulaceae) and Their Anti-Inflammatory Activity

**DOI:** 10.3390/biom10030438

**Published:** 2020-03-12

**Authors:** Mostafa Alilou, Stefania Marzocco, Hossein Batooli, Jakob Troppmair, Stefan Schwaiger, Hermann Stuppner

**Affiliations:** 1 Institute of Pharmacy, Pharmacognosy, Center for Molecular Biosciences (CMBI), University of Innsbruck, 6020 Innsbruck, Austria; mostafa.alilou@student.uibk.ac.at (M.A.); hermann.stuppner@uibk.ac.at (H.S.); 2 Daniel-Swarovski Research Laboratory, Department of Visceral, Transplant and Thoracic Surgery, Innsbruck Medical University, Innrain 66, 6020 Innsbruck, Austria; jakob.troppmair@i-med.ac.at; 3 Department of Pharmacy, University of Salerno, Via Giovanni Paolo II 132, I-84084 Fisciano, SA, Italy; 4 Kashan Botanical Garden, Research Institute of Forests and Rangelands, Agricultural Research, Education and Extension Organization (AREEO), Tehran 193951113, Iran; ho_batooli@yahoo.com

**Keywords:** *Dionysia diapensifolia* Bioss, anti-inflammatory, macrophages, phenolic compounds, flavone

## Abstract

The genus *Dionysia*, belonging to the Primulaceae family, encompasses more than 50 species worldwide with a center of diversity located in the arid Irano-Turanian mountains. In this study, a phytochemical investigation of the aerial parts of *D. diapensifolia* Bioss. led to the isolation of 24 phenolic compounds **1**–**7** and **9**–**25**, and one sesquiterpenoid **8**. Compound **1** was identified as new natural product, while isolation of **2** and **3**, already known as synthetic products, from a natural source is reported for the first time in the present study. Isolation of compound **8** from a *Dionysia* species and indeed the whole Primulaceae family is reported for the first time too. Structure elucidation was performed by extensive spectroscopic analyses (1D-, 2D-NMR, and MS), and by comparison with reported literature data. Furthermore, DP4+ chemical shift probability calculations were performed to establish the relative configuration of compound **1**. Additionally, subfractions obtained by liquid-liquid extraction of the methanolic extract of the plant, and subsequently the isolated new and selected known compounds **1**–**4**, **6**, **8**–**11** obtained from the diethyl ether subfraction were investigated for their inhibitory effect on NO release and iNOS and COX-2 expression in J774A.1 murine macrophages. The results showed a potential anti-inflammatory activity of the obtained subfractions, of which the diethyl ether subfraction was the most active one in inhibiting NO release and COX-2 expression (*p* < 0.001). Among the investigated isolated compounds, compound **4** significantly (*p* < 0.001) inhibited NO release and iNOS and COX-2 expression in a comparable manner like the used positive controls (L-NAME and indomethacin, respectively). Moreover, other isolated substances displayed moderate to high inhibitory activities, illustrating the potential anti-inflammatory activity of *Dionysia diapensifolia*.

## 1. Introduction

Inflammation is a pathophysiological process, identified by redness, edema, pain and other symptoms, which plays a pivotal role in protecting the body from injury and infection [1]. However, prolonged inflammatory responses are the most important cause of chronic inflammatory diseases such as atherosclerosis, obesity and different types of cancer [1,2]. Macrophages play a substantial role in inflammatory responses by mediating the upregulation of inflammatory mediators like nitric oxide (NO), inducible nitric oxide synthase (iNOS), cyclooxygenase-2 (COX-2), interleukin-6 (IL-6), IL-12 and tumor necrosis factor α (TNF-α) [3]. Especially macrophage activation, mediated by stimuli such as LPS, lead to elevated levels of inflammatory mediators. Long term activation of them can result in tissue damage and chronic inflammation [4]. Thus, the reduction of these inflammatory mediators is a promising strategy for the discovery of nonsteroidal anti-inflammatory drugs (NSAIDs). Nowadays, due to the role of inflammation and associated side effects of existing anti-inflammatory drugs, particularly on the gastrointestinal tract, there is tremendous demand for anti-inflammatory agents with low risk profile. Plants provide a broad range of complex and diverse natural compounds with potential anti-inflammatory activities. Polyphenols are a class of natural products that are involved in the defense system, genome protection (genomic stability) and pigmentation of plants [5]. Flavonoids are the most known and most common plant secondary metabolites with a wide range of pharmacological activity such as antibacterial, anticancer, anti-inflammatory and immunomodulatory activity [5,6].

The genus *Dionysia* (Primulaceae) has its center of diversity in the arid Irano-Turanian mountains [7]. Worldwide it comprises more than 50 species [8], most of them of interest due to their colorful flowers, which bloom in spring and summer. Previous studies on the exudate flavonoids of *Dionysia* species indicated the presence of typical primula type flavonoids with regular and irregular substitution patterns [8]. Apart from these investigations, only a few reports are available on the phytochemistry and the pharmacological activity of *Dionysia* species. One reported property is the anti-tumor and apoptosis inducing activity of an extract of *D. termeana* [9]. However, the responsible active substances have not been identified. Another phytochemical investigation on exudates of *D. diapensifolia*, led to the isolation of an unusual chalcone type compound **4** with anti-fungal activity. However, authors did not quantify the observed inhibition [10].

**Figure 1:**
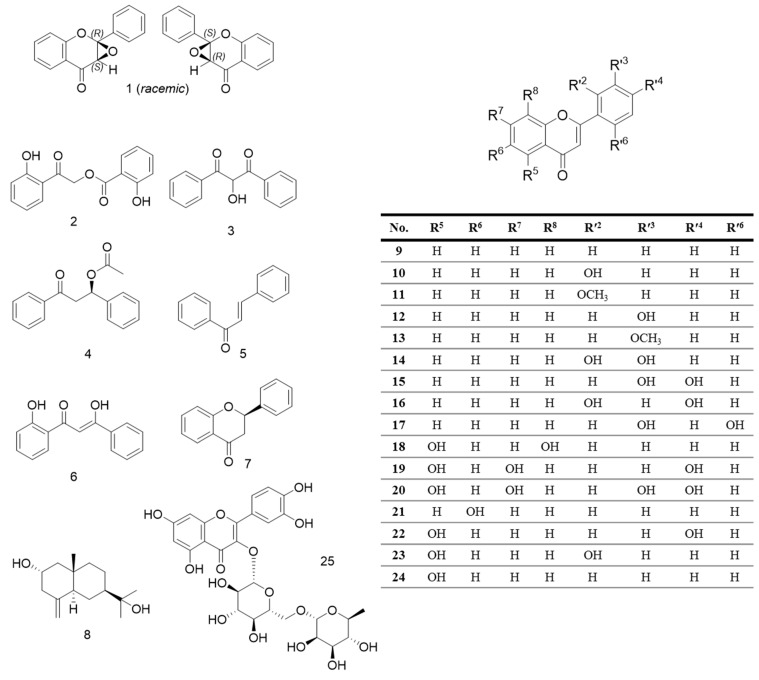
Structures of the isolated compounds from the aerial parts of *Dionysia diapensifolia*.

**Figure 2:**
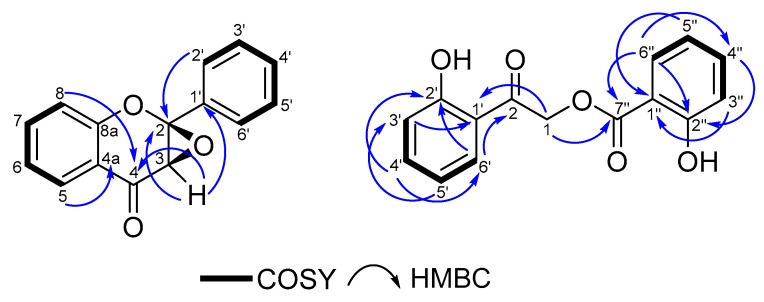
Key COSY and HMBC correlations of the two new phenolic compounds, **1** and **2**, from aerial part of *D. diapensifolia*.

Our preliminary screening and profiling of the methanolic extract of the aerial plant parts of *D. diapensifolia*, which exhibited a broad range of polyphenolic constituents, indicated potential NO release and iNOS and COX-2 expression inhibitory activity of subfractions of this plant. These results prompted us to further phytochemical investigations, which have led to the isolation of 25 compounds from the diethyl ether and *n*-butanol subfractions (Figure 1), including one new epoxy flavone **1** and two phenolic compounds **2** and **3**, which were identified as new natural products (Figure 1 and Figure 2), although **2** and **3** have been already known as synthetic substances [11,12]. Furthermore, compounds **5**, **7**, **8**, **12**–**18**, and **20**–**25** were isolated for the first time from this species. Structure elucidation of the isolated compounds was performed by evaluation of 1D- and 2D-NMR spectra and HR-MS data and in the case of the racemic compound **1**, by DP4+ NMR chemical shift probability calculations to determine the relative configuration at the chiral centers C2 and C3. In the next step, the anti-inflammatory activity of selected isolated compounds was investigated.

## 2. Materials and Methods

### 2.1. General Experimental Procedures

UV/VIS and ECD spectra measurements were performed at a J-8000 spectropolarimeter (JASCO, Tokio, Japan). Optical rotations were measured at a JASCO P-2000 polarimeter. IR spectra were recorded on an ALPHA FT-IR apparatus (Bruker, Ettlingen, Germany) equipped with a Platinum ATR module. One- and two-dimensional NMR experiments were performed at a Bruker Avance II 600 spectrometer operating at 600.19 MHz (^1^H) and 150.92 MHz (^13^C) at 300 K, with deuterated chloroform (chloroform-*d*), acetone (acetone-*d*_6_), methanol (methanol-*d*_4_) or DMSO (DMSO-*d*_6_) as solvents, containing 0.03% TMS. These solvents were purchased from Euriso-top SAS (Saint-Aubin Cedex, France). The chemical shifts were recorded as *δ* values referenced to the TMS signal. HPLC analysis was carried out at an LC-20AD XR system (Shimadzu, Düsseldorf, Germany) equipped with DAD detector, auto-sampler, and column thermostat. LC- and LC-ESI-MS parameters (general method): stationary phase: Phenomenex Aqua C18 5 µm, 250 × 4.6 mm; mobile phase: A = H_2_O + 0.02% TFA, B = acetonitrile; gradient: 0 min: B = 2%; 20 min: B = 50%; 40 min: B = 98%; 50 min B = 98%; T: 35 °C; flow: 1 mL/min; sample concentration and injection volume: 2–8 mg/mL, 5–15 µL in ACN, THF or MeOH. HPLC-ESIMS experiments were carried out at a HP1100 system (Agilent, Waldbronn, Germany) hyphenated to an Esquire 3000 plus ion trap (Bruker Daltonics, Bremen, Germany), using electrospray ionization (ESI) in alternating mode and using the following MS parameters: 1:5 split from HPLC, dry temp.: 325 °C; dry gas: 8.00 L/min; nebulizer 30 psi; full scan mode: *m*/*z* 100–1200; ion polarity: alternating mode; capillary voltage: 4.5 kV; end plate offset: −0.5 kV. HRESIMS experiments were performed at a Bruker TOF-Q mass spectrometer (Brucker Daltonics, Bremen, Germany) coupled with an Agilent 1200 HPLC, equipped with auto-sampler, DAD and column thermostat. ESIMS parameters: 1:5 split from HPLC, dry temp.: 220 °C; dry gas: 6.00 L/min; nebulizer 23.2 psi; full scan mode: *m*/*z* 100–1500; ion polarity: negative; capillary voltage: 3.5 kV; end plate offset: −0.5 kV. Semi-preparative HPLC experiments were conducted on a Dionex UltiMate 3000 (Thermo Fisher Scientific Inc., New York, NY, USA) equipped with an auto-sampler, DAD, column thermostat and AFC-3000 fraction collector. All reagents and solvents were of analytical grade and purchased from VWR International (Darmstadt, Germany) if not otherwise stated. Solvents used for HPLC analysis were obtained from Merck (Darmstadt, Germany). Ultrapure water for the HPLC analysis was produced by a Sartorius Arium 611 UV water purification system (Sartorius AG, Göttingen, Germany). Silica gel 60 (0.04–0.06 meshes) for column chromatography and Sephadex LH-20 were purchased from VWR International.

### 2.2. Plant Material and Chemicals

*Dionysia diapensifolia* aerial parts were collected at Arsanjan, Islam Abad village, Shiraz, Fars province, Iran. The plant material was dried at room temperature in the dark and stored in paper bags until extraction. Identification of plant material was performed by Asso. Prof. Dr. Hossein Batooli (Kashan Botanical Garden) and a voucher specimen (KBG: 3496) has been deposited in the Research Institute of Forests and Rangelands; Agricultural Research, Education and Extension Organization (AREEO, Tehran, Iran).

### 2.3. Extraction and Isolation

Plant material (500 g) was milled and extracted with 2 L of methanol using an ultrasonic bath (10 min). The filtrate was evaporated using a rotary evaporator set at 40 °C, resulting in 68 g of combined crude extract after seven further extractions. Subsequently, 67 g of methanolic extract were suspended in 400 mL of water and extracted with diethyl ether, ethyl acetate and *n*-butanol (5 × 200 mL of each). Evaporation of solvents resulted in following yields of each subfraction: 34 g diethyl ether, 5 g ethyl acetate, 20 g *n*-butanol and 10 g water fraction. Subsequently, a part of the diethyl ether subfraction (33 g) was fractionated by silica gel column (Ø = 10 cm, l = 40 cm) chromatography using a gradient elution of petroleum ether-EtOAc, from 0% to 50% EtOAc (500 mL, 5% increase in each step), followed by elution with 50, 65, 80 and 100% EtOAc (500 mL each). Elution was continued with 98:2, 95:5, 90:10 and 80:20 EtOAc:MeOH mixtures (each 500 mL). Fractions were monitored by TLC and subsequently combined to afford 27 fractions (F1–F27). All combined fractions were analyzed with HPLC-DAD and LC-MS. F4 (295 mg) was applied on a Sephadex LH-20 column (Ø = 2 cm, l = 100 cm) and eluted with CH_2_Cl_2_:acetone (85:15; *v*/*v*), which was resulted in 10 fractions (F4-S1 to F4-S10). Subsequently, F4-S5 (20 mg) was separated by semi-preparative HPLC using the following conditions: stationary phase: Phenomenex Synergi Max-RP 80Å 4 µm, 250 × 10 mm; mobile phase: A = acetonitrile, B = H_2_O; B = 80% for 15 min, then from B = 80% to B = 98% in 5 min; detection wavelength: 254 nm; flow rate: 3 mL/min; T: 30 °C, resulted in purification of F4-S5-P5 (**7**, 2.5 mg), F4-S5-P8 (**5**, 3 mg), F4-S5-P11 (**6**, 3.5 mg), and F4-S5-P13 (**3**, 2.5mg). F6 (550 mg) was subjected to RP18-MPLC using a Reveleris X2 flash chromatography system (Büchi Labortechnik AG, Flawil, Switzerland) with the following parameters: stationary phase: Reveleris C18 column 80 g, 40 µm; mobile phase: A = H_2_O, B = acetonitrile; gradient: 0 min: 0% B; 20 min: 100% B; 30 min: 100% B; 35 min: stop; flow: 20 mL/min; solid sample injection: trituration of sample with loose RP18 material (1:1); detection: ELSD, UV (254, 280 nm); collection mode: collect all; tube volume: 5 mL (peak), 20 mL (non-peak); UV and ELSD sensitivity: low. Obtained fractions were checked for their similarity using TLC and afforded F6-S5 (**22**, 15 mg) as pure compound. F8 (724 mg) was subjected to Sephadex LH-20 column chromatography (Ø = 2 cm, l = 100 cm) with isocratic elution (CH_2_Cl_2_: acetone; 85:15; *v*/*v*), which afforded 11 fractions (F8-S1 to F8-S11). Subsequently, F8-S7 (35 mg) and F8-S8 (30 mg) were subjected to semi preparative HPLC (conditions: Phenomenex Aqua 5 µm C18, 250 × 10 mm; mobile phase: A = acetonitrile, B = H_2_O; B = 50% to B = 98% in 15 min, then retained in B = 98% for 5 min; detection wavelength: 254 nm; flow rate: 3 mL/min; T: 30 °C) to afford F8-S7-P2 (**4**, 10 mg), F8-S7-P9 (**1**, 1.2 mg), as well as F8-S8-P9 (**2**, 3 mg) and F8-S8-P11 (**24**, 4 mg), respectively. F16 (250 mg) was chromatographed through a Sephadex LH-20 column (Ø = 2 cm, l = 100 cm) and eluted with dimethoxyethane as mobile phase to afford 5 fractions (F16-S1 to F16-S5). Further separation of F16-S5 (35 mg) using semi preparative HPLC (conditions: Phenomenex Aqua 5 µm C18, 250 × 10 mm; mobile phase: A = acetonitrile, B = H2O; B = 60% to B = 98% in 35 min, continued for 5 min; detection wavelength: 254 nm; flow rate: 2 mL/min; T: 30 °C), resulted in isolation of F16-S1-P1 (**18**, 4 mg), F16-S1-P2 (**9**, 5 mg), F16-S1-P4 (**11**, 4 mg), F16-S1-P6 (**13**, 5 mg), and F16-S1-P7 (**23**, 2 mg). F17 (657 mg) was loaded on a Sephadex LH-20 column (Ø = 2 cm, l = 100 cm) with isocratic elution using dimethoxyethane as solvent, resulting in 15 fractions, which F17-S13 (**14**, 21 mg) and F17-S16 (**12**, 17 mg) were isolated as pure compounds. F18 (873 mg) was subjected on chromatography using Sephadex LH-20 column (Ø = 2 cm, l = 100 cm) with isocratic elution of dimethoxyethane. The separation afforded 31 fractions (F18-S1 to F18-S31), of which F18-S17 (**14**, 7.7 mg) and F18-F29 (**19**, 5 mg) were isolated as pure compounds. F18-S14 (12 mg) was loaded on a silica gel column (Ø = 0.5 cm, l = 40 cm) and eluted isocratically with CH_2_Cl_2_:acetone (85:15; *v*/*v*), which afforded F18-S14-P3 (**17**, 1.2 mg) and F18-S14-P22 (**8**, 3 mg). Subsequently, F18-S22 (25 mg) was purified using semi preparative HPLC with following conditions, stationary phase: Phenomenex Aqua 5 µm C18, 250 × 10 mm; mobile phase: A = acetonitrile, B = H_2_O; B = 20% to B = 55% in 20 min, then from B = 55% to B = 98% in 5 min and remained in this concentration for 10 min; detection wavelength: 254 nm; flow rate: 3 mL/min; T: 30 °C, which resulted in F18-S22-P1 (**20**, 5 mg). Furthermore, F18-S23 (25 mg) was subjected to semi preparative HPLC (stationary phase: Phenomenex Aqua 5 µm C18, 250 × 10 mm; mobile phase: A = acetonitrile, B = H_2_O; B = 20% to B = 55% in 20 min, then from B = 55% to B = 98% in 5 min and remained in this concentration for 10 min; detection wavelength: 254 nm; flow rate: 3.5 mL/min; T: 30 °C), to afford F18-S23-P5 (**21**, 8 mg). F20 (292 mg) was recrystallized from methanol to afford F20-S1 (**10**, 40 mg). Chromatography of F22 (300 mg) on a Sephadex LH-20 column (Ø = 2 cm, l = 100 cm) with isocratic elution of dimethoxyethane, resulted in 20 fractions, of which F22-S9 (**15**, 6 mg) was obtained as pure substance. F23 (619 mg) was applied on a Sephadex LH-20 column (Ø = 2 cm, l = 100 cm) and eluted with dimethoxyethane, which afforded 20 fractions with F23-S11 (**16**, 10 mg) being isolated as a pure compound. HPLC-DAD analysis of the butanolic subfraction of the methanolic extract of *D. diapensifolia* showed one major peak at R_T_ = 10.8 min (Figure S22, Supplementary Materials). Part of the fraction (1.0 g) was used for recrystallization in methanol resulting in pure compound **25** (800 mg) which was identified by NMR and MS and comparison with a rutin standard (Figure S22, Supplementary Materials).

### 2.4. Physical and Spectroscopic Data of the New Isolated Compounds from Aerial Part of Dionysia diapensifolia

Compound **1**: Light yellow gum; UV (CH_3_CN) *λ_max_* (log ε): 256 nm (3.26), 320 nm (2.74); IR *ν_max_* 2956, 2927, 2856, 1732, 1684, 1606, 1460, 1303 cm^−1^; ^1^H (600.19 MHz, CDCl_3_) and ^13^C NMR data (150.91 MHz, CDCl_3_) see Table 1; LC-HR-ESI-MS (*m*/*z*): 237.0577 [M − H]^−^ (calcd for C_15_H_11_O_3_, 237.577).

Compound **2**: Yellow amorphous powder; UV (CH_3_CN) *λ_max_* (log ε): 208 nm (4.15), 248 nm (3.81), 313 nm (3.35); IR *ν_max_* 2956, 2927, 2856, 1732, 1663, 1483, 1252, 1191, 1178, 1155, 746 cm^−1^; ^1^H (600.19 MHz, CDCl_3_) and ^13^C NMR data (150.92 MHz, CDCl_3_) see Table 1; LC-HR-ESI-MS (*m*/*z*): 295.0573 [M + Na]^+^ (calcd for C_15_H_12_O_5_Na, 295.0577).

### 2.5. Cell Based Assays

#### 2.5.1. J774A.1 Murine Macrophages Cell Line

J774A.1 murine monocyte macrophage cell line (American Type Culture Collection, Rockville, MD, USA), was grown adherent to Petri dishes with Dulbecco′s modified Eagle′s medium (DMEM) supplemented with 10% fetal bovine serum (FBS), 25 mM HEPES, 2 mM glutamine, 100 u/mL penicillin and 100 mg/mL streptomycin at 37 °C in a 5% CO_2_ atmosphere. J774A.1 macrophages were plated in 96 well plates (5 × 10^4^/well) and allowed to adhere before the treatments described as follows.

#### 2.5.2. Evaluation of Cytotoxic Activity

J774A.1 macrophages (5 × 10^4^/well) were plated on 96-well plates and, after the medium was replaced with fresh medium alone or containing serial dilutions of the methanolic extracts and subfractions (5–0.5 μg/mL), or isolated compounds (**1**–**4**, **6**, **8**–**11**; 50–5 µM) for 24 h. 3-(4,5-dimethyltiazol-2yl)−2,5-phenyl-2*H*-tetrazolium bromide (MTT) assay was used to assess cell viability as previously reported [13]. In brief, 25 µL of MTT (5 mg/mL) were added and the cells were then incubated for additional 3 h. Cells were then lysed and the dark blue crystals solubilized with 100 µl of a solution containing 50% (mL/L) N,N-dimethylformamide, 20% (mL/L) sodium dodecyl sulfate (SDS) adjusted to an pH of 4.5. The optical density (OD) of each well was measured with a microplate spectrophotometer (Titertek Multiskan, Dasit, Cornaredo, Milan, Italy) equipped with a 550 nm filter, used as the main absorbance and a 620 nm filter used as reference. Macrophages viability after treatment was calculated as: % cellular inhibition = 100 − (OD treated/OD control) × 100. Positive control: 6-mercaptopurine.

#### 2.5.3. Measurement of NO Release

NO levels were measured as nitrite NO2^−^, as index of NO released by J774A.1 macrophages culture medium 24 h after LPS treatment by Griess reaction, as previously reported [14]. In brief, after adhesion, the cellular medium of J774A.1 macrophages (5 × 10^4^/well; 96-well plate) was replaced with fresh medium alone or containing serial dilutions of the methanolic extract and subfractions of *D. diapensifolia* (5–0.5 μg/mL) or isolated compounds (50–5 µM) and incubated for 1 h and then co-exposed to LPS (1 μg/mL) for further 24 h. Thereafter, 100 µl of cell culture medium were mixed with 100 µl of Griess reagent (equal volumes of 1% (*w*/*v*) sulphanilamide in 5% (*v*/*v*) phosphoric acid and 0.1% (*w*/*v*) *N*-(1-napthyl)ethylenediamine dihydrochloride in water and incubated at room temperature for 10 min. Subsequently, the absorbance was measured at 550 nm in a microplate reader Titertek (Dasit, Cornaredo, Milan, Italy). The amount of NO_2_^−^ measured in the samples is expressed as μM concentration, calculated by using a sodium NO_2_^−^ standard curve.

#### 2.5.4. iNOS and COX-2 Determination by Cytofluorimetry

After treatment with extracts, fractions or compounds and LPS as described for NO evaluation, J774A.1 macrophages were collected, washed with phosphate buffered saline (PBS) and incubated in fixing solution (containing PBS, 2% FBS and 4% formaldehyde) at 4 °C for 20 min and subsequently in fix perm solution (containing PBS, 2% FBS, 4% formaldehyde and 0.1% Triton X) at 4 °C for 30 min. Anti-iNOS (BD Laboratories, Milan, Italy) or anti-COX-2 antibodies (BD Laboratories) were then added for 30 min. The secondary antibody, in fix perm solution, was added and then macrophages were evaluated using a fluorescence-activated cell sorting (FACSscan; Becton Dickinson, Milan, Italy) and elaborated with Cell Quest software as previously reported [15].

#### 2.5.5. Data Analysis

Data are reported as mean ± standard error of the mean (S.E.M.) values of at least three independent experiments, each triplicate. Statistical analysis was performed by analysis of variance test, and multiple comparisons were made by Bonferroni’s test by using Prism 5 (GraphPad Software, San Diego, CA, USA). *p* values lower than 0.05 were considered as significant.

## 3. Results and Discussion

### 3.1. Structure Elucidation

Compound **1** was isolated as light-yellow gum. The HRESIMS displayed a [M − H]^−^ ion at *m*/*z* 237.0577, indicating a molecular formula of C_15_H_10_O_3_ (calcd for C_15_H_9_O_3_^−^, 237.0557). The UV spectrum in CH_3_CN showed a maximum at 256 (3.26) and 320 (2.74) nm, indicating a flavonoid scaffold. The ^1^H-NMR and HSQC spectra displayed nine aromatic methine groups at *δ*_H_ 8.00 (H-8), 7.58 (H-2′,6′), 7.39 (H-3′,5′), 7.35 (H-4′), 7.35 (H-7), 7.00 (H-6), and 6.74 (H-5), and an aliphatic methine group at *δ*_H_ 4.22 (H-3). Analysis of the ^13^C-NMR spectrum revealed 15 carbons, including 10 aromatic methine carbons at *δ*_C_ 136.7 (C-7), 129.2 (C-4′), 129.1 (C-3′,5′), 126.7 (C-8), 125.9 (C-2′,6′), 122.0 (C-6), 119.0 (C-5), four quaternary carbons at *δ*_C_ 159.9 (C-8a), 140.8 (C-1′), 121.6 (C-4a) and 84.8 (C-2), and a carbonyl group at *δ*_C_ 187.5 (C-4). The COSY spectrum showed two spin systems: H-5/H-6/H-7/H-8 and H-2′, 6′/H-3′, 5′/H-4′. The HMBC spectrum showed a correlation from H-5 (*δ*_H_ 6.74) to a carbonyl group at *δ*_C_ 187.5 and from H-6 (*δ*_H_ 7.00) to C-4a (*δ*_C_ 121.6), resulting in the identification of a carbonyl group at position C-4 (Figure 2). Furthermore, a polar aliphatic methine group (H-3) at *δ*_H_ 4.22 displayed a correlation to C-4, C-2 and C-1′, which later established the position of a phenyl group at C-2. The HMBC correlation from H-2′ to C-2 and the lack of correlation from H-2′ to C-3, confirmed the position of C-2 and C-3. Due to the down field shift of the resonances of C-3 and C-2 and considering the molecular formula, the remaining coordination sites of the mentioned carbons (C-2 and C-3) were filled by an oxygen-bridge, introducing two chiral centers at C-2 and C-3. However, optical rotation measurement resulted in “zero rotation value” for this molecule, suggesting the presence of a racemate. As the NOESY spectrum measured for **1** was not conclusive to determine its relative configuration, DP4+ calculation was performed. The results indicated the presence of a *Z*-epoxy ring with probability of greater than 99.9% as correct isomer (Figure S24, Supplementary Materials). Therefore, the structure of **1** was thus deduced as a racemate of 2*R*,3*S*- and 2*S*,3*R*-epoxyflavone.

Compound **2** was isolated as yellow amorphous powder. Its HR-ESI-MS displayed a [M + Na]^+^ ion at *m*/*z* 295.0573, indicating a molecular formula of C_15_H_12_O_5_ (calcd. 295.0577 for C_15_H_12_O_5_Na). ^1^H NMR and HSQC spectra revealed eight methine protons at *δ*_H_ 8.01, 7.69, 7.55, 7.52, 7.05, 7.02, 6.98 and 6.95; two methylene protons at *δ*_H_ 5.64 and two downfield shifted proton signals at *δ*_H_ 11.59 and 10.39 of two stabilized hydroxyl groups. The ^13^C NMR spectrum indicated 15 carbons; among them two carbonyl carbons at *δ*_C_ 196.6 (C-2) and *δ*_C_ 169.4 (C-7′′); a methylene carbon at 65.4 (C-1) and four quaternary carbons at *δ*_C_ 162.6 (C-2′), 162.0 (C-2′′), 117.1 (C-1′) and 111.8 (C-1′′) were characteristic. The COSY spectrum displayed two spin systems corresponding to H-3′/H-4′/H-5′/H-6′ and H-3′′/H-4′′/H-5′′/H-6′′, in which the splitting pattern indicated the presence of two bi-substituted phenyl rings. Furthermore, the HMBC spectrum displayed correlations from H-5′ (*δ*_H_ 6.98) to C-3′ and C-1′; from H-3′ (7.05) to C-1′ and C5′ and from H-6′ (7.69) to C-2′, C-4′, and a carbonyl group C-2 (*δ*_C_ 196.6), which altogether revealed the *ortho*-hydroxy benzoyl group structure. A HMBC correlation from H-1 (*δ*_H_ 5.64) to C-2 (*δ*_C_ 196.6), C-1′ (*δ*_C_ 117.1) and C-7′′ (*δ*_C_ 169.4) confirmed the position of the methylene group. Subsequently, correlations from H-5′′ (*δ*_H_ 6.95) to C-3′′ and C-1′′; from H-3′′ (7.02) to C-1′′, C-5′′, and C-2′′ and from H-6′′ (8.01) to C-2′′, C-4′′, and the carbonyl group C-7′′ (*δ*_C_ 169.4) revealed the structure and position of the second bi-substituted *ortho*-hydroxy benzoyl group. Considering all observed connections and correlations, the structure of compound **2** was deduced as 2-(2′-hydroxy-phenyl)-2-oxoethyl 2′′-hydroxybenzoate.

**Figure 3:**
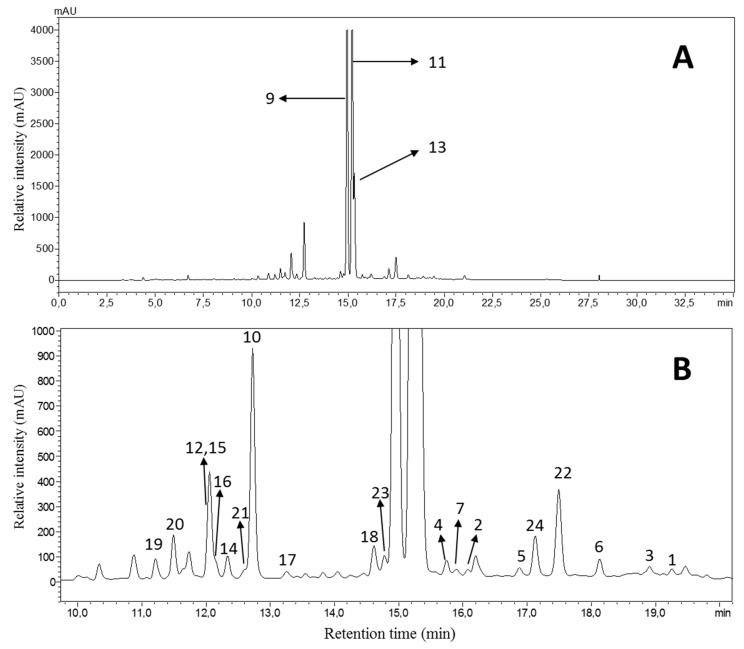
Chromatogram of the HPLC-DAD analysis of the diethyl ether subfraction (**A**) and an enlarged view (**B**) at λ 254 nm obtained from the methanolic extract of the aerial parts of *D. diapensifolia*. Analysis conditions: stationary phase: Phenomenex Aqua C18 5 µm, 250 × 4.6 mm; mobile phase: A = H_2_O + 0.02% TFA, B = acetonitrile; gradient: 0 min: B = 2%; 20 min: B = 50%; 40 min: B = 98%; 50 min B = 98%; T: 35 °C; flow: 1 mL/min; sample concentration and injection volume: 8 mg/mL, 10 µL.

In addition to compounds **1** and **2**, more than 20 constituents other could be isolated. Their structures were elucidated by 1D- and 2D-NMR techniques, along with mass spectrometry, and comparisons with literature data. Consequently these compounds were identified as 2-hydroxy-1,3-diphenylpropane-1,3-dione (**3**) [11], (*R*)-(+)-3-acetoxy-3-phenylpropiophenone (**4**) [16], chalcone (**5**) [14], 2’,β-dihydroxychalcone (**6**) [17], (+)-flavanone (**7**) [18], (+)-pterocarpol (**8**) [19], flavone (**9**) [18], 2’-hydroxyflavone (**10**) [20], 2’-methoxyflavone (**11**) [21], 3’-hydroxyflavone (**12**) [22], 3’-methoxyflavone (**13**) [21], 2’,3’-dihydroxy flavone (**14**) [22], 3’,4’-dihydroxyflavone (**15**) [22], 2’,4’-dihyroxyflavone (**16**) [22], 2’,5’-dihydroxyflavone (**17**) [20], primetin (**18**) [22], apigenin (**19**) [21], luteolin (**20**) [22], 6-hydroxyflavone (**21**) [22], 5,4’-dihydroxyflavone (**22**) [22], 5,2’-dihydroxyflavone (**23**) [23], primuletin (**24**) [22] and rutin (**25**). The ^1^H- and ^13^C-NMR spectra of all isolated compounds are shown in the Supplementary Materials. All isolated compounds (except **8** and **25**) were also detectable in the in HPLC-DAD chromatogram of the diethyl ether subfraction of *D. diapensifolia* (Figure 3).

### 3.2. Investigation of Anti-Inflammatory Activity of Subfractions and Isolated Compounds on NO Release, iNOS Expression and COX-2 Expression Inhibition on J774.A.1 Macrophages

Many chronic inflammations display elevated levels of inflammation mediators or their producing enzymes such as NO, iNOS and COX-2 [24]. NO production can be modulated by various inducers such as lipopolysaccharide (LPS), tumor necrosis factor alpha (TNF-α), and interleukin-1β (IL-1β). However, the transcription of iNOS is the major regulatory step of NO production [25]. It has also been shown that NO production modulates the expression of COX-2 through the activation of an inflammation cascade, leading to an up-regulation of COX-2 and increasing levels of PGE2 [26,27]. Considering the correlation between these mediators and their critical role in inflammation, inhibition of them seems a promising therapeutic strategy for treatment of many chronic inflammatory diseases.

The crude methanolic extract of *D. diapensifolia*, along with subfractions obtained by partitioning with solvents of different polarity, were tested for their cytotoxic activity on a J774A.1 macrophage cell line prior further pharmacological investigation. As shown in Table 2, the results indicated that all subfractions were not toxic in the applied concentration range, while the MeOH extract of D. *diapensifolia* was slightly toxic on J774A.1 macrophages at higher concentrations (Table 2).

Subsequent inhibitory activity assessment of the subfractions on NO production revealed the diethyl ether subfraction as most active subfraction, which could reduce the NO release by more than 70% in the applied concentration range (*p* < 0.001 vs. LPS alone, Table 3). Furthermore, evaluation of the iNOS inhibitory activity indicated that all subfractions significantly reduced iNOS release in a concentration dependent manner (*p* < 0.001 vs. LPS alone, Table 3). However, the diethyl ether subfraction displayed a lower activity than other tested fractions. As shown in Table 3, the ability to inhibit the COX-2 expression was only moderate with a maximum of 31.8% for the diethyl ether fraction (concentration of 5 µg/mL, *p* < 0.01 vs. LPS).

**Figure 4:**
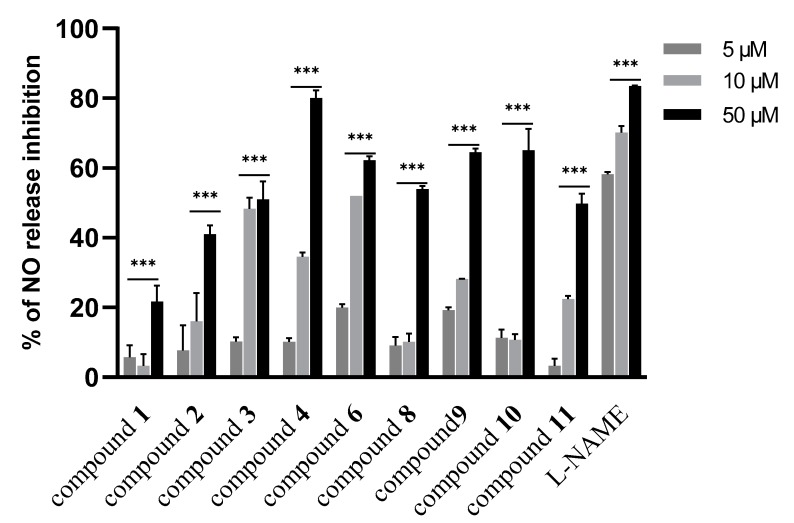
Effect of isolated compounds from aerial parts of *Dionysia diapensifolia* on NO release inhibition in J774A.1 macrophages (n = 3). Data are expressed as mean ± S.E.M.; *** denotes *p* < 0.001 vs. LPS (1 µg/mL). L-NAME (5, 10, and 50 µM) was used as a positive control.

In the next step, the isolated new compounds (**1**–**3**) along with compounds **4**, **6**, **8**, **9**, **10** and **11** were selected for further investigation. The selection of those known compounds was based on the lack of pharmacological activity in inflammation or in the case of **9**–**11**, due to their contribution as major constituents of the subfraction. First, the cytotoxic activity of selected isolated compounds was assessed. Results illustrated that only compounds **6** and **10** at concentrations above 50 µM were growth-inhibitory (Table S1, Supplementary Materials). Subsequently, all the selected compounds were investigated for their effect on NO release, as well as on iNOS and COX-2 expression levels. As shown in Figure 4, the results indicated that compound **4** was the most active compound in NO release inhibition at all tested concentrations (5–50 µM) in a concentration-dependent manner. However, other investigated compounds displayed moderate activity in this assay.

**Figure 5:**
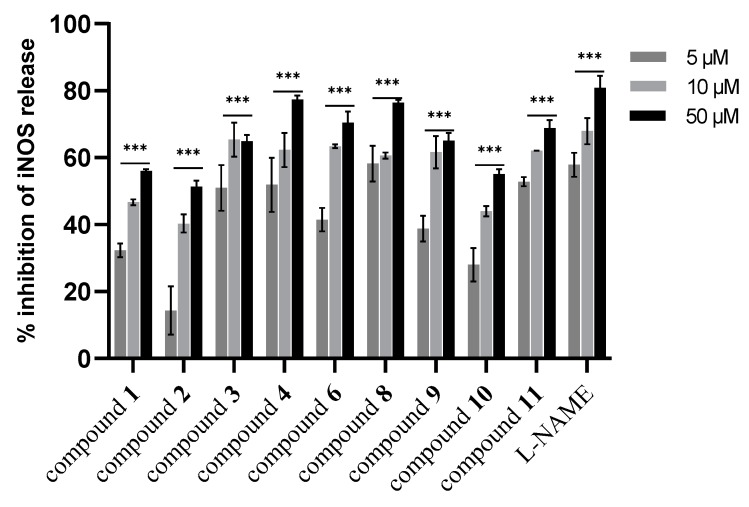
Effect of isolated compounds from aerial parts of *Dionysia diapensifolia* on iNOS expression in J774A.1 macrophages (n = 3). Data are expressed as mean ± S.E.M.; *** denotes *p* < 0.001 vs. LPS (1 µg/mL). L-NAME (5, 10, and 50 µM) was used as a positive control.

**Figure 6:**
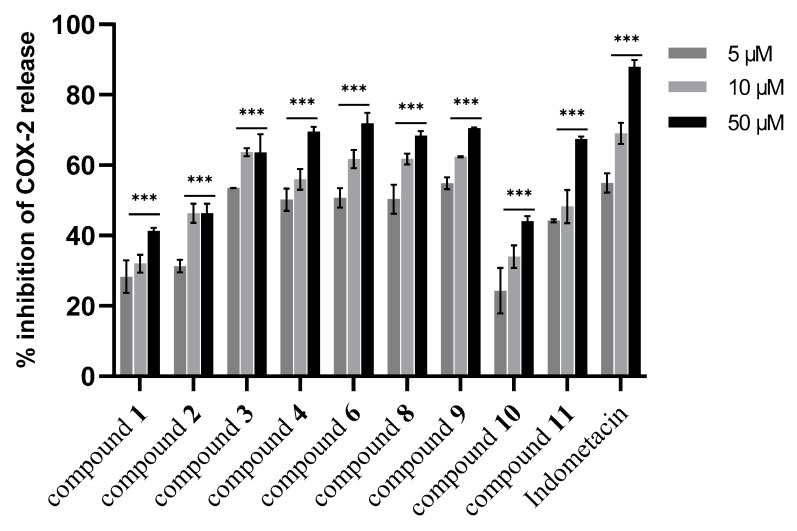
Effect of isolated compounds from aerial parts of *Dionysia diapensifolia* on COX-2 expression in J774A.1 macrophages (n = 3). Data are expressed as mean ± S.E.M.; *** denotes *p* < 0.001 vs. LPS (1 µg/mL). Indomethacin (5, 10, and 50 µM) was used as a positive control.

Further investigation on the effect on iNOS expression levels showed that compounds **4**, **6**, **8**, **11** caused a comparable reduction of iNOS expression like L-NAME in a concentration dependent manner (Figure 5). In this regard, compounds were assessed further for their impact on COX-2 expression. As shown in Figure 6, almost all tested compounds (except **1**, **2**, and **10**) revealed a significant COX-2 expression reduction (*p* < 0.001 vs. LPS) in comparison to indomethacin (positive control) and in all tested concentrations (5–50 µM). In comparison, compound **1** and **2** moderately inhibited the COX-2 expression.

*Dionysia* species are mainly known for their exudate flavonoids, covering a broad range of diverse structural features [8,16]. Previous studies showed the presence of not only regular flavonoids, but also irregular and aberrant flavonoids with unusual substitution patterns such as 2’-hydroxyflavone, 3’,4’-dihydroxyflavone and 8,2’-dihydroxyflavone [8]. Investigations of exudate flavonoids of *D. diapensifolia* led to the identification of 2’,β-dihydroxychalcone (**6**), flavone (**9**), 2’-hydroxyflavone (**10**), 2’-methoxyflavone (**11**), 3’-methoxy-4’,5’-methylendioxyflavone, 5-hydroxy-2’-methoxyflavone, apigenin, apigenin-7-methylether, naringenin (**19**), naringenin-7-methyl ether, kaempferol, kaempferol-3-methylether, kaempferol-7-methylether and the isolation of an unusual dihydrochalcone (*R*)-(+)-3-acetoxy-3-phenyl-propiophenone (**4**) for the first time from a *Dionysia* species [10,16]. However, in this study, our phytochemical investigation led to the isolation of a new natural product **1** and two compounds **2**, **3** so far only known as synthetic products, which can be classified as unusual flavonoids and phenolic compounds. Moreover, in the present study, the sesquiterpenoid **8** is reported for the first time from the genus *Dionysia* and the Primulaceae family.

**Figure 7:**
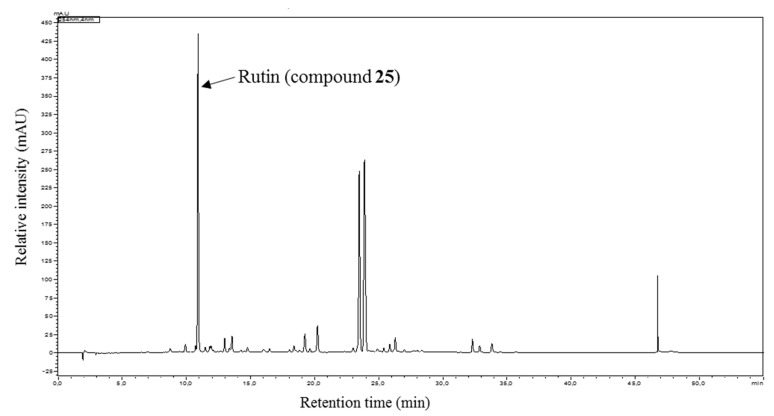
HPLC-DAD chromatogram of methanolic extract of the aerial parts of *D. diapensifolia*. Analysis condition: stationary phase: Phenomenex Synergi Max-RP C18 4 µm, 150 × 4.6 mm; mobile Phase: A = H_2_O + 0.02% TFA, B = acetonitrile; gradient: 0 min: B = 2%; 20 min: B = 98%; 35 min: B = 98%; T: 35 °C; flow: 1 mL/min; sample concentration and injection volume: 2 mg/mL, 10 µL; detection = 254 nm.

Since the HPLC analysis of the crude methanolic extract of *D. diapensifolia* showed not only two major peaks for compound **9** and **11** (co-eluting with **13**, Figure 7) but also a large peak at R_T_ 10.8 min, which was mainly concentrated in the obtained *n*-butanol fraction (Figure S22, Supplementary Materials). Purification and structure elucidation of a part of the *n*-butanol fraction led to identification of rutin (**25**) for the first time in the methanolic extract of *D. diapensifolia*.

Most of the known isolated compounds were described previously for their anti-inflammatory activity. Wang et al. showed that 6-hydroxyflavone (**21**) and its derivatives could potentially inhibit the downstream iNOS expression in kidney mesangial cells [28]. Luteolin (**20**), a natural flavone known as potent anti-inflammatory agent, found in many plant species, indicated to reduce TNF-α and proinflammatory cytokines expression, including that of TNF-α, IL-6 and IL-1β in RAW264.7 [29]. Apigenin (**19**) is a multitarget compound with promising anti-inflammatory activity. It has been reported that apigenin could suppress LPS induced nitric oxide (NO) and cyclooxygenase-2 (COX-2) expression in RAW 264.7 macrophage cells. In addition, it can attenuate acute lung injury (ALI) through inhibition of COX-2 and NF-kB activation pathways [30]. Furthermore, apigenin could significantly inhibit production of IL-1β and down regulated iNOS and COX-2 expression in a murine DSS colitis model [30]. Monohydroxy dibenzoylmethane (**3**), as curcumin structure analogue, displayed high inhibitory activity on chemical induced tumor promotion and inflammation on mouse skin. Furthermore, it induced apoptosis in human colorectal carcinoma cells through sequential activation of caspase cascades [31]. Considering previously published data along with our findings presented in this study qualify *Dionysia diapensifolia* and related species as valuable sources of flavonoids and chalcones with high anti-inflammatory capacity.

## 4. Conclusions

In this study, 25 compounds (19 flavonoids, five phenolic compounds and a sesquiterpenoid) were isolated from the aerial part of *D. diapensifolia*, of which three (compounds **1**–**3**) were new natural products and one (compound **8**) was found to be a new constituent of the *Dionysia* genus and the Primulaceae family. Among the known isolates, compounds **6**, **9**, **10**, **11**, **12** and **19** were already identified from exudates of *D. diapensifolia*, while the remaining compounds are reported for the mentioned species for the first time in this study. Since NOESY correlations were not conclusive, determination of the relative configuration of compound **1** was performed by DP4+ chemical shift calculations to establish the relative configuration of chiral centers C-2 and C-3. Furthermore, our results indicated that *D. diapensifolia* subfractions display remarkable anti-inflammatory activities through suppression of the release of inflammatory mediators such as NO and the reduction of the iNOS and COX-2 expression. Although a traditional use of this plant has not been reported clearly in literature, it might be used as a rich and cheap source of anti-inflammatory compounds, which can be used in the medicine and cosmetic industries.

## Figures and Tables

**Table 1:** ^1^H- and ^13^C-NMR Spectroscopic Data of **1** and **2** in CDCl_3_ (^1^H 600.19 MHz, ^13^C 150.91 MHz).

Compound 1			Compound 2		
Position	δ_C_ (ppm)	δ_H_ (ppm, J in Hz)	Position	δ_C_ (ppm)	δ_H_ (ppm, J in Hz)
1	-	-	1	65.4	5.64 (2H, s)
2	84.8	-	2	196.3	-
3	56.2	4.22 (1H, s)	1′	117.2	-
4	187.5	-	2′	162.6	-
4a	121.6	-	3′	119.2	7.05 (1H, d, *J* =8.5)
5	119.0	6.74 (1H, dd, *J* = 8.4, 0.6)	4′	137.4	7.55 (1H, ddd, *J* = 8.1, 6.8, 1.2)
6	122.0	7.0 (1H, ddd, *J* = 8.4, 7.7, 0.8)	5′	119.7	6.98 (1H, m)
7	136.7	7.35 (2H, m)	6′	128.4	7.69 (1H, dd, *J* = 8.0, 1.6)
8	126.7	8.0 (1H, dd, *J* = 7.8, 1.7)	1′′	111.8	-
8a	159.9	-	2′′	162.0	-
1′	140.8	-	3′′	117.9	7.02 (1H, d, J = 8.4)
2′	125.9	7.58 (2H, d, *J* = 8.5)	4′′	136.5	7.51 (1H, ddd, *J* = 9.3, 6.9, 1.3)
3′	129.1	7.39 (2H, t, *J* = 7.7)	5′′	119.6	6.95 (1H, m)
4′	129.2	7.35 (2H, m)	6′′	130.4	8.01 (1H, dd, *J* = 8.0, 1.7)
5′	129.1	7.39 (2H, t, *J* = 7.7)	7′′	169.4	-
6′	125.9	7.58 (2H, d, *J* = 8.5)	OH-2′	-	11.59
			OH-2′′	-	10.39

**Table 2:** % Cellular inhibition of subfractions of the methanolic extract of *D. diapensifolia* on macrophages after 24 h, evaluated by MTT assay. Data are expressed as mean ± S.E.M (n = 3) of the percentage of cellular mortality vs. control cells. **, * denote *p* < 0.01, *p* < 0.05 vs. control.

Test Substance	5 µg/mL	2.5 µg/mL	0.5 µg/mL	0.05 µg/mL
MeOH extract	34.33 ± 0.88 **	29.67 ± 0.88 **	13.33 ± 0.68 *	2.33 ± 0.88
Diethyl ether	3.03 ± 1.85	1.13 ± 1.13	1.47 ± 0.82	4.53 ± 2.54
EtOAc	0.00 ± 0.00	0.00 ± 0.00	0.00 ± 0.00	3.47 ± 2.61
BuOH	8.03 ± 0.60	2.63 ± 1.56	3.33 ± 3.33	2.23 ± 2.23
H_2_O	0.57 ± 0.38	0.23 ± 0.23	0.33 ± 0.33	2.33 ± 1.25
6-mercaptopurin ^1^	27.86 ± 1.88 **	17.65 ± 0.91 **	13.23 ± 2.12 *	-

^1^ Concentrations of positive control: 200, 50 and 10 µM, respectively.

**Table 3:** Effect of tested of the subfractions of *D. diapensifolia*. on NO release, iNOS and COX-2 expression. Data are expressed mean ± S.E.M of the percentage of inhibition vs. LPS (n = 3). ***, **, * denote *p* < 0.001, *p* < 0.01, *p* < 0.05 vs. LPS.

	% Inhibition of NO Release			% Inhibition of iNOS Expression			% Inhibition of COX-2 Expression		
	5 µg/mL	2.5 µg/mL	0.5 µg/mL	5 µg/mL	2.5 µg/mL	0.5 µg/mL	5 µg/mL	2.5 µg/mL	0.5 µg/mL
Diethyl ether	78.91 ± 0.10 ***	78.99 ± 0.00 ***	73.22 ± 2.12 ***	59.85 ± 8.95 ***	59.60 ± 1.90 ***	51.06 ± 2.44 ***	31.80 ± 0.36 **	21.65 ± 3.13 *	13.48 ± 5.13
EtOAc	71.23 ± 0.17 ***	64.53 ± 1.87 ***	60.85 ± 1.05 ***	72.51 ± 0.39 ***	57.50 ± 2.59 ***	44.34 ± 2.26 ***	27.29 ± 0.97 **	20.19 ± 0.35 *	10.15 ± 4.73
*n*-BuOH	70.12 ± 0.81 ***	61.08 ± 0.62 ***	60.21 ± 0.88 ***	73.02 ± 0.73 ***	62.15 ± 4.27 ***	56.80 ± 0.96 ***	24.97 ± 1.16 **	14.71 ± 4.28	15.36 ± 0.16
H_2_O	67.21 ± 0.22 ***	59.12 ± 1.98 ***	57.31 ± 2.00 ***	68.38 ± 3.16 ***	61.27 ± 0.37 ***	54.02 ± 0.71 ***	25.86 ± 0.48 **	17.85 ± 0.68	12.27 ± 1.41
Positive control ^1,2^	69.64 ± 0.61 ***	54.81 ± 0.98 ***	34.40 ± 0.00 **	56.98 ± 0.98 ***	36.07 ± 1.95 ***	21.94 ± 0.24 **	54.07 ± 0.71 ***	47.18 ± 1.88 ***	12.87 ± 1.60 **

^1^ L-NAME used as positive control for NO and iNOS assays (concentrations: 5, 2.5 and 0.5 µM) and ^2^ indomethacin for COX-2 assay (concentrations: 5, 2.5 and 0.5 µM).

## Supplementary Materials

The following are available online at https://www.mdpi.com/2218-273X/10/3/438/s1, ^1^H, COSY, HSQC, HMBC, ^13^C, IR and HRLCESIMS spectra of compound **1** and **2**. HPLC chromatogram of *n*-butanol fraction of the MeOH extract. Results of cytotoxic activity of methanolic extract and its subfractions. Results of DP4+ chemical shift probability calculation. Cytotoxic activity of subfractions of MeOH extract of *D. diapensifolia*. Cytotoxic activity of selected compounds in the study. Low energy conformers of two diastereomers of compound **1** and their Boltzmann contribution. Experimental and calculated chemical shifts and shielding tensors, used for DP4+ chemical shift probability calculation. Results of DP4+ chemical shift probability calculation.
