# Supplementary material for: Unusual Secondary Metabolites of the Aerial Parts of Dionysia diapensifolia Bioss. (Primulaceae) and Their Anti-Inflammatory Activity
Source: Biomolecules. 2020 Mar 12;10(3):438. doi: 10.3390/biom10030438 (PMC7175200; doi:10.3390/biom10030438)
Supplement: Supplementary file 1 [file biomolecules-10-00438-s001.pdf]

Supplementary Materials

Unusual Secondary Metabolites of the Aerial Parts of *Dionysia diapensifolia* Bioss. (Primulaceae) and Their Anti-Inflammatory Activity

Mostafa Alilou [1,2], Stefania Marzocco [3,*], Hossein Batooli [4], Jakob Troppmair [2], Stefan Schwaiger [1,*] and Hermann Stuppner [1]

1 Institute of Pharmacy, Pharmacognosy, Center for Molecular Biosciences (CMBI), University of Innsbruck, 6020 Innsbruck, Austria; mostafa.alilou@student.uibk.ac.at (M.A.); hermann.stuppner@uibk.ac.at (H.S.)
2 Daniel-Swarovski Research Laboratory, Department of Visceral, Transplant and Thoracic Surgery, Innsbruck Medical University, Innrain 66, 6020 Innsbruck, Austria; jakob.troppmair@i-med.ac.at
3 Department of Pharmacy, University of Salerno, Via Giovanni Paolo II 132, I-84084 Fisciano SA, Italy
4 Kashan Botanical Garden, Research Institute of Forests and Rangelands; Agricultural Research, Education and Extension Organization (AREEO), 193951113 Tehran , Iran; ho_batooli@yahoo.com
4
* Correspondence: smarzocco@unisa.it (S.M.); stefan.schwaiger@uibk.ac.at (S.S.); Tel.: +39-089-969250 (S.M.); +43-512-507-58406 (S.S.)

INDEX

Figure S1. ^{1}H-NMR (600.19 MHz, $CHCl_3$-*d*) of compound **1**. 4

Figure S2. ^{1}H-NMR (600.19 MHz, $CHCl_3$-*d*) of compound **1** (insert *δ* 8.0 – 6.5 ppm). 5

Figure S3. COSY spectrum (600.19 MHz, $CHCl_3$-*d*) of compound **1**. 6

Figure S4. HSQC spectrum (600.19/150.91 MHz, $CHCl_3$-*d*) of compound **1**. 7

Figure S5. HSQC spectrum (600.19/150.91 MHz, $CHCl_3$-*d*) of compound **1** (insert *δ* 8.0 – 6.5 ppm). 8

Figure S6. HMBC spectrum (600.19/150.91 MHz, $CHCl_3$-*d*) of compound **1**. 9

Figure S7. HSQC spectrum (600.19/150.91 MHz, $CHCl_3$-*d*) of compound **1** (insert *δ* 8.0 – 4.0 ppm). 10

Figure S8. ^{13}C-NMR spectrum (150.91 MHz, $CHCl_3$-*d*) of compound **1**. 11

Figure S9. IR spectrum of compound **1**. 12

Figure S10. HRLCESIMS spectrum of compound **1**. 13

Figure S11. ^{1}H-NMR (600.19 MHz, $CHCl_3$-*d*) of compound **2**. 14

Figure S12. ^{1}H-NMR (600.19 MHz, $CHCl_3$-*d*) of compound **2** (insert δ 8.2 – 6.7 ppm). 15

Figure S13. COSY spectrum (600.19 MHz, $CHCl_3$-*d*) of compound **2**. 16

Figure S14. COSY spectrum (600.19 MHz, $CHCl_3$-*d*) of compound **2** (insert δ 8.2 – 6.7 ppm) 17

Figure S15. HSQC spectrum (600.19/150.91 MHz, $CHCl_3$-*d*) of compound **2**. 18

Figure S16. HSQC spectrum (600.19/150.91 MHz, $CHCl_3$-*d*) of compound **2** (insert δ 8.2 – 5.4 ppm). 19

Figure S17. HMBC spectrum (600.19/150.91 MHz, $CHCl_3$-*d*) of compound **2**. 20

Figure S18. HMBC spectrum (600.19/150.91 MHz, $CHCl_3$-*d*) of compound **2** (insert δ 11.7 – 5.5 ppm). 21

Figure S19. ^{13}C-NMR (600.19/150.91 MHz, $CHCl_3$-*d*) of compound **2**. 22
Figure S20. IR spectrum of compound **2**. 23
Figure S21. HRLCESIMS spectrum of compound **2**. 24
Figure S22. ^{1}H-NMR (600.19 MHz, $CHCl_3$-*d*) of compound **3**. 25
Figure S23. COSY spectrum (600.19 MHz, $CHCl_3$-*d*) of compound **3**. 26
Figure S24. HSQC spectrum (600.19, 150.91 MHz, $CHCl_3$-*d*) of compound **3**. 27
Figure S25. HMBC spectrum (600.19, 150.91 MHz, $CHCl_3$-*d*) of compound **3**. 28
Figure S26. ^{13}C-NMR (150.91 MHz, $CHCl_3$-*d*) of compound **3**. 29
Figure S27. ^{1}H-NMR (600.19 MHz, $CHCl_3$-*d*) of compound **4**. 30
Figure S28. ^{13}C-NMR (150.91 MHz, $CHCl_3$-*d*) of compound **4**. 31
Figure S29. ^{1}H-NMR (600.19 MHz, $CHCl_3$-*d*) of compound **5**. 32
Figure S30. ^{13}C-NMR (150.91 MHz, $CHCl_3$-*d*) of compound **5**. 33
Figure S31. ^{1}H-NMR (600.19 MHz, $CHCl_3$-*d*) of compound **6**. 34
Figure S32. ^{13}C-NMR (150.91 MHz, $CHCl_3$-*d*) of compound **6**. 35
Figure S33. ^{1}H-NMR (600.19 MHz, $CHCl_3$-*d*) of compound **7**. 36
Figure S34. ^{13}C-NMR (150.91 MHz, $CHCl_3$-*d*) of compound **7**. 37
Figure S35. ^{1}H-NMR (600.19 MHz, $CHCl_3$-*d*) of compound **8**. 38
Figure S36. ^{13}C-NMR (150.91 MHz, $CHCl_3$-*d*) of compound **8**. 39
Figure S37. ^{1}H-NMR (600.19 MHz, MeOH-d_4) of compound **9**. 40
Figure S38. ^{13}C-NMR (150.91 MHz, MeOH-d_4) of compound **9**. 41
Figure S39. ^{1}H-NMR (600.19 MHz, DMSO-d_6) of compound **10**. 42
Figure S40. ^{13}C-NMR (150.91 MHz, DMSO-d_6) of compound **10**. 43
Figure S41. ^{1}H-NMR (600.19 MHz, MeOH-d_4) of compound **11**. 44
Figure S42. ^{13}C-NMR (150.91 MHz, MeOH-d_4) of compound **11**. 45
Figure S43. ^{1}H-NMR (600.19 MHz, MeOH-d_4) of compound **12**. 46
Figure S44. ^{13}C-NMR (150.91 MHz, MeOH-d_4) of compound **12**. 47
Figure S45. ^{1}H-NMR (600.19 MHz, MeOH-d_4) of compound **13**. 48
Figure S46. ^{13}C-NMR (150.91 MHz, MeOH-d_4) of compound **13**. 49
Figure S47. ^{1}H-NMR (600.19 MHz, Acetone-d_6) of compound **14**. 50
Figure S48. ^{13}C-NMR (150.91 MHz, Acetone-d_6) of compound **14**. 51
Figure S49. ^{1}H-NMR (600.19 MHz, DMSO-d_6) of compound **15**. 52
Figure S50. ^{13}C-NMR (150.91 MHz, DMSO-d_6) of compound **15**. 53
Figure S51. ^{1}H-NMR (600.19 MHz, DMSO-d_6) of compound **16**. 54
Figure S52. ^{13}C-NMR (150.91 MHz, DMSO-d_6) of compound **16**. 55
Figure S53. ^{1}H-NMR (600.19 MHz, $CHCl_3$-*d*) of compound **17**. 56

Figure S54. ^{13}C-NMR (150.91 MHz, $CHCl_3$-*d*) of compound **17**. 57

Figure S55. ^{1}H-NMR (600.19 MHz, $CHCl_3$-*d*) of compound **18**. 58

Figure S56. ^{13}C-NMR (150.91 MHz, $CHCl_3$-*d*) of compound **18**. 59

Figure S57. ^{1}H-NMR (600.19 MHz, DMSO-d_6) of compound **19**. 60

Figure S58. ^{13}C-NMR (150.91 MHz, DMSO-d_6) of compound **19**. 61

Figure S59. ^{1}H-NMR (600.19 MHz, DMSO-d_6) of compound **20**. 62

Figure S60. ^{13}C-NMR (150.91 MHz, DMSO-d_6) of compound **20**. 63

Figure S61. ^{1}H-NMR (600.19 MHz, MeOH-d_4) of compound **21**. 64

Figure S62. ^{13}C-NMR (150.91 MHz, MeOH-d_4) of compound **21**. 65

Figure S63. ^{1}H-NMR (600.19 MHz, $CHCl_3$-*d*) of compound **22**. 66

Figure S64. ^{13}C-NMR (150.91 MHz, $CHCl_3$-*d*) of compound **22**. 67

Figure S65. ^{1}H-NMR (600.19 MHz, Acetone-d_6) of compound **23**. 68

Figure S66. ^{13}C-NMR (150.91 MHz, Acetone-d_6) of compound **23**. 69

Figure S67. ^{1}H-NMR (600.19 MHz, MeOH-d_4) of compound **24**. 70

Figure S68. ^{13}C-NMR (150.91 MHz, MeOH-d_4) of compound **24**. 71

Figure S69. ^{1}H-NMR (600.19 MHz, DMSO-d_6) of compound **25**. 72

Figure S70. ^{13}C-NMR (150.91 MHz, DMSO-d_6) of compound **25**. 73

Table S1. Anti-proliferative activity of selected compounds isolated from diethyl ether subfraction of the methanolic extract of *D. diapensifolia* in MTT assay after 24 h shown as % cellular inhibition (MEAN±SEM) of n = 3. ***, **, * denote $p < 0.001$, $p < 0.01$, $p < 0.05$ vs. control. 74

Figure S71. HPLC-DAD chromatogram of *D. diapensifolia n*-butanol subfraction and rutin standard. 75

Figure S72. Low energy conformers of compound **1**. Conformer generation was done on MacroModel 09 (Schrödinger Ltd.), using OPLS-3 as forcefield in gas phase. Conformers occurring in energy window of 5 kcal.mol^{-1} were further optimized in DFT/6-31G(d,p) level in the gas phase using Gaussian 16 v. A3 software [1] 76

Table S2. Experimental chemical shifts and Boltzmann-averaged shielding tensors of two diastereomers of **1**, used for DP4+ chemical shift calculation. 77

Figure S73. The result sheet of DP4+ chemical shift probability calculation of two diastereomers of compound **1**. Calculation of shift tensors were done using GIAO/mpw1pw91/6-311+(d,p)/CPCM in $CHCl_3$ in Gaussian 16 A.3 [Ref]. DP4+ probability calculation was done using the method originally published by Grimblat et. al [2]. 78

References 79

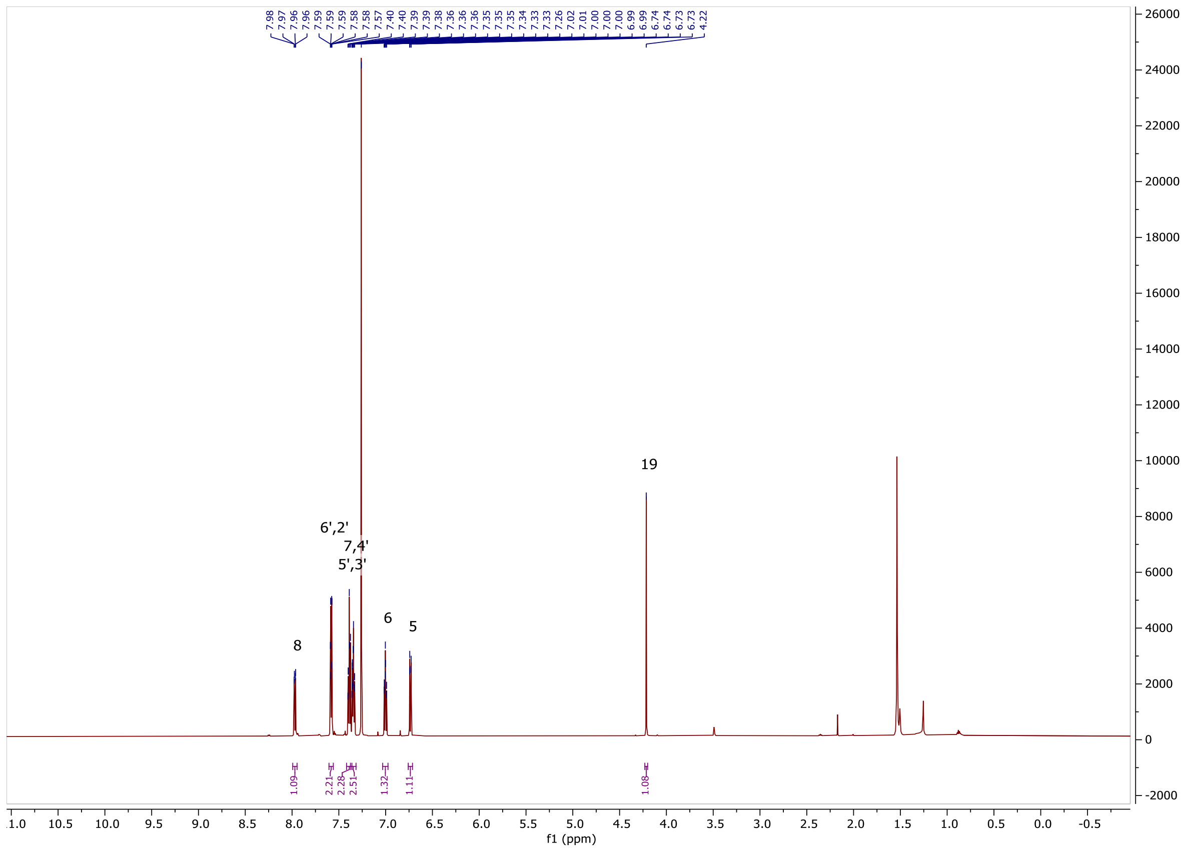

Figure S1. ^{1}H-NMR (600.19 MHz, $CHCl_3$-*d*) of compound 1.

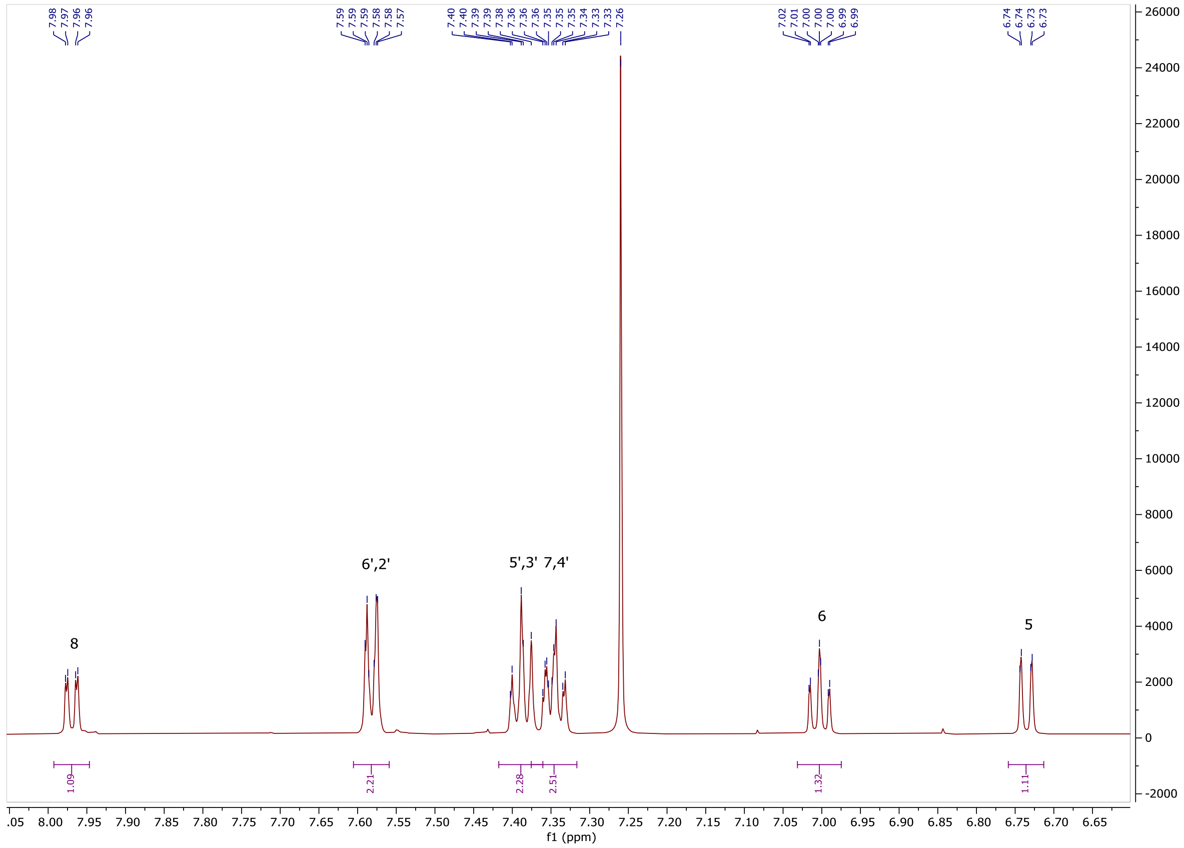

Figure S2. ¹H-NMR (600.19 MHz, CHCl₃-*d*) of compound 1 (insert *δ* 8.0 – 6.5 ppm).

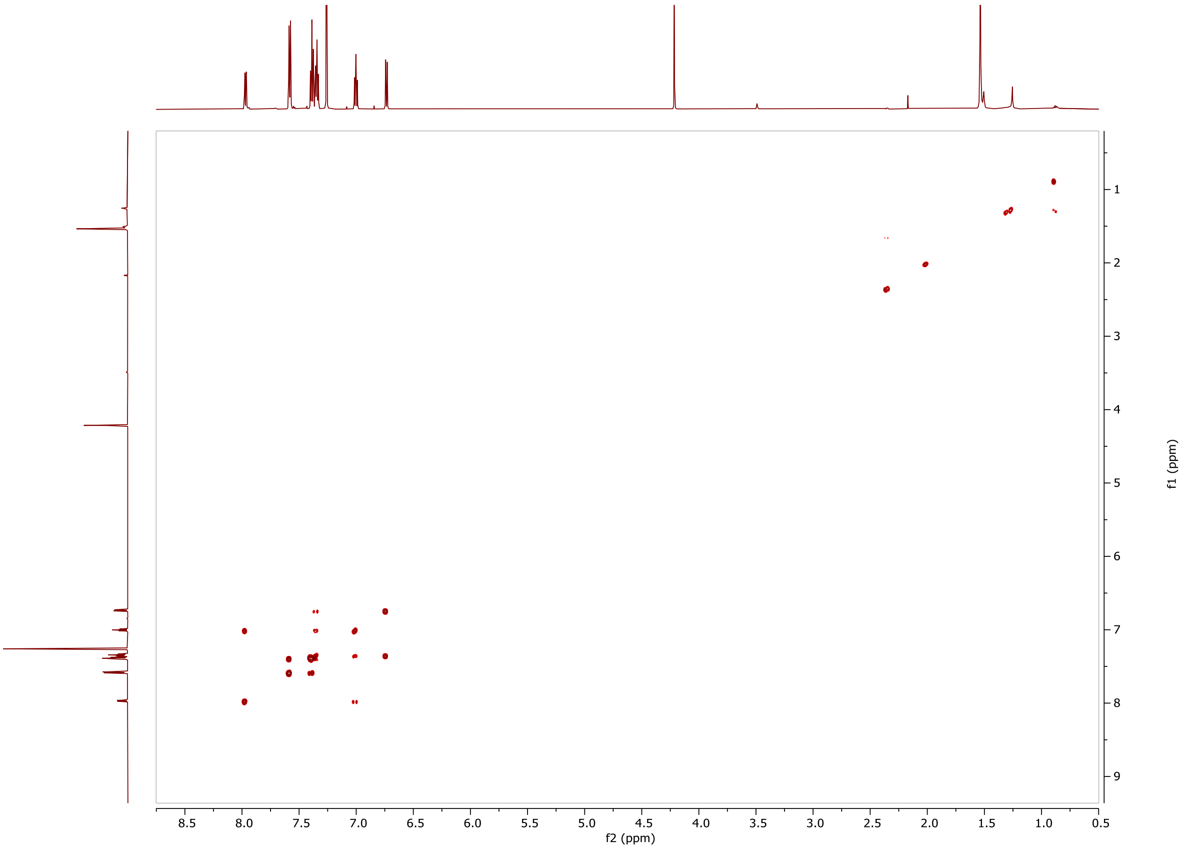

Figure S3. COSY spectrum (600.19 MHz, CHCl3-*d*) of compound 1.

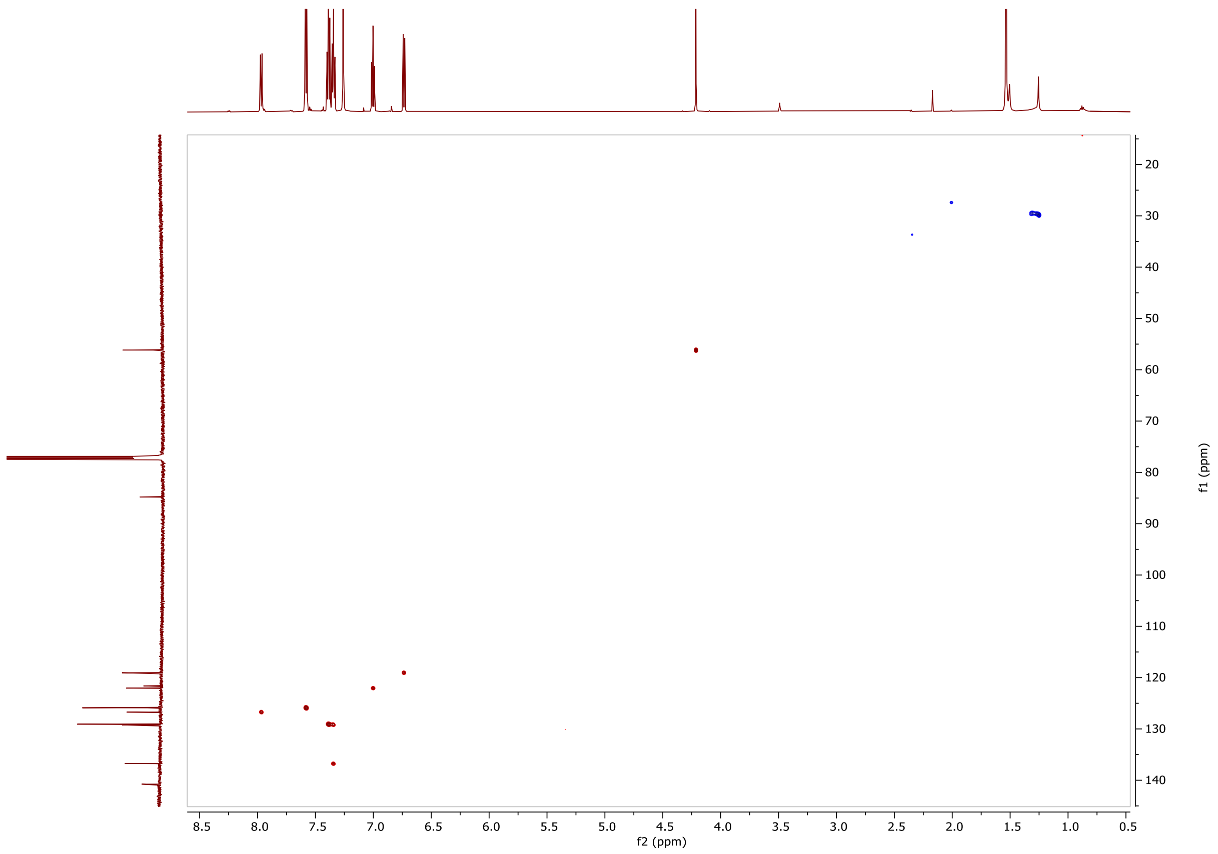

Figure S4. HSQC spectrum (600.19/150.91 MHz, $CHCl_3$-*d*) of compound 1.

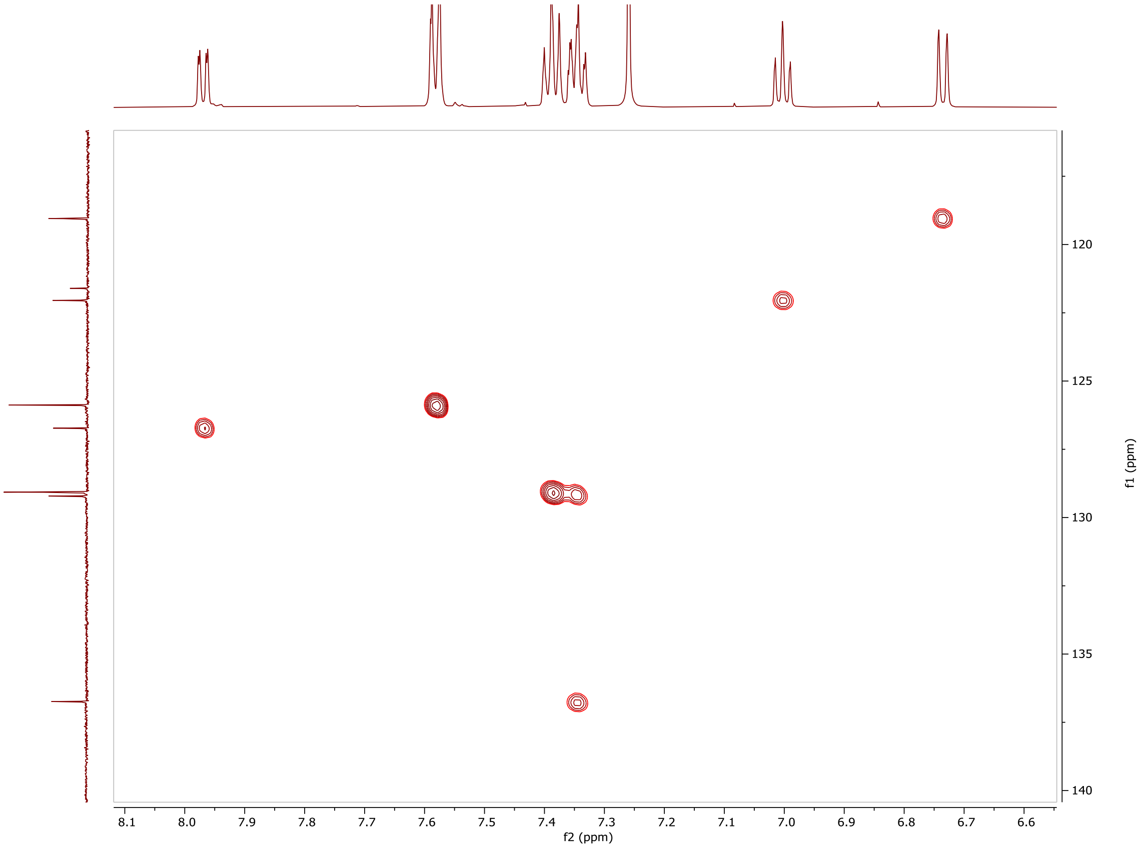

Figure S5. HSQC spectrum (600.19/150.91 MHz, $CHCl_3$-*d*) of compound 1 (insert *δ* 8.0 – 6.5 ppm).

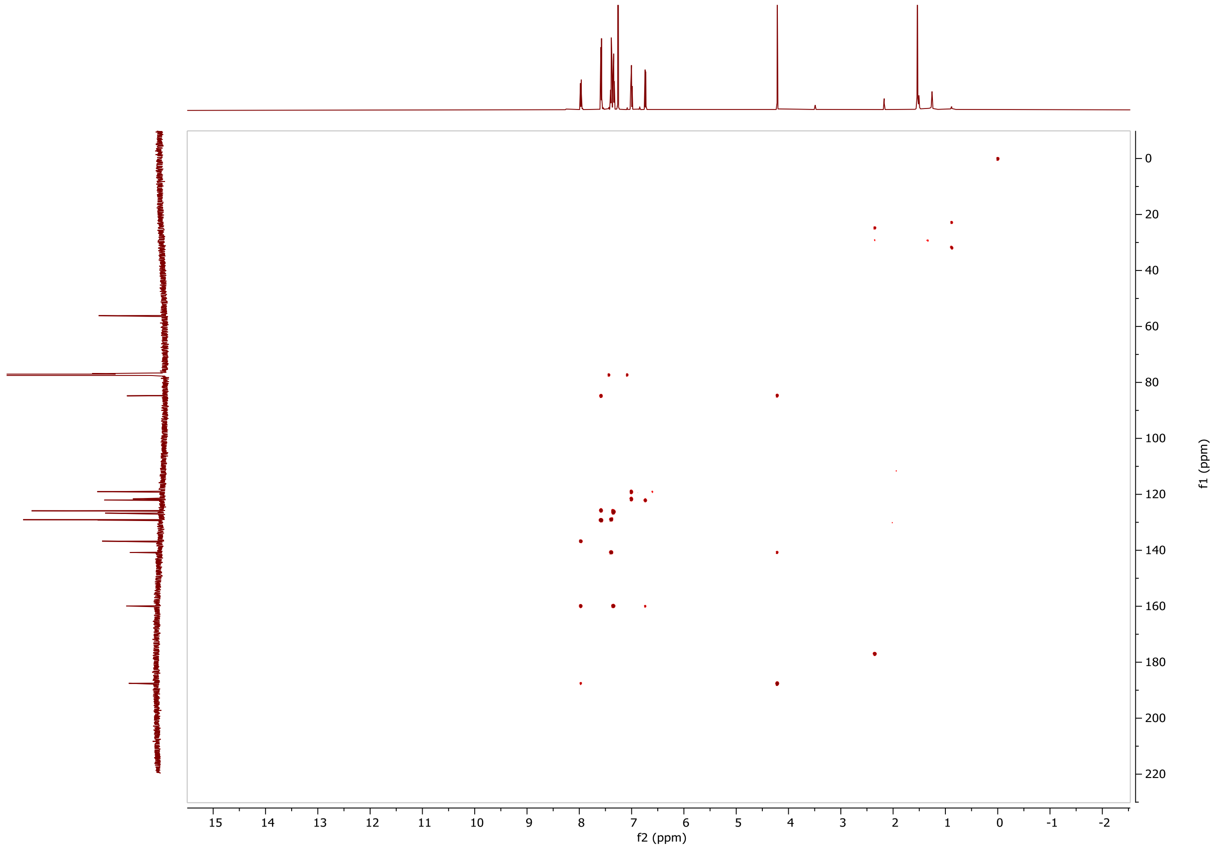

Figure S6. HMBC spectrum (600.19/150.91 MHz, $CHCl_3$-*d*) of compound 1.

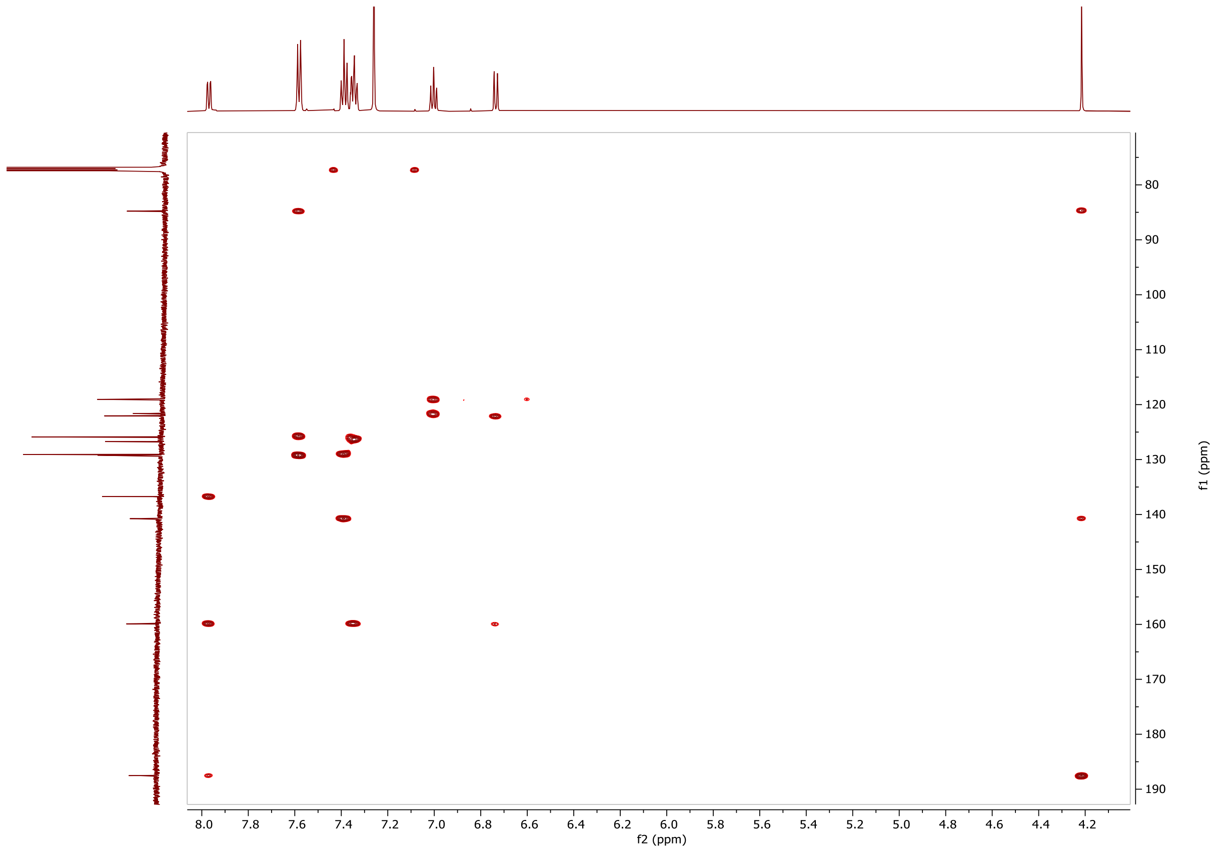

Figure S7. HSQC spectrum (600.19/150.91 MHz, $CHCl_3$-*d*) of compound 1 (insert δ 8.0 – 4.0 ppm).

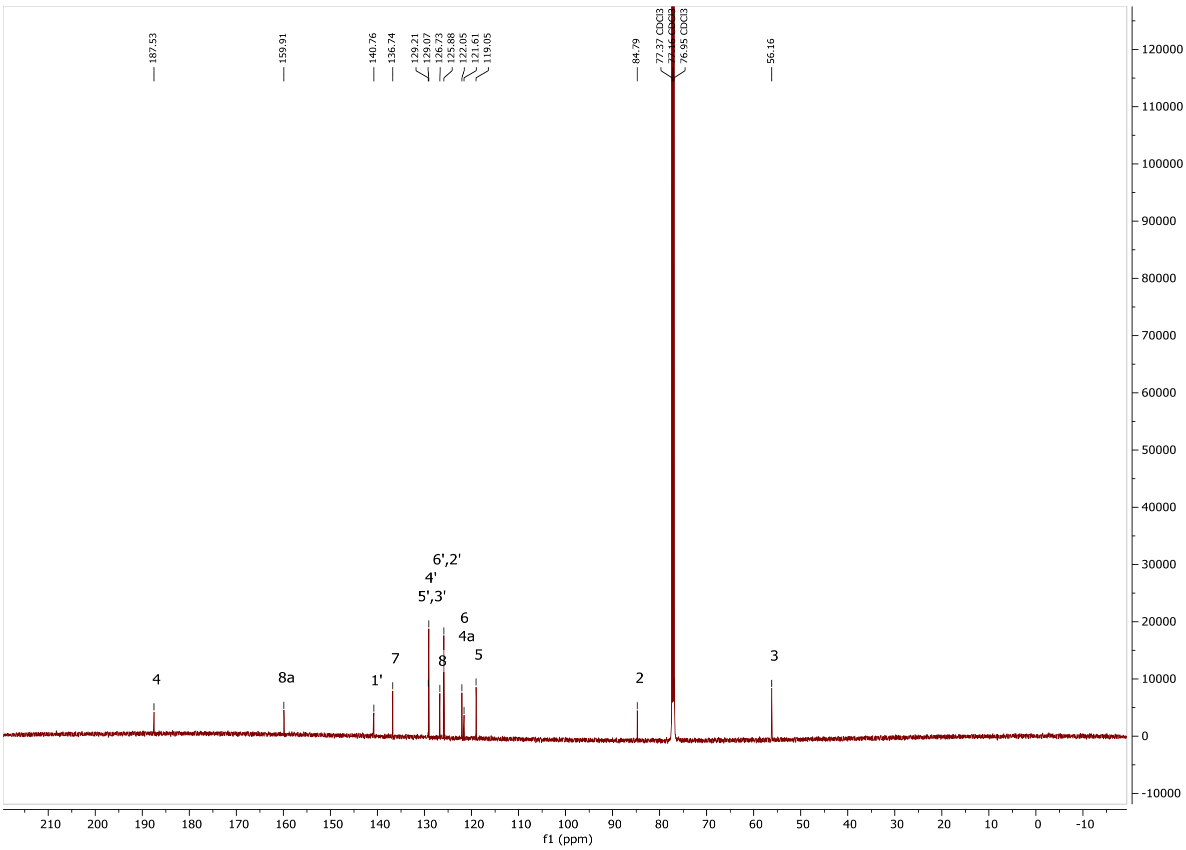

Figure S8. ^{13}C-NMR spectrum (150.91 MHz, CHCl3-*d*) of compound **1**.

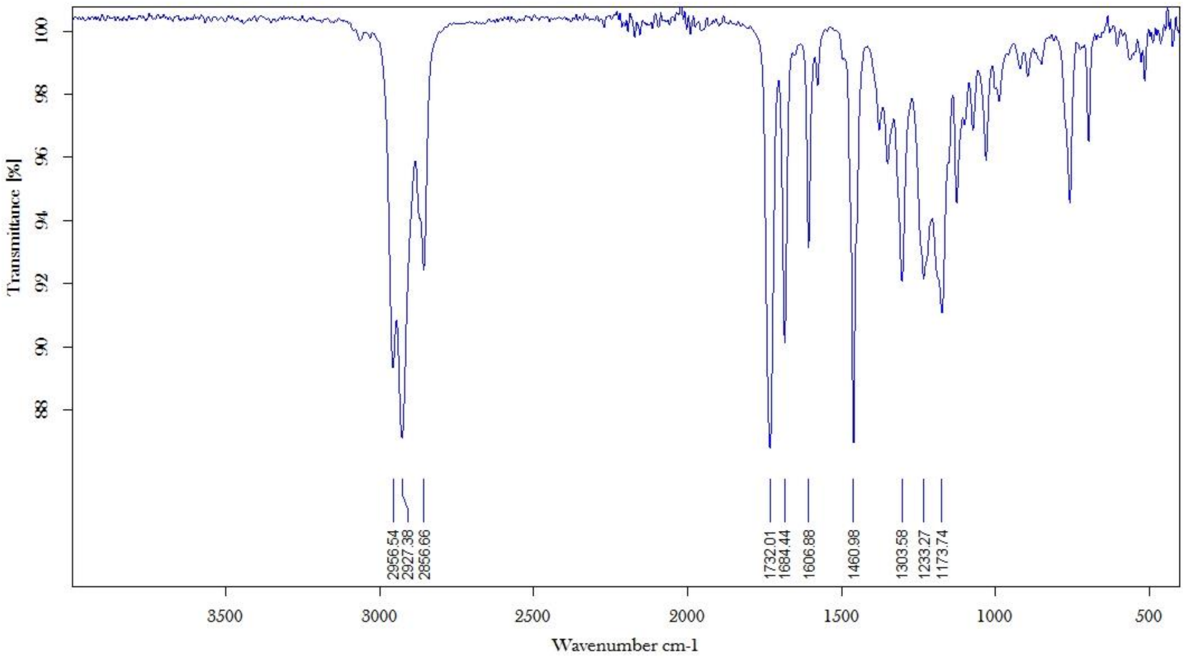

Figure S9. IR spectrum of compound 1.

Mass Spectrum SmartFormula Report

Analysis Info

Analysis Name	D:\Data\Pharmakognosie\Mostafa\pure_compounds_LCMS\Drev_48-51_F7_prep-2-pos_62_01_26122.d	Acquisition Date	2/7/2020 10:20:03 PM
Method	screen_neg_2019.m	Operator	Simon
Sample Name	Drev_48-51_F7_prep-2-pos	Instrument / Ser#	micrOTOF-Q 10202
Comment	#7 - oeliger A. 1200784/0<br>Aceton		

Acquisition Parameter

Source Type	ESI	Ion Polarity	Negative	Set Nebulizer	1.6 Bar
Focus	Not active	Set Capillary	3500 V	Set Dry Heater	220 °C
Scan Begin	100 m/z	Set End Plate Offset	-500 V	Set Dry Gas	6.0 l/min
Scan End	1500 m/z	Set Collision Cell RF	452.4 Vpp	Set Divert Valve	Source

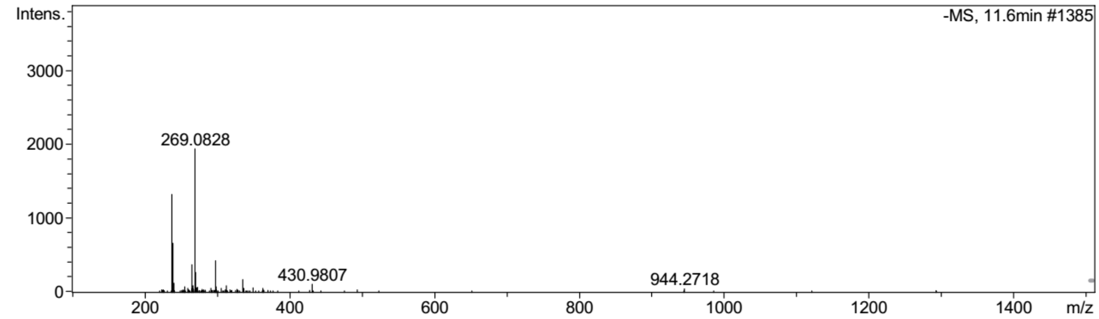

Meas. m/z	#	Formula	Score	m/z	err [mDa]	err [ppm]	mSigma	rdb	e⁻ Conf	N-Rule
237.0577	1	C 15 H 9 O 3	100.00	237.0557	-2.0	-8.5	277.2	11.5	even	ok

Figure S10. HRLCESIMS spectrum of compound 1.

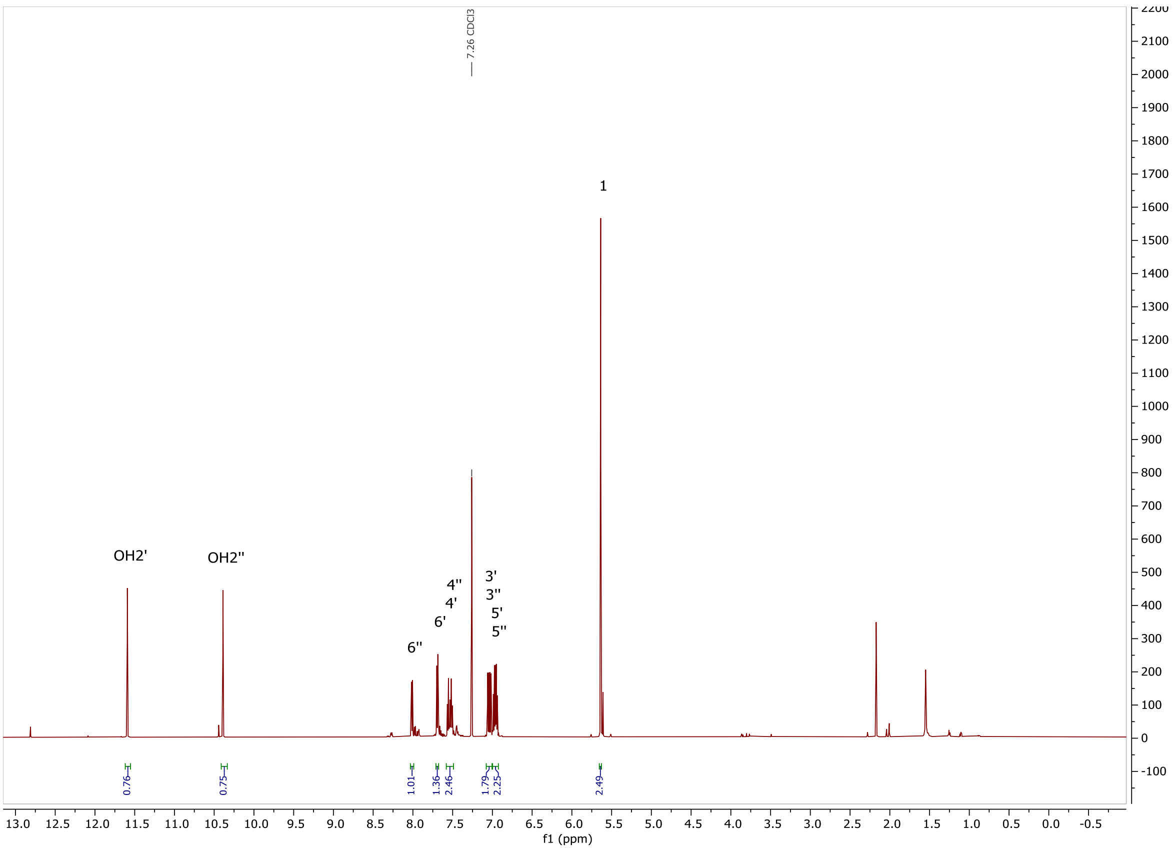

Figure S11. ^{1}**H-NMR (600.19 MHz, CHCl₃-*d*) of compound 2.**

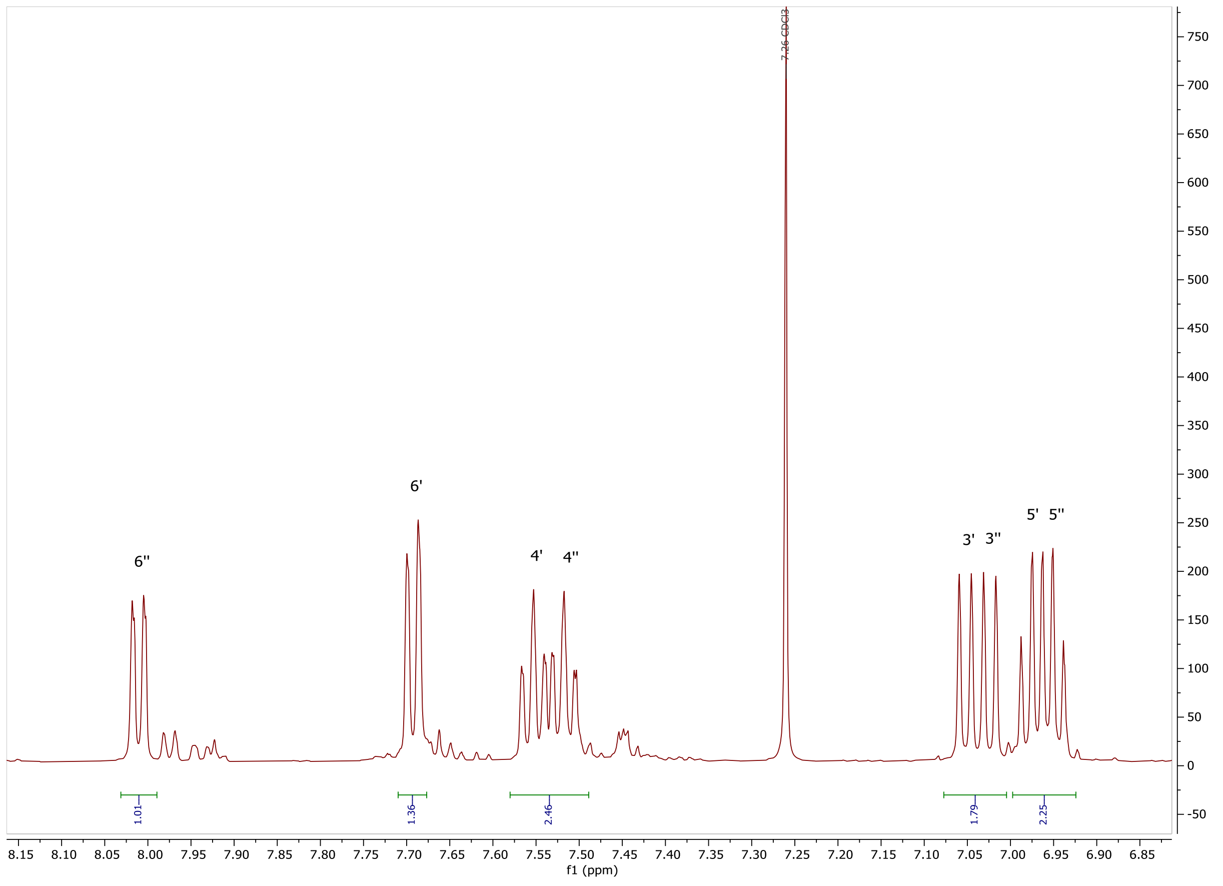

Figure S12. ¹H-NMR (600.19 MHz, $CHCl_3$-*d*) of compound 2 (insert δ 8.2 – 6.7 ppm).

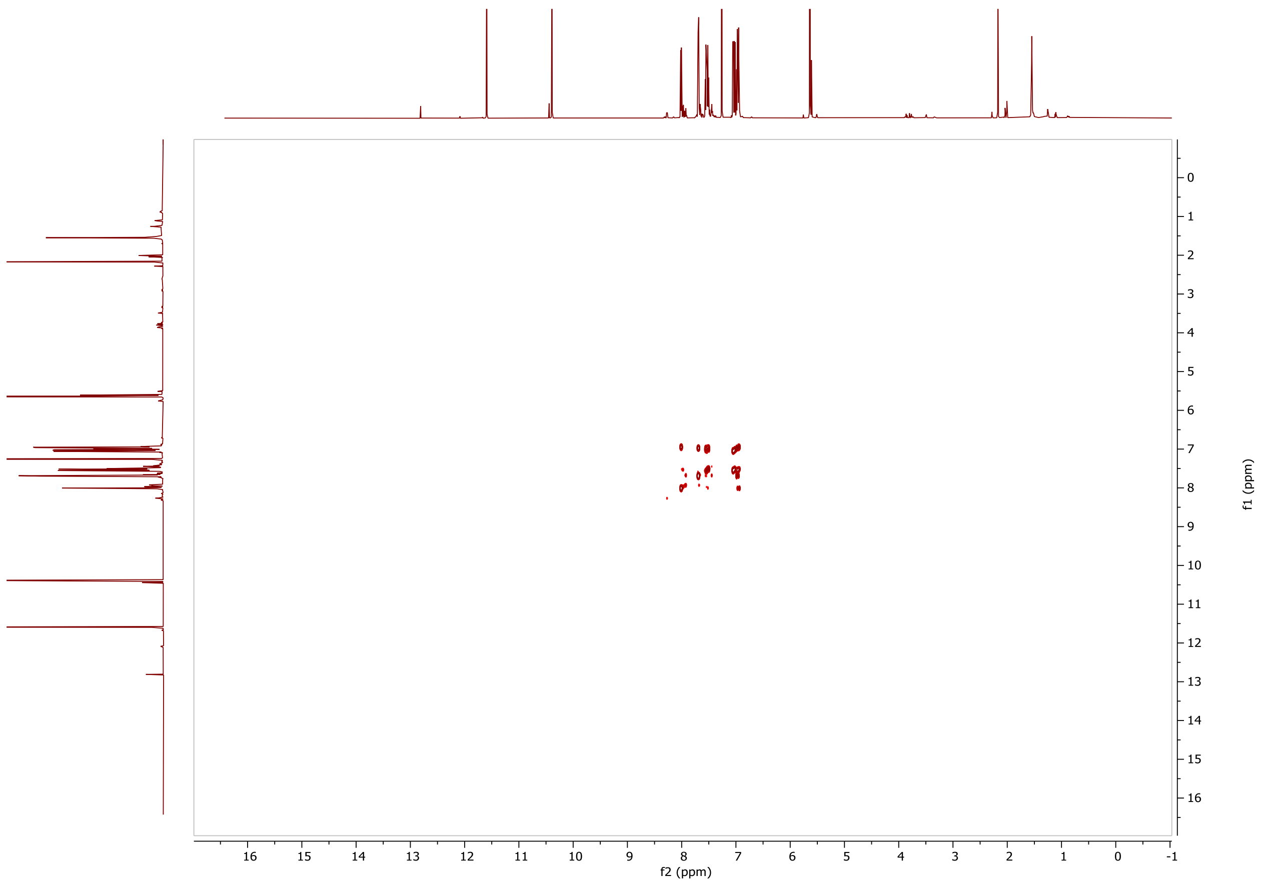

Figure S13. COSY spectrum (600.19 MHz, $CHCl_3$-*d*) of compound 2.

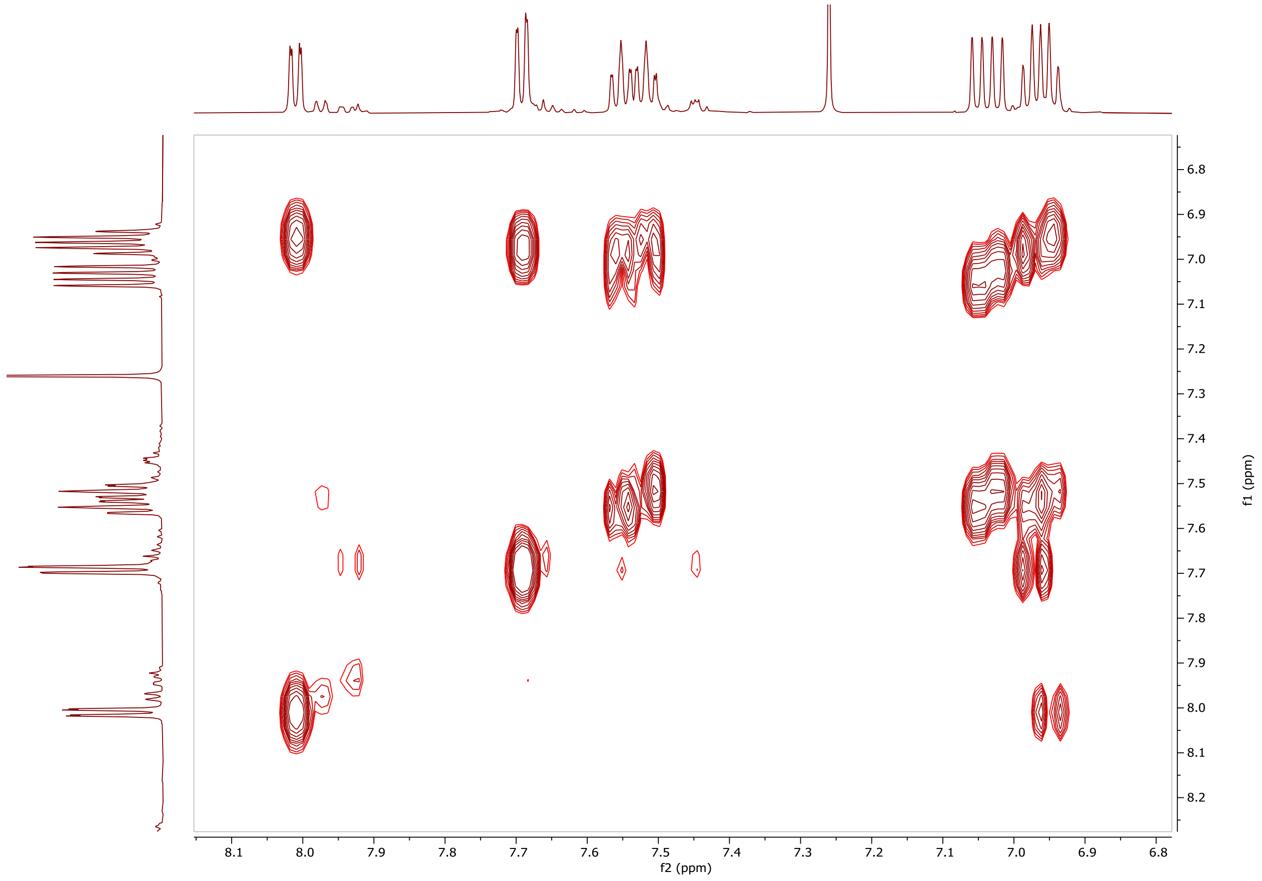

Figure S14. COSY spectrum (600.19 MHz, CHCl3-*d*) of compound 2 (insert δ 8.2 – 6.7 ppm).

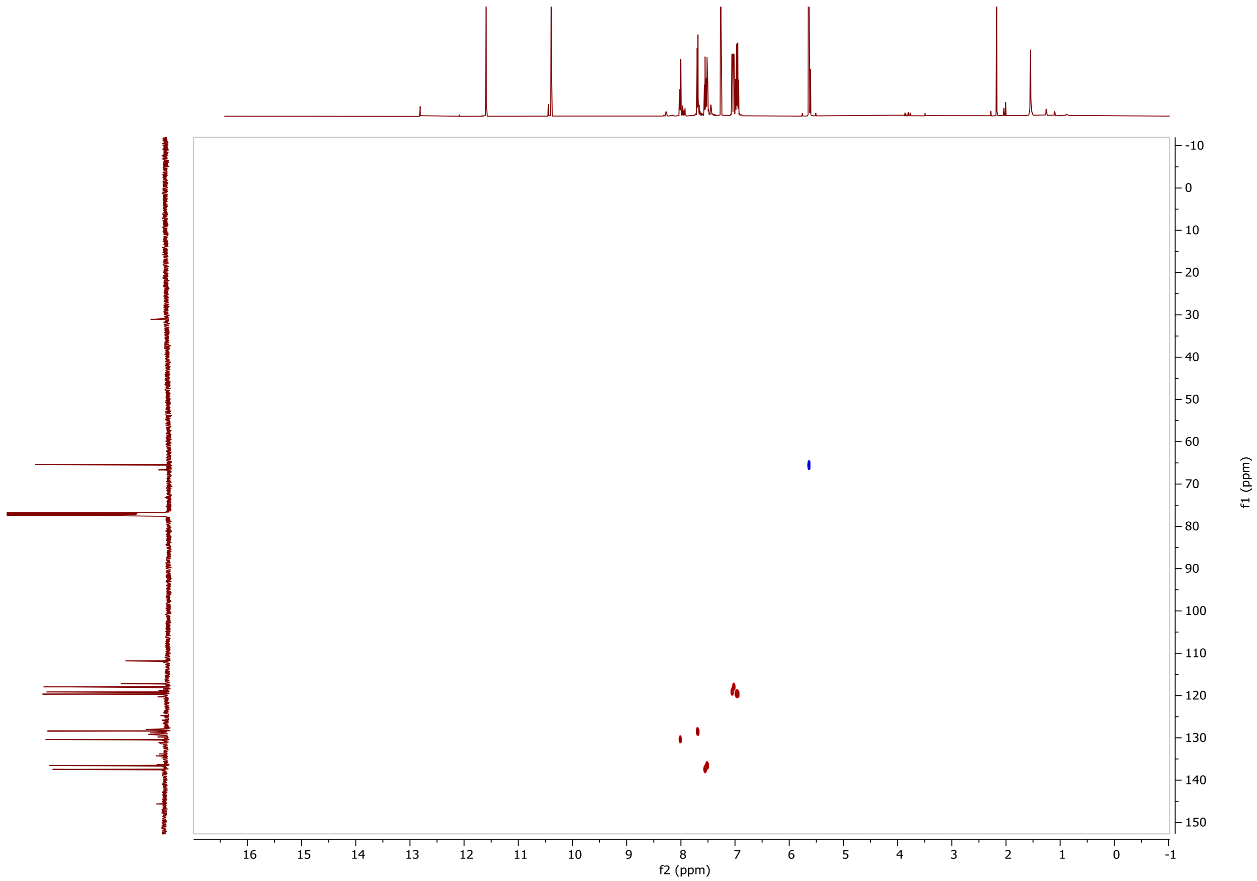

Figure S15. HSQC spectrum (600.19/150.91 MHz, $CHCl_3$-*d*) of compound 2.

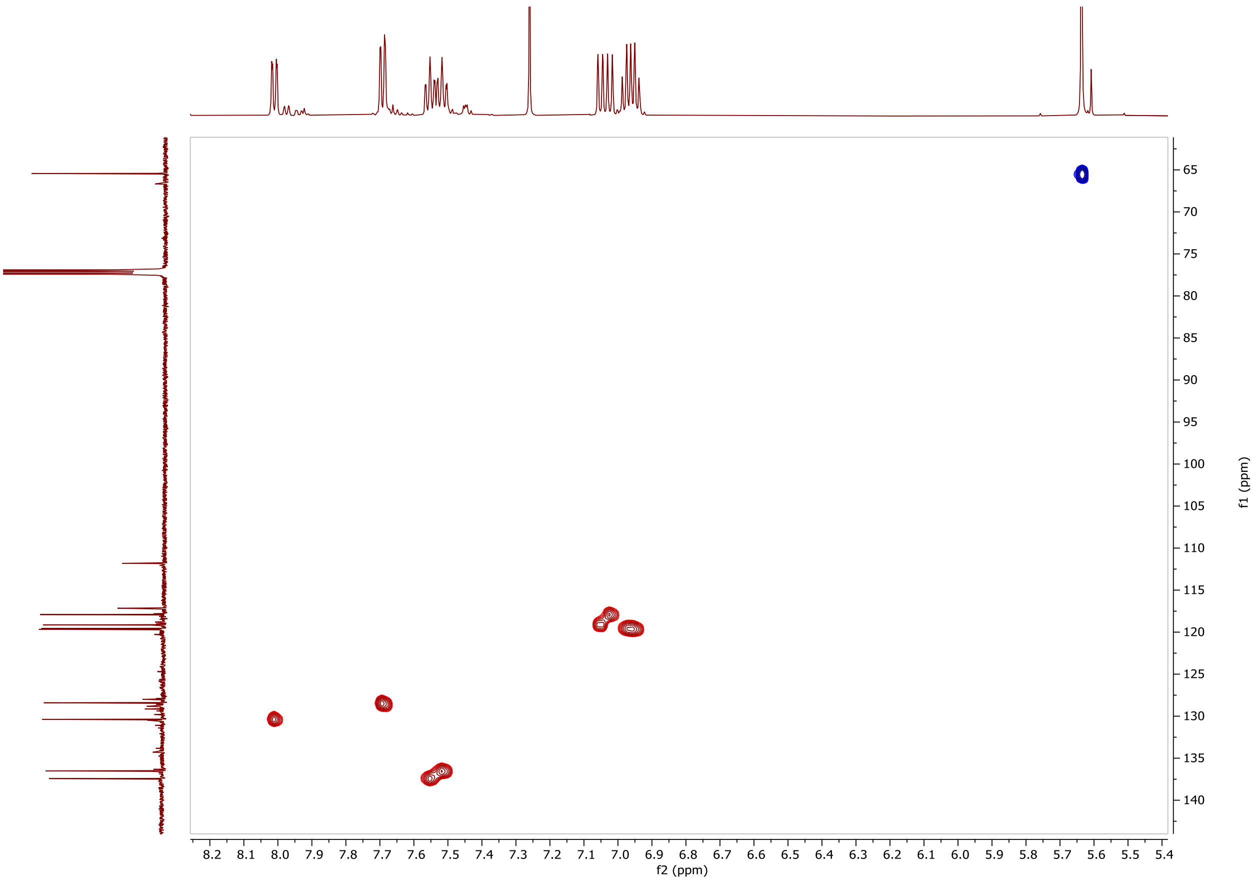

Figure S16. HSQC spectrum (600.19/150.91 MHz, $CHCl_3$-*d*) of compound 2 (insert δ 8.2 – 5.4 ppm).

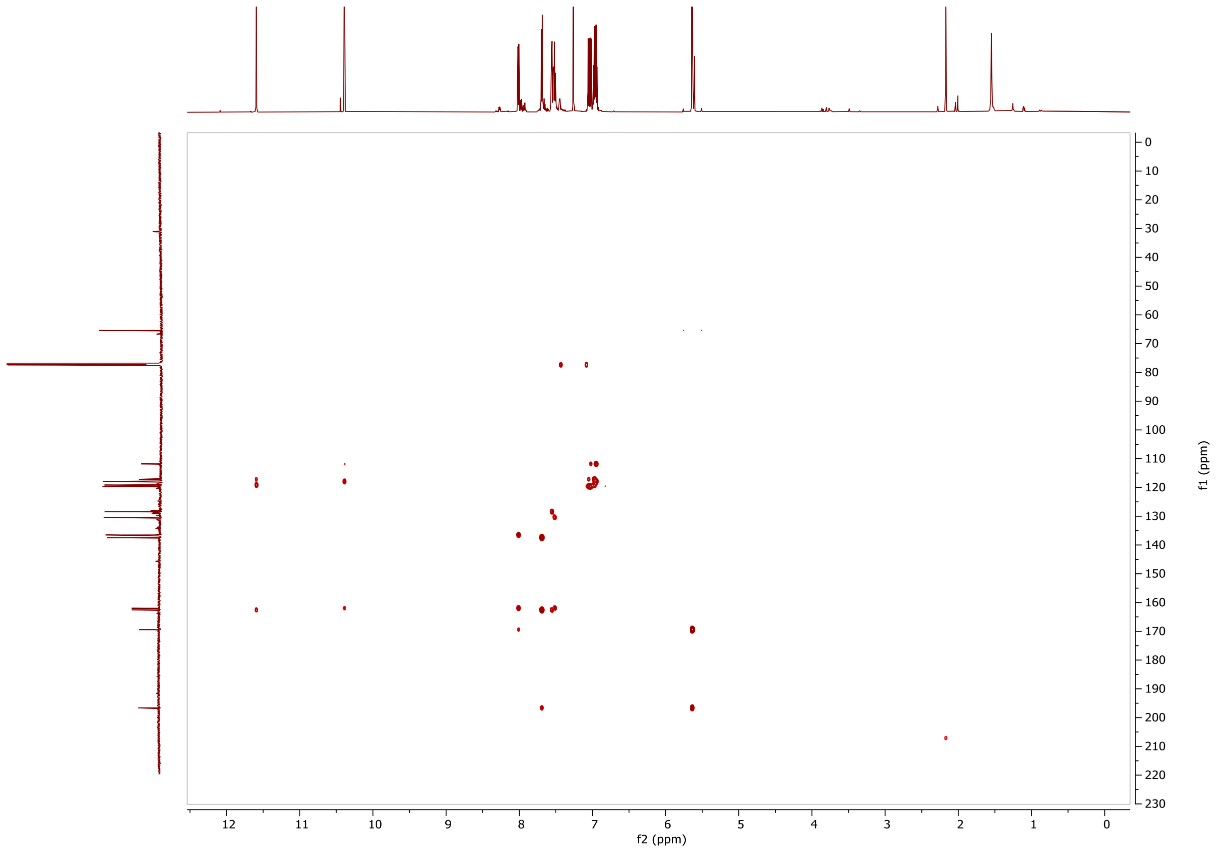

Figure S17. HMBC spectrum (600.19/150.91 MHz, $CHCl_3$-*d*) of compound 2.

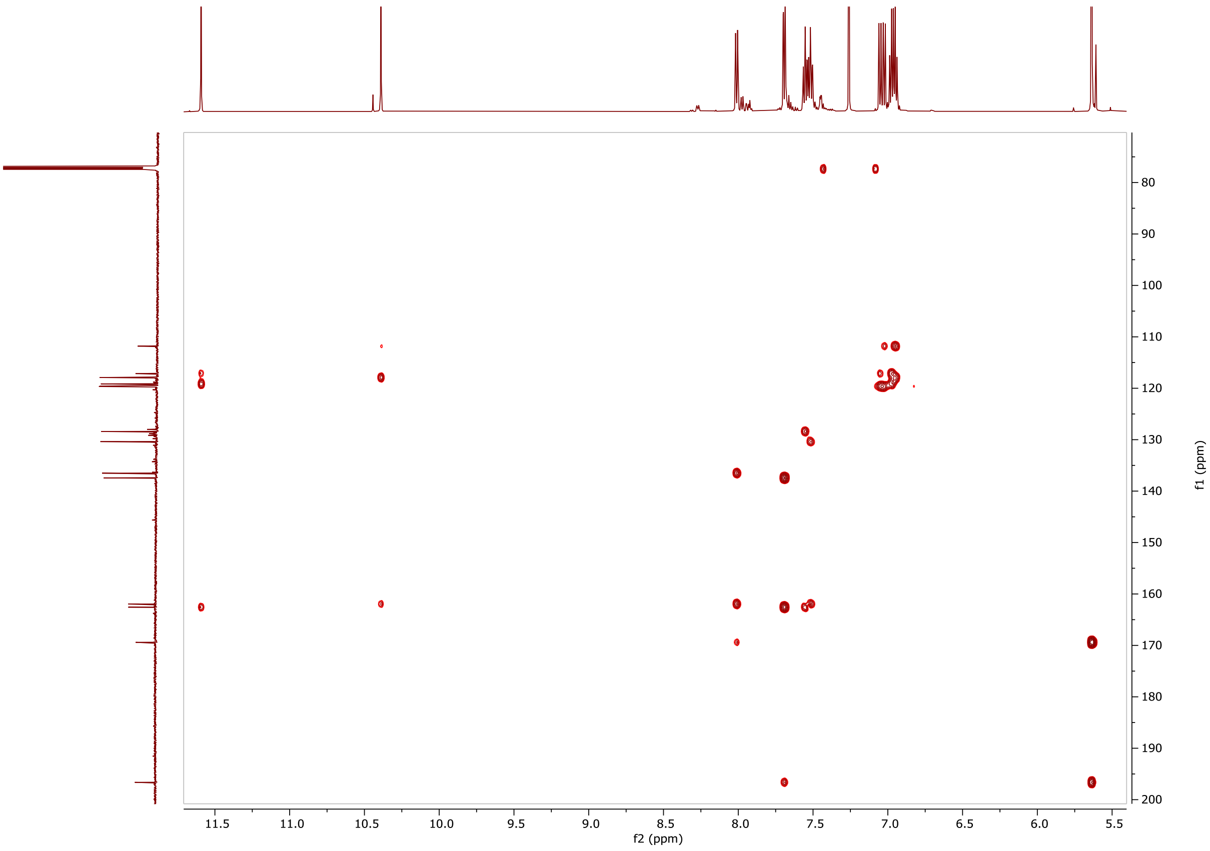

Figure S18. HMBC spectrum (600.19/150.91 MHz, CHCl$_3$-*d*) of compound 22 (insert δ 11.7 – 5.5 ppm).

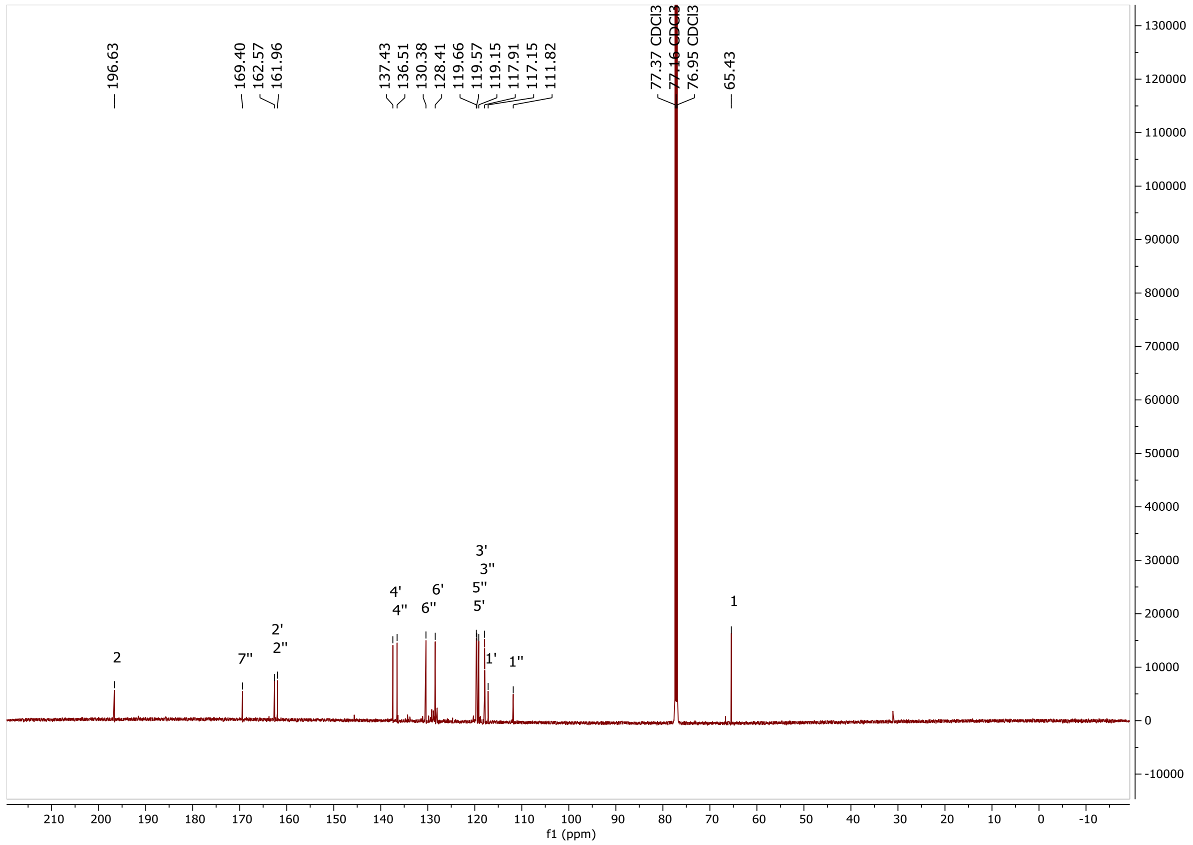

Figure S19. **^{13}C-NMR (600.19/150.91 MHz, CHCl3-*d*) of compound 2.**

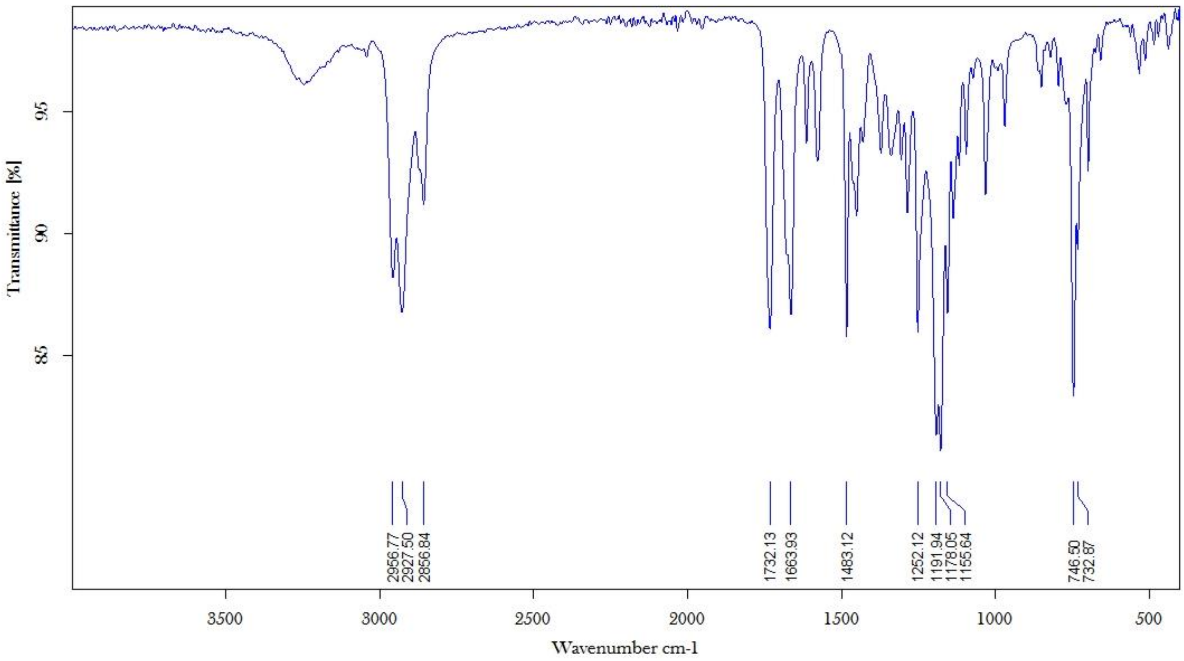

Figure S20. IR spectrum of compound 2.

Mass Spectrum SmartFormula Report

Analysis Info

Analysis Name	D:\Data\Pharmakognosie\Mostafa\pure_compounds_LCMS\F8PS9_THF_pos_45_01_26091.d	Acquisition Date	1/10/2020 8:39:13 PM
Method	screen_pos_2019.m	Operator	Simon
Sample Name	F8PS9_THF_pos	Instrument / Ser#	micrOTOF-Q 10202
Comment	#7 - oeliger A. 1200784/0 Aceton		

Acquisition Parameter

Source Type	ESI	Ion Polarity	Positive	Set Nebulizer	1.6 Bar
Focus	Not active	Set Capillary	4500 V	Set Dry Heater	220 °C
Scan Begin	100 m/z	Set End Plate Offset	-500 V	Set Dry Gas	6.0 l/min
Scan End	1500 m/z	Set Collision Cell RF	452.4 Vpp	Set Divert Valve	Source

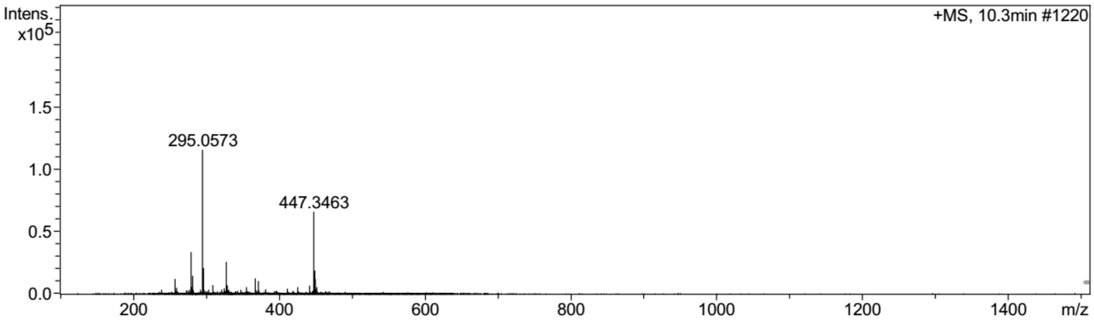

Meas. m/z	#	Formula	Score	m/z	err [mDa]	err [ppm]	mSigma	rdb	e⁻ Conf	N-Rule
295.0573	1	C 15 H 12 Na O 5	100.00	295.0577	0.4	1.3	8.3	9.5	even	ok
	2	C 13 H 13 Na 2 O 5	28.64	295.0553	-2.0	-6.9	20.9	6.5	even	ok

Figure S21. HRLCESIMS spectrum of compound 2.

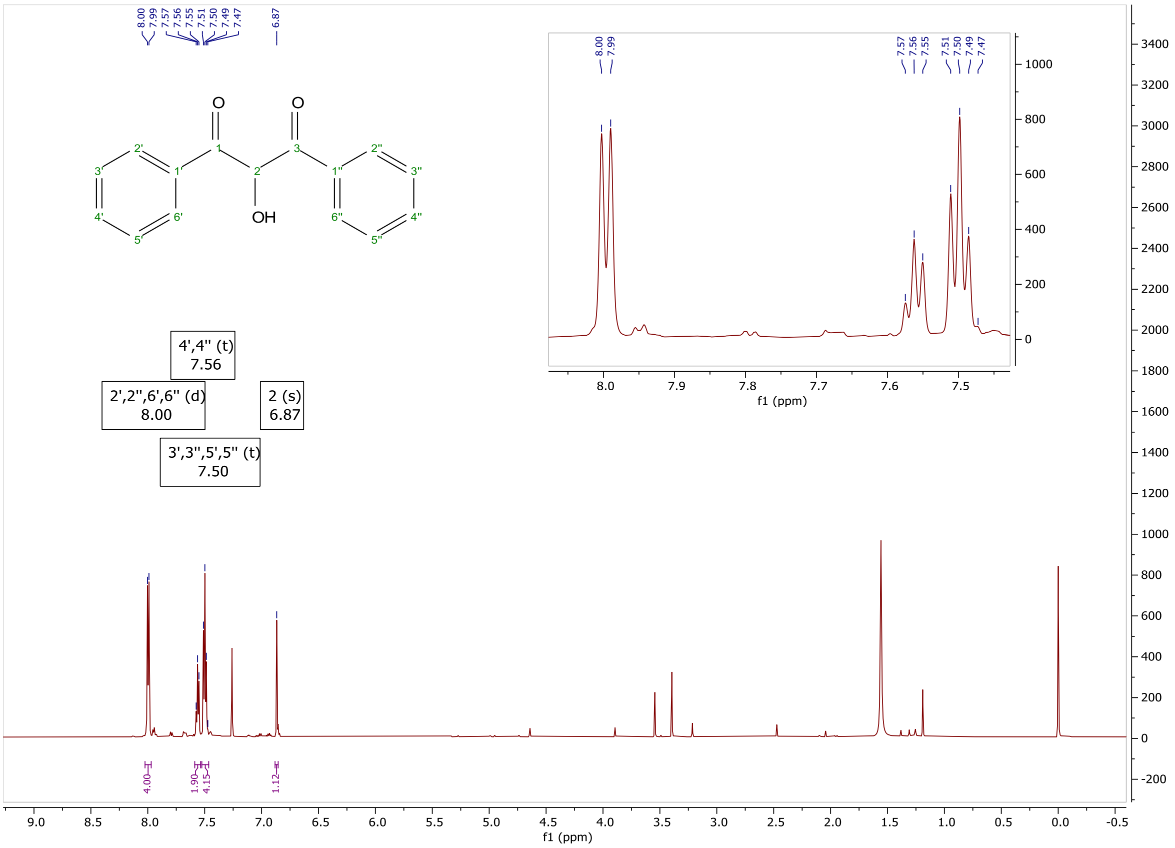

Figure S22. **¹H-NMR (600.19 MHz, CHCl₃-*d*) of compound 3.**

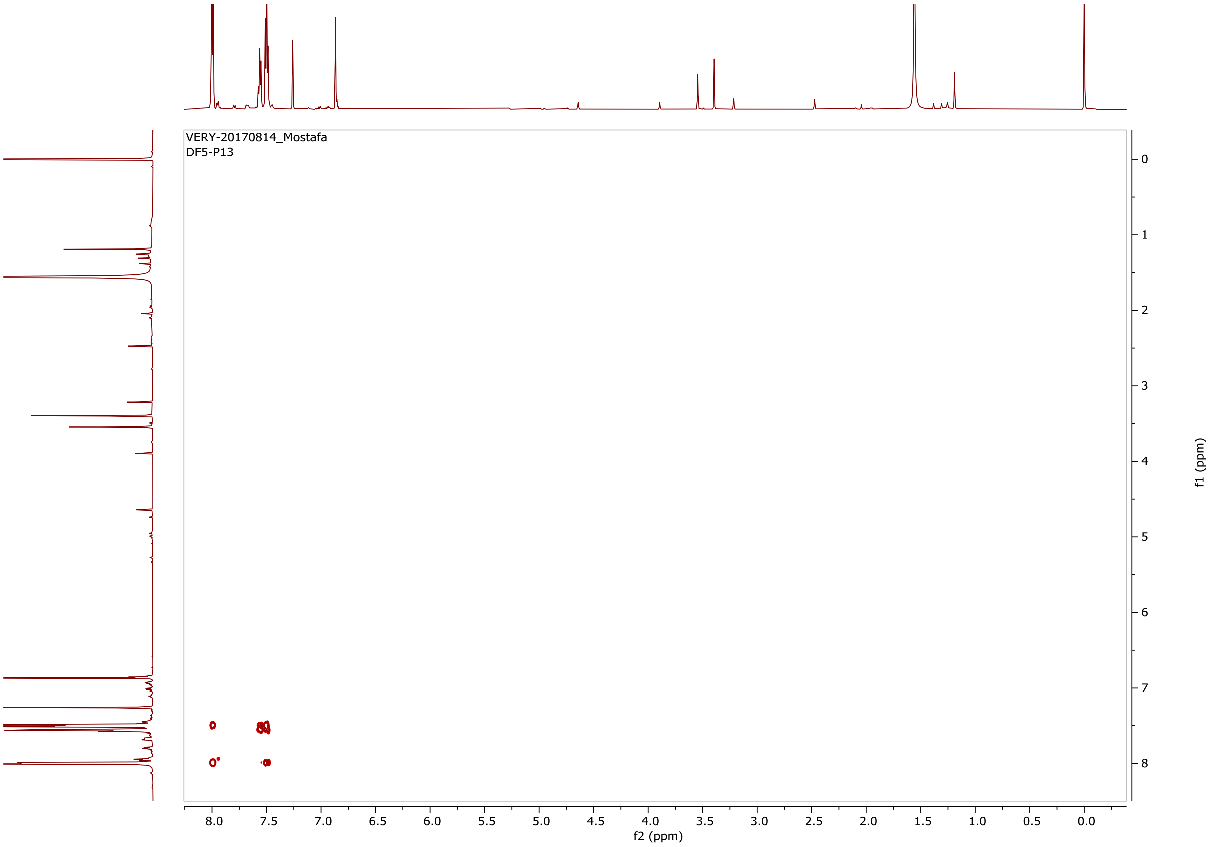

Figure S23. COSY spectrum (600.19 MHz, $CHCl_3$-*d*) of compound 3.

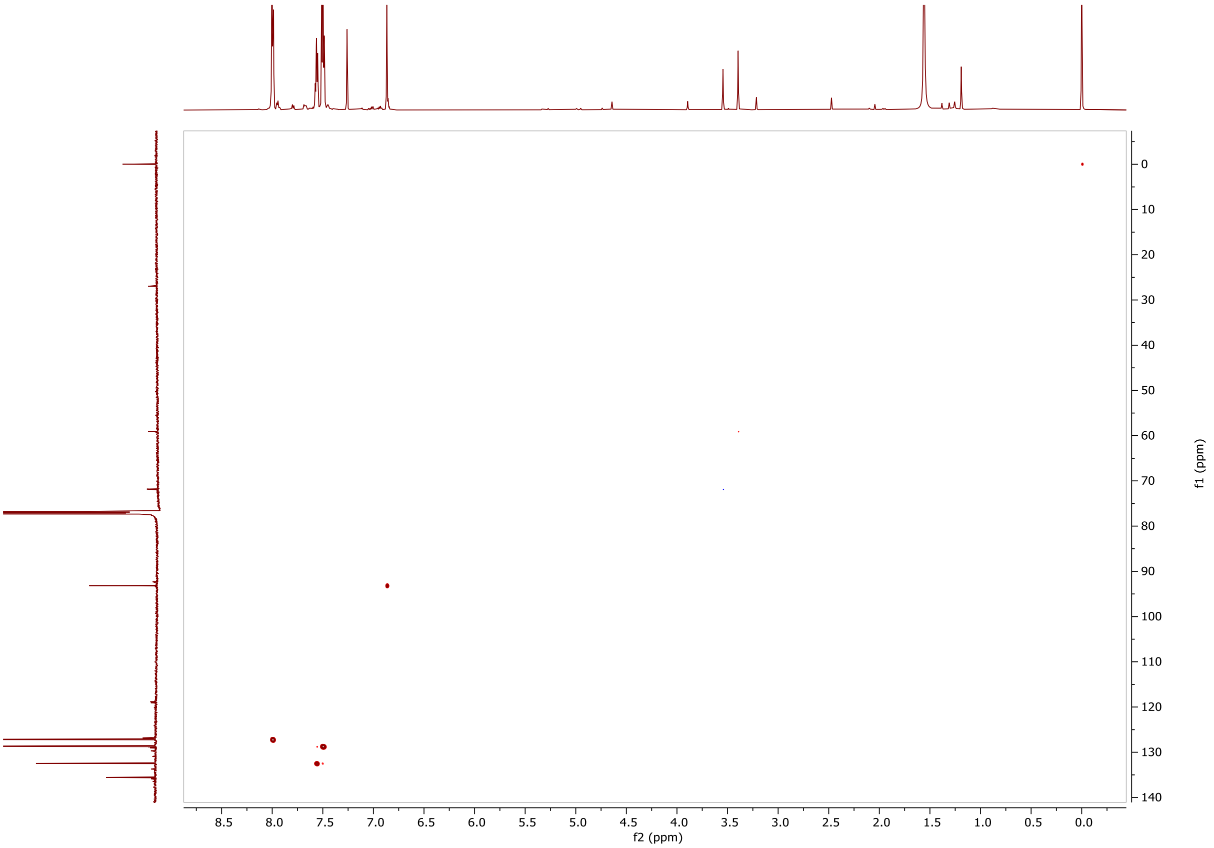

Figure S24. HSQC spectrum (600.19, 150.91 MHz, $CHCl_3$-*d*) of compound 3.

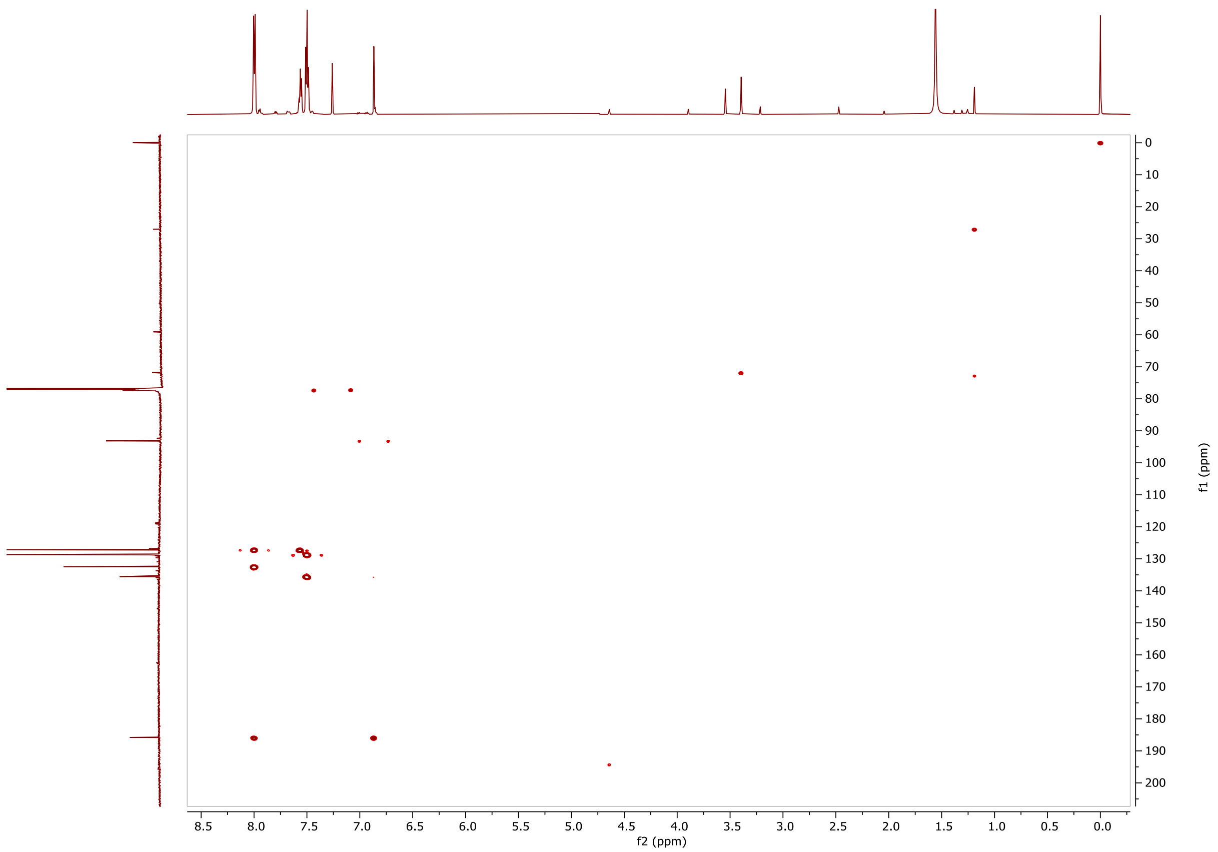

Figure S25. HMBC spectrum (600.19, 150.91 MHz, $CHCl_3$-*d*) of compound 3.

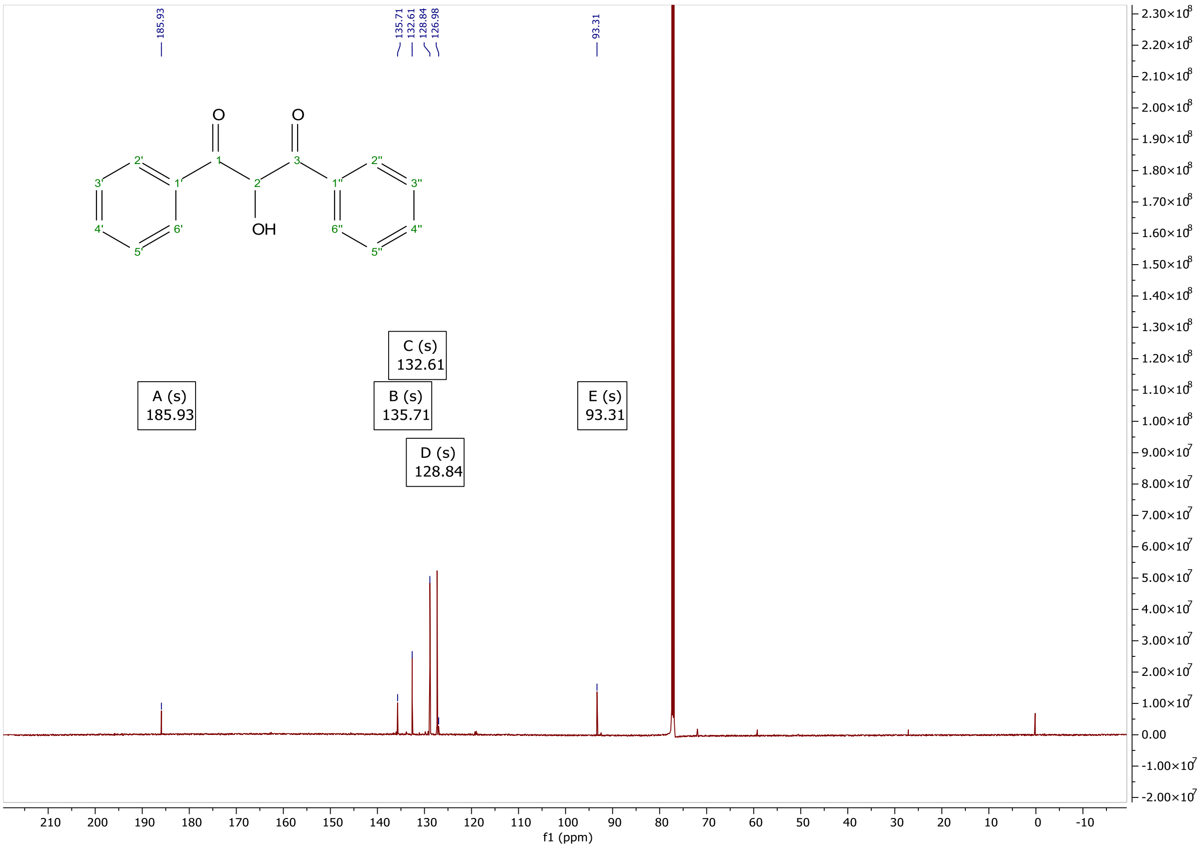

Figure S26. **^{13}C-NMR (150.91 MHz, $CHCl_3$-*d*) of compound 3.**

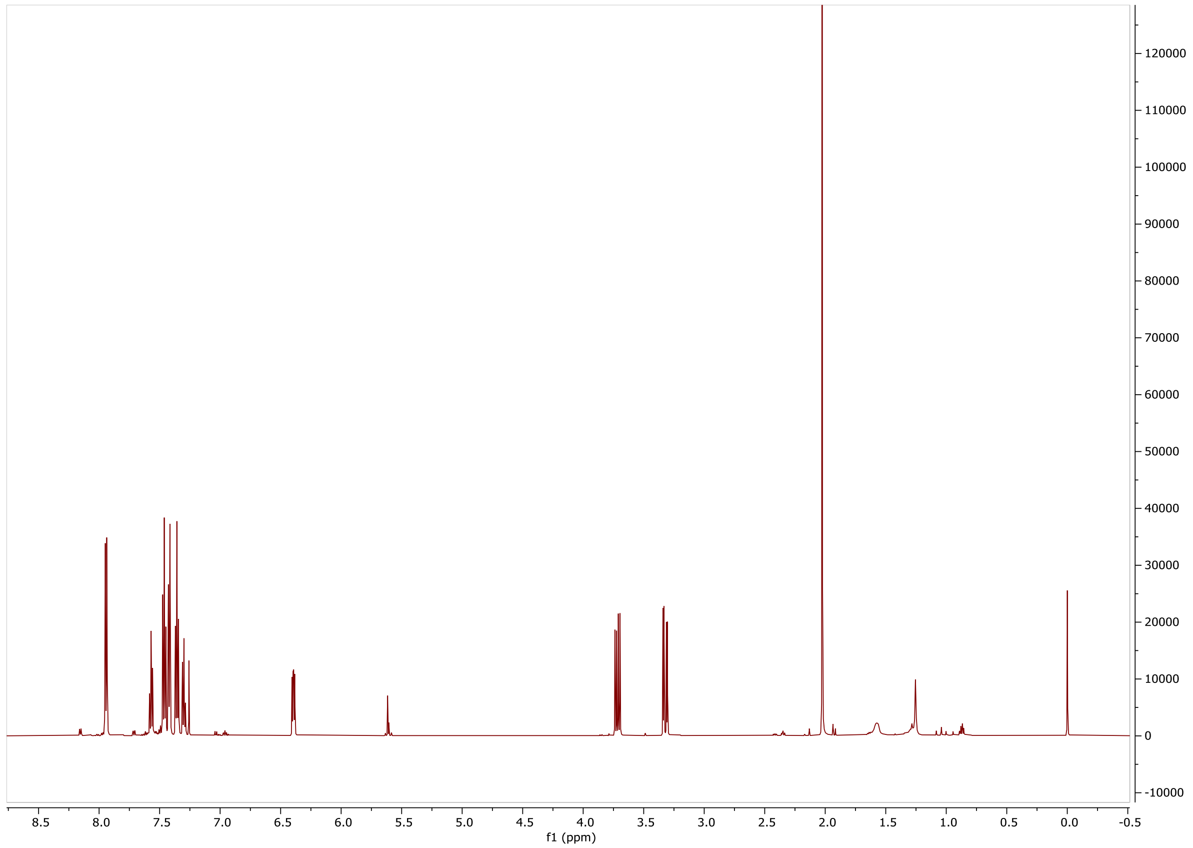

Figure S27. ^{1}H-NMR (600.19 MHz, $CHCl_3$-*d*) of compound 4.

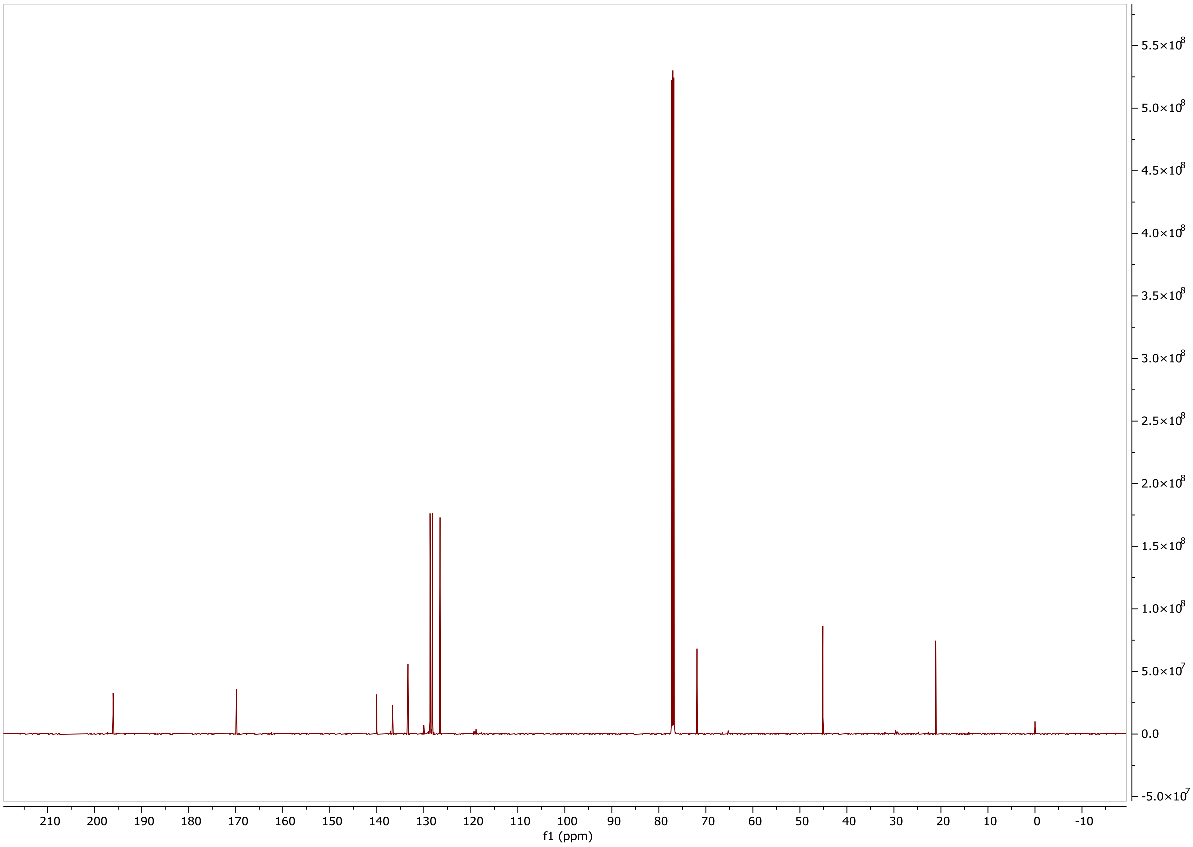

Figure S28. ^{13}**C-NMR (150.91 MHz, CHCl$_3$-*d*) of compound 4.**

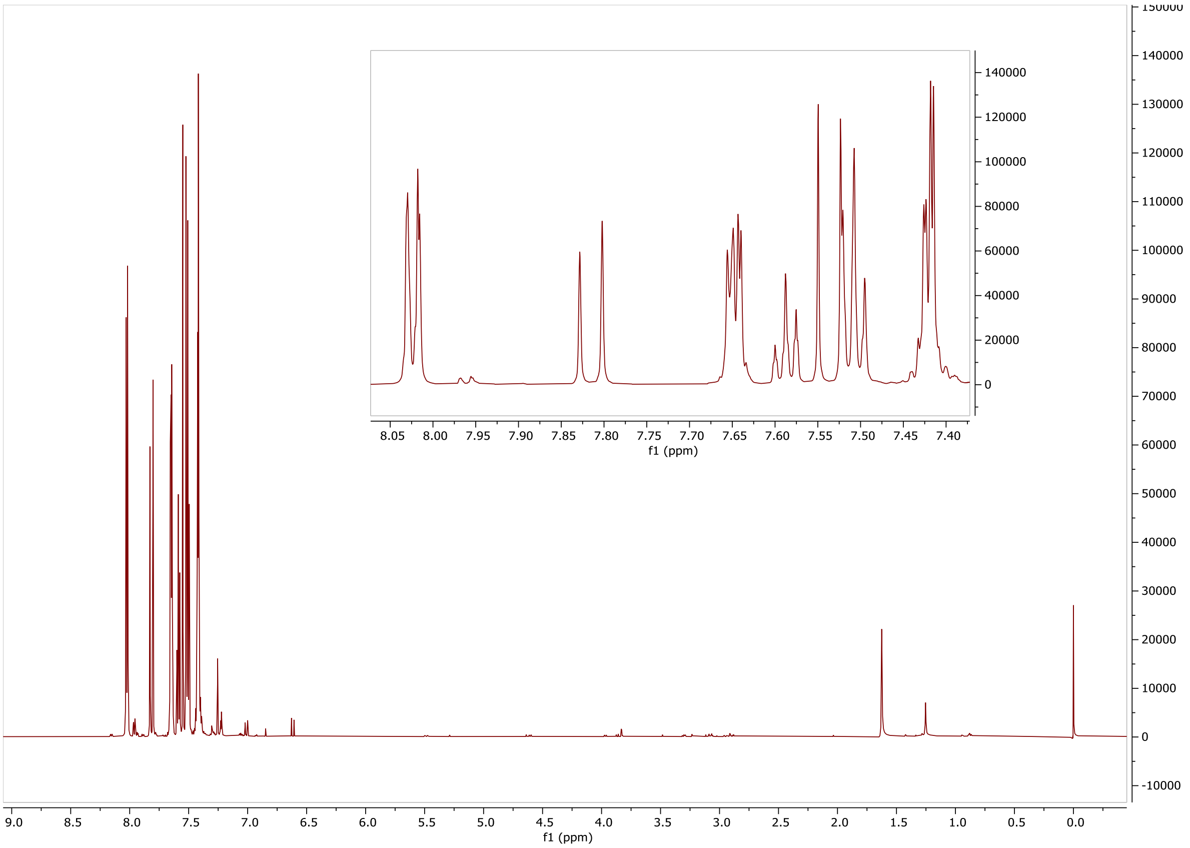

Figure S29. ¹H-NMR (600.19 MHz, CHCl₃-*d*) of compound 5.

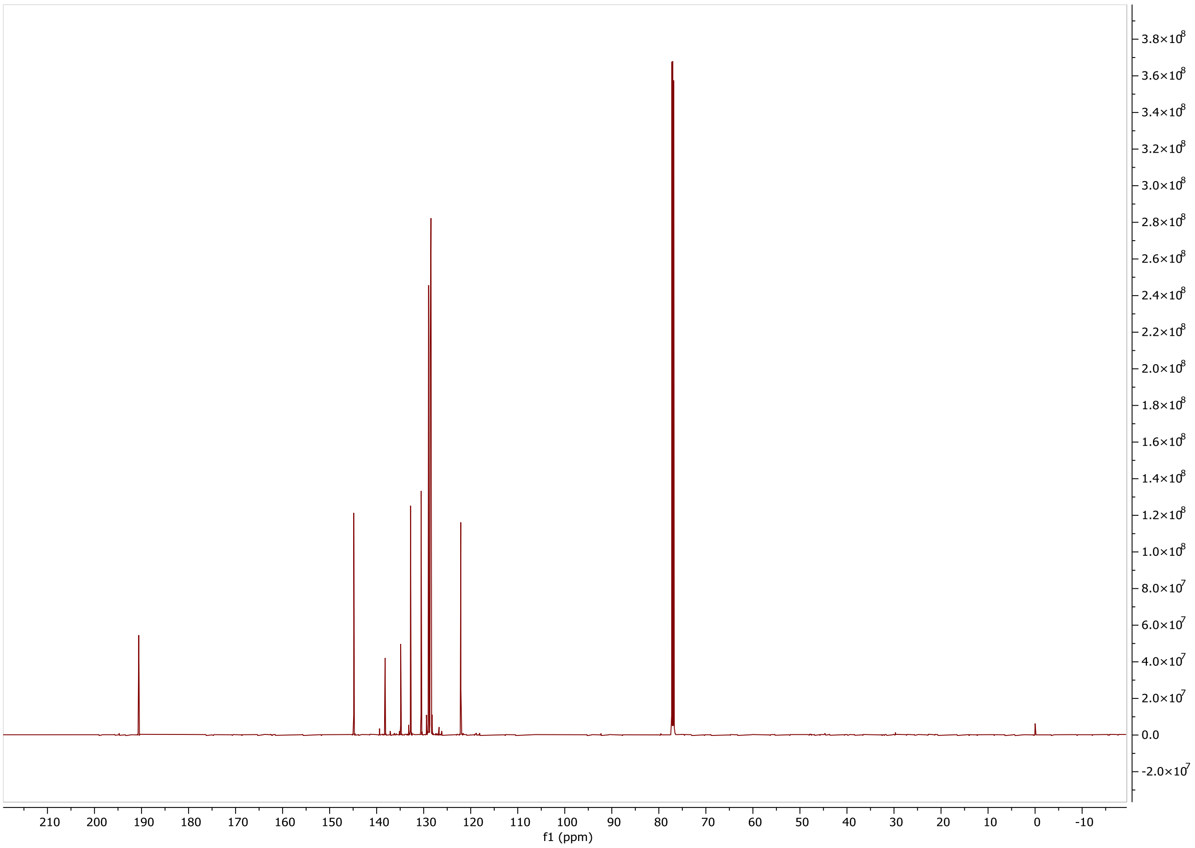

Figure S30. ^{13}C-NMR (150.91 MHz, $CHCl_3$-*d*) of compound 5.

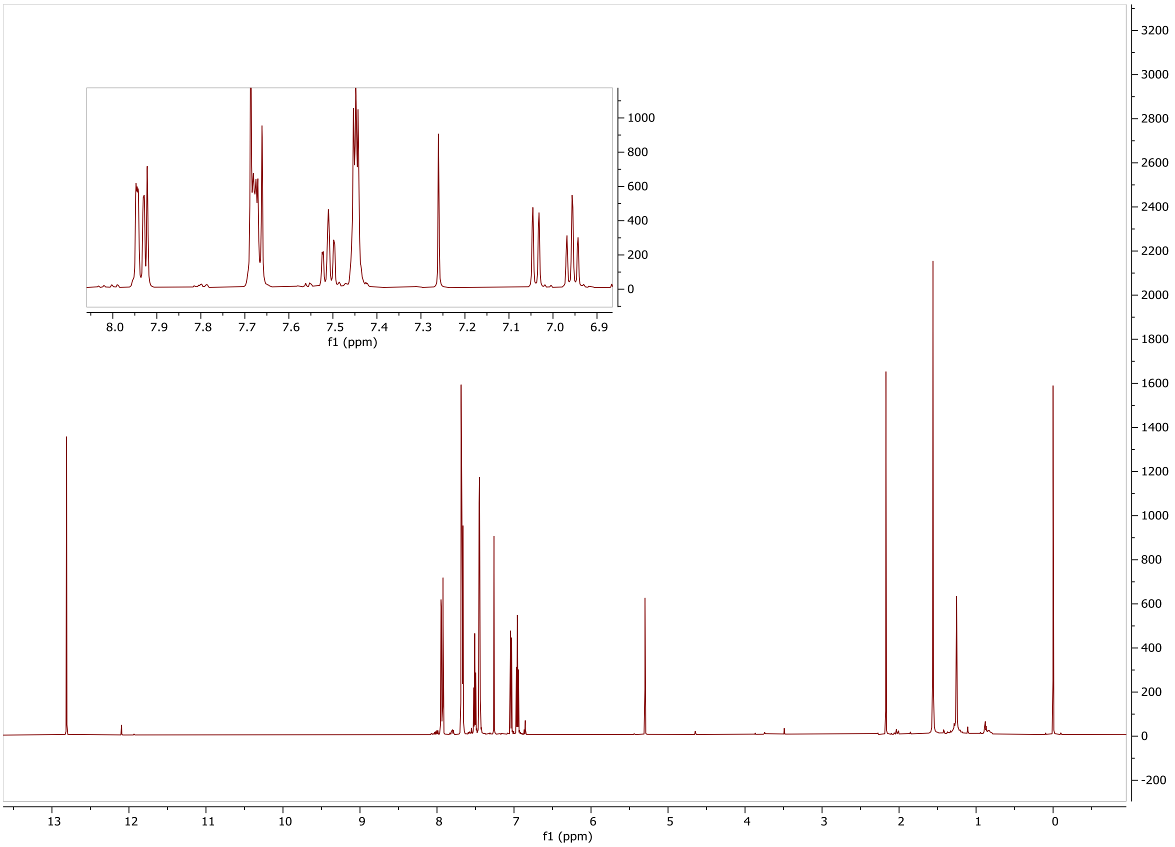

Figure S31. ¹H-NMR (600.19 MHz, $CHCl_3$-*d*) of compound 6.

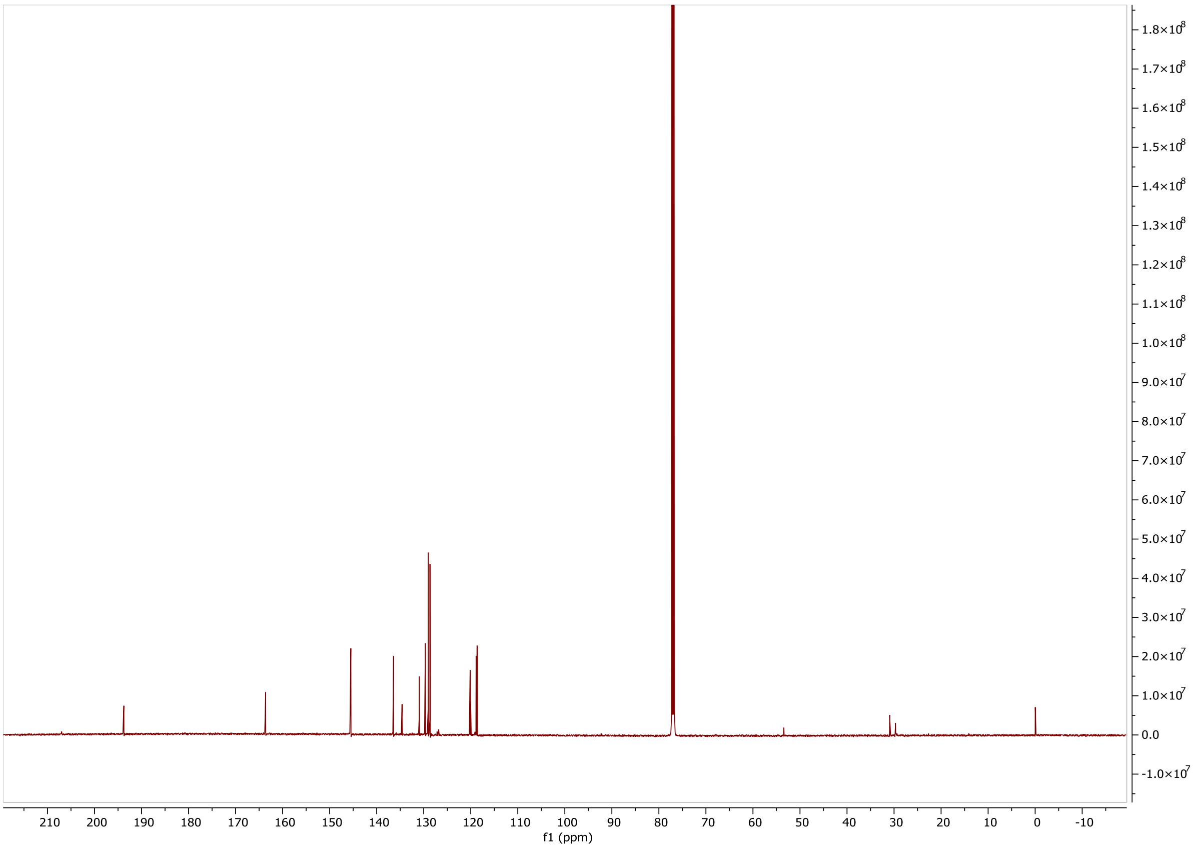

Figure S32. **^{13}C-NMR (150.91 MHz, $CHCl_3$-*d*) of compound 6.**

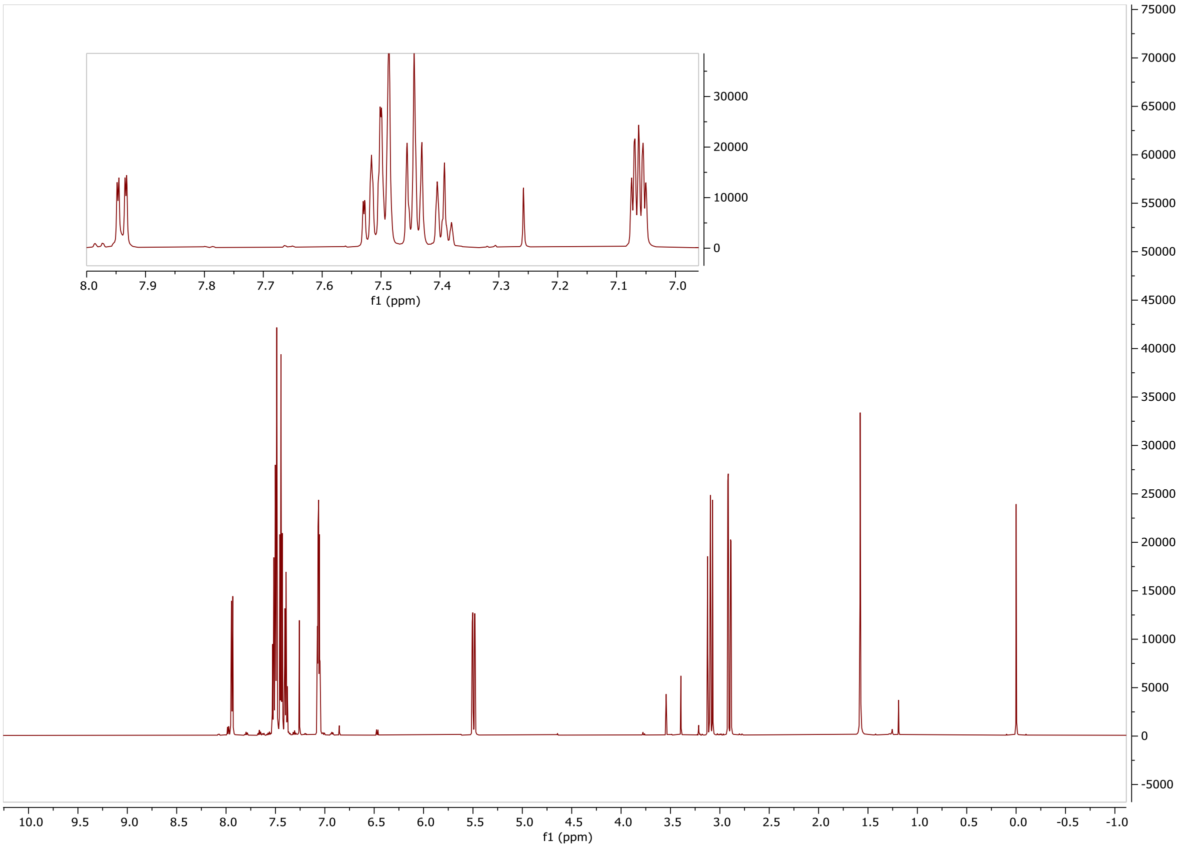

Figure S33. **¹H-NMR (600.19 MHz, $CHCl_3$-*d*) of compound 7.**

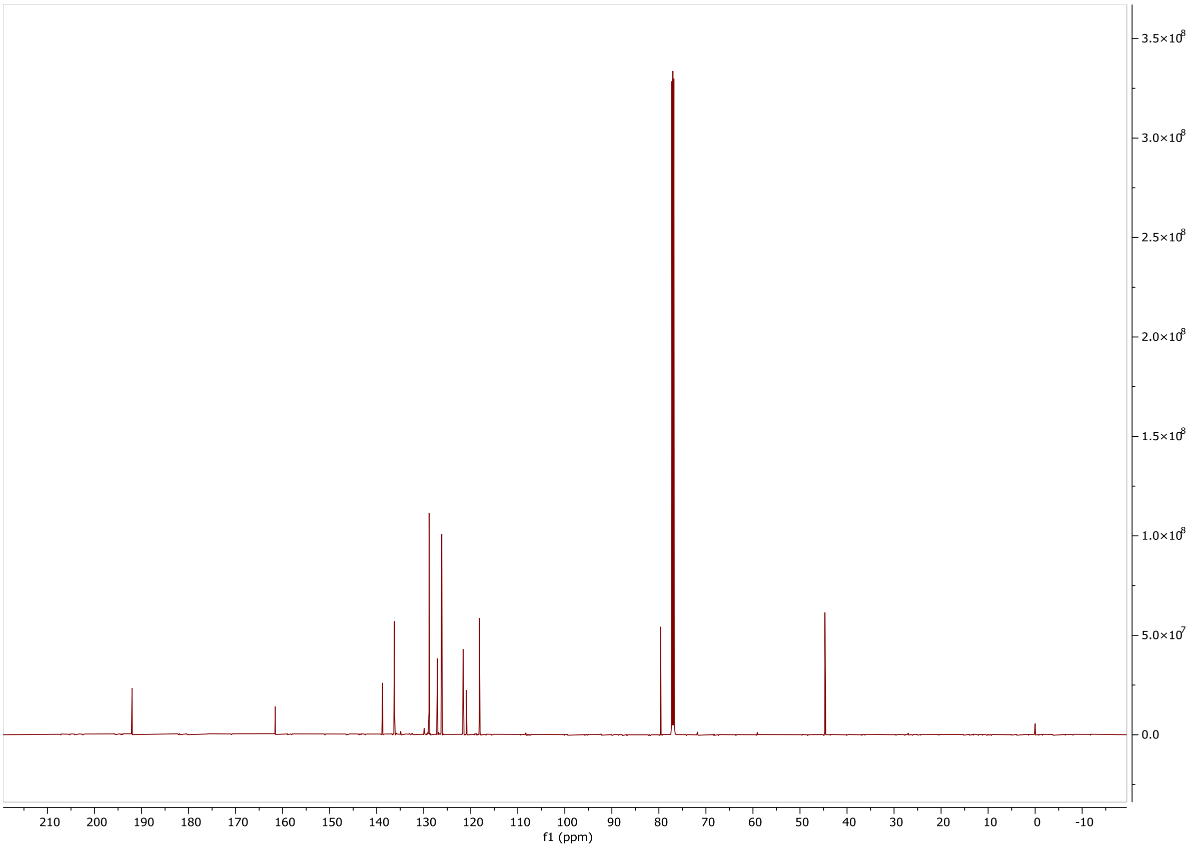

Figure S34. ^{13}C-NMR (150.91 MHz, $CHCl_3$-*d*) of compound 7.

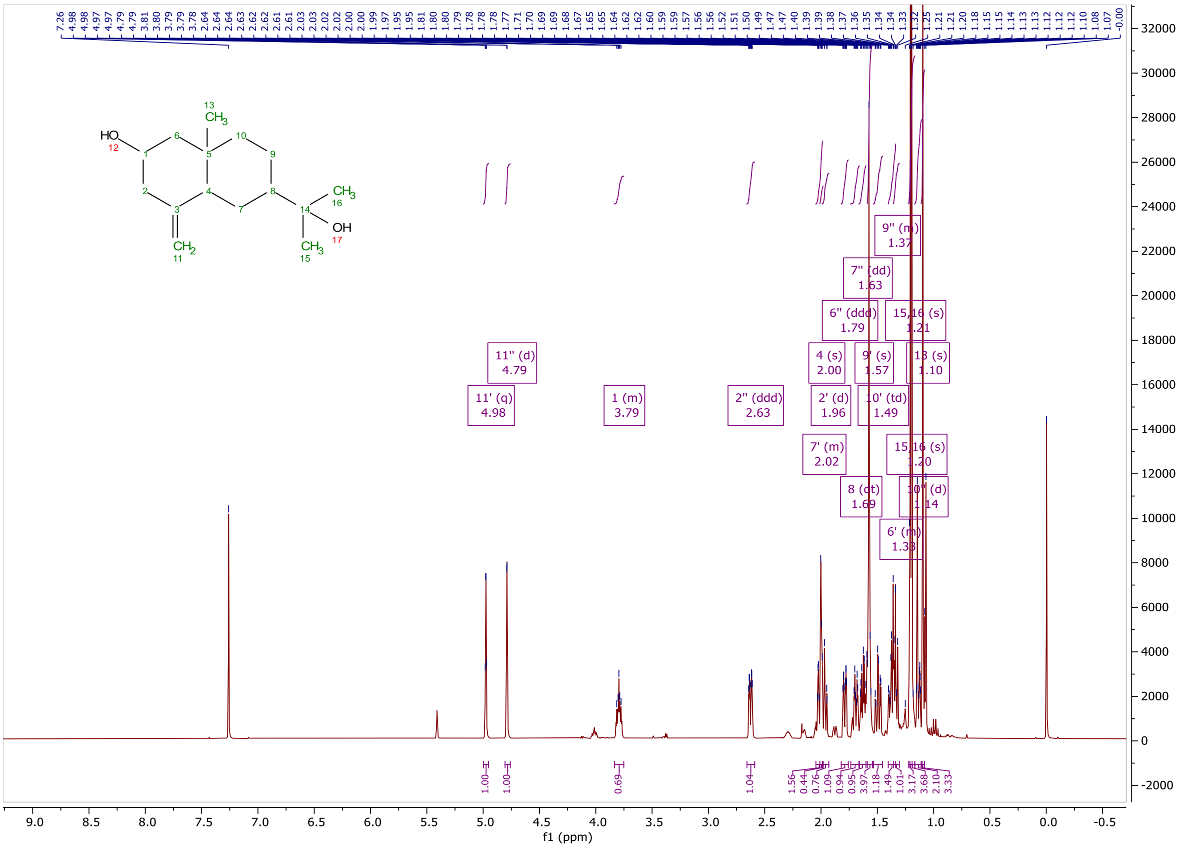

Figure S35. ^{1}H-NMR (600.19 MHz, $CHCl_3$-*d*) of compound 8.

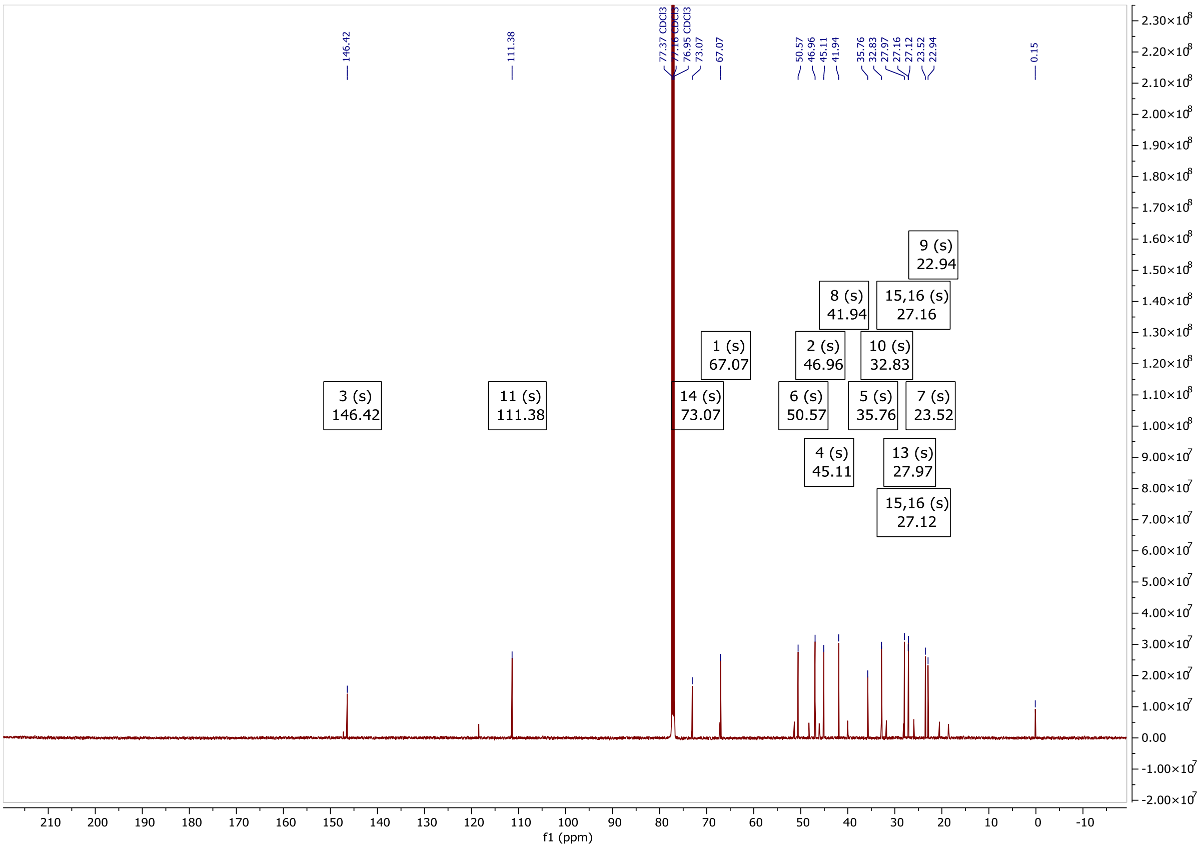

Figure S36. ^{13}**C-NMR (150.91 MHz, CHCl$_3$-*d*) of compound 8.**

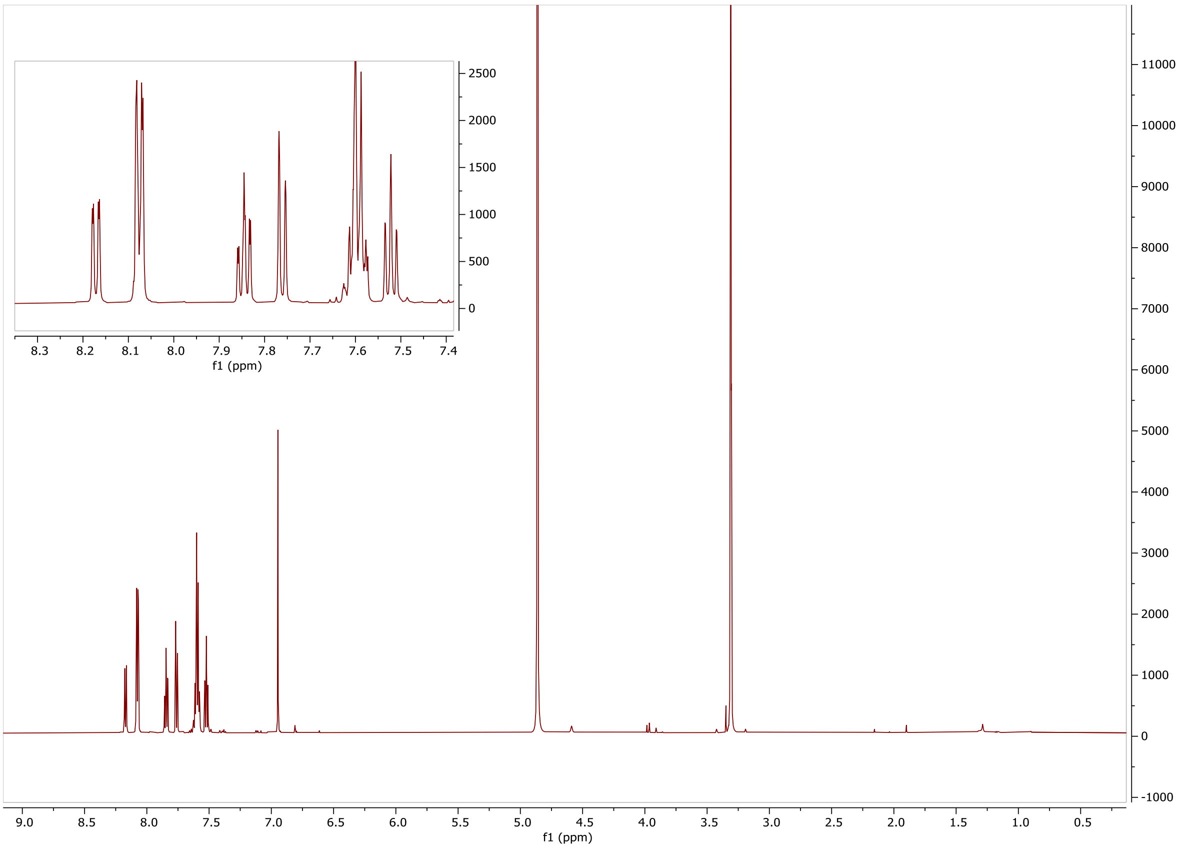

Figure S37. ^{1}H-NMR (600.19 MHz, MeOH-*d*$_4$) of compound 9.

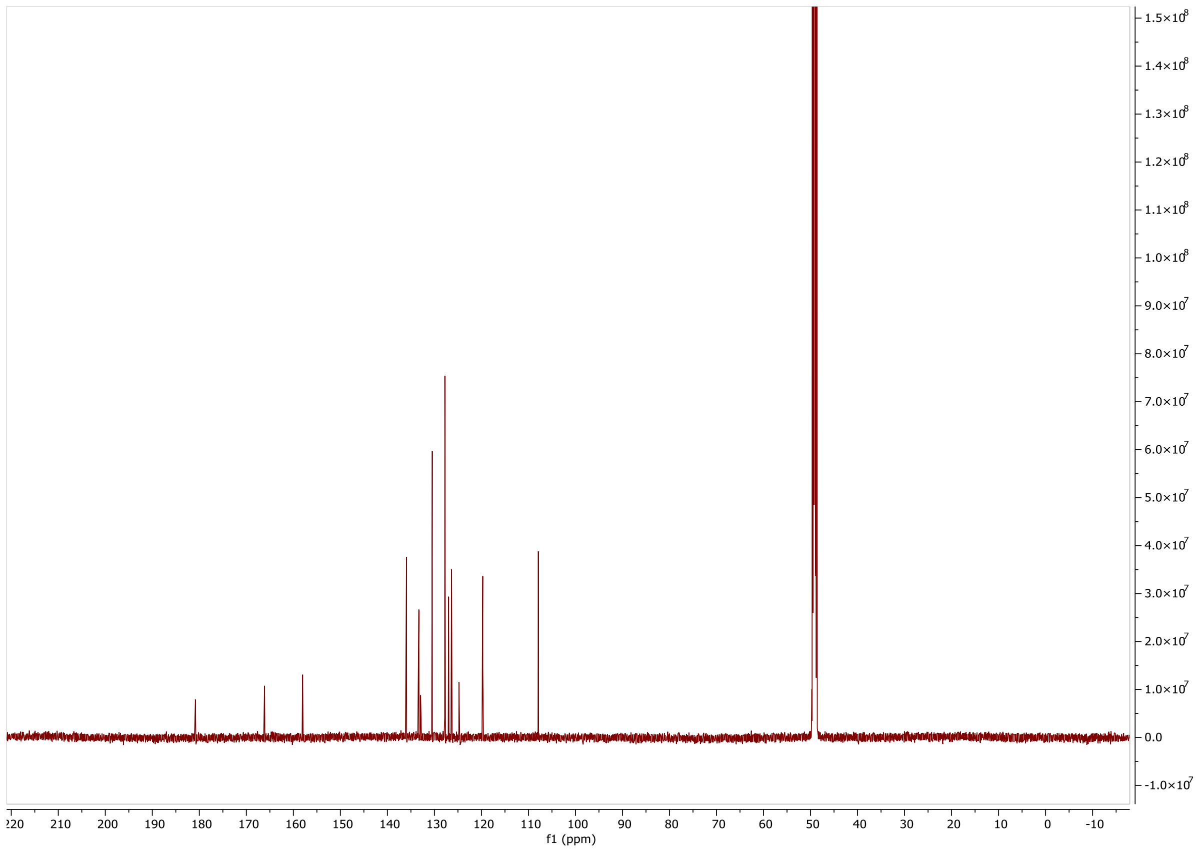

Figure S38. ^{13}C-NMR (150.91 MHz, MeOH-*d*$_4$) of compound 9.

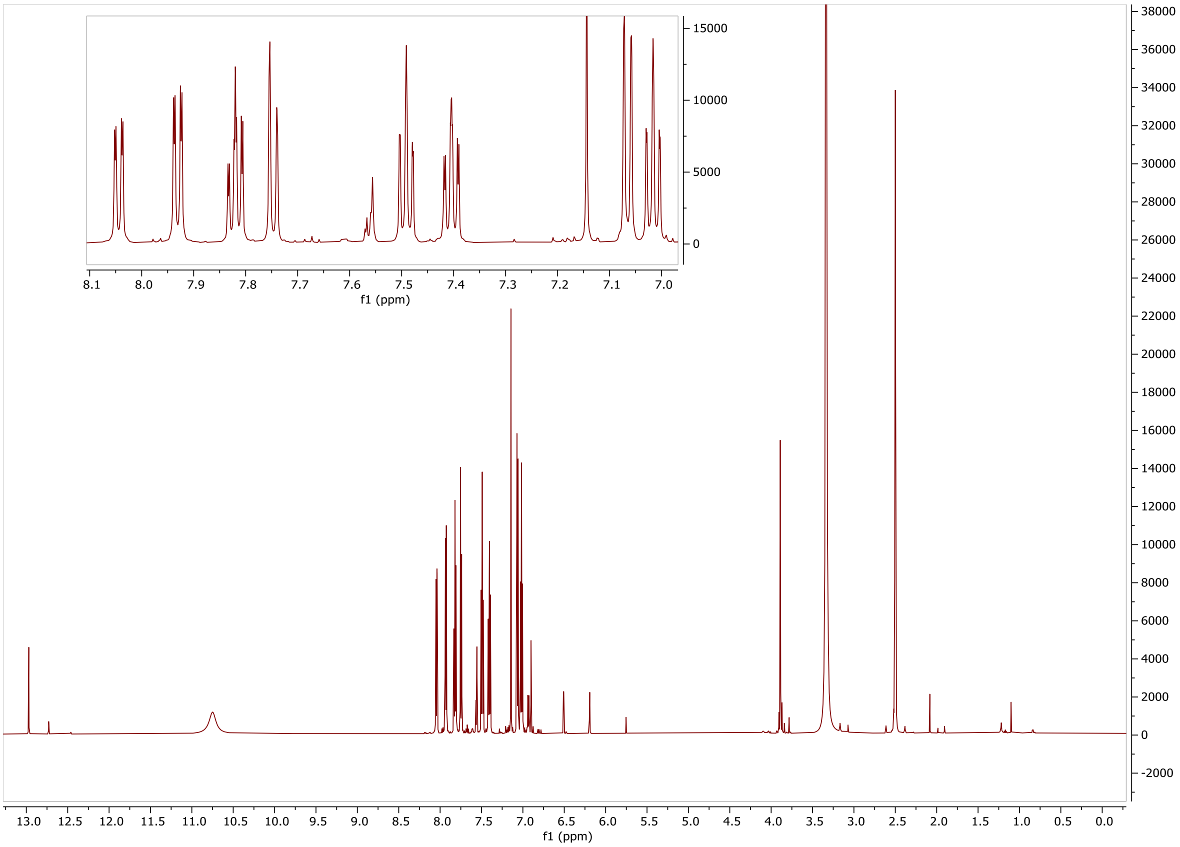

Figure S39. **^{1}H-NMR (600.19 MHz, DMSO-d_6) of compound 10.**

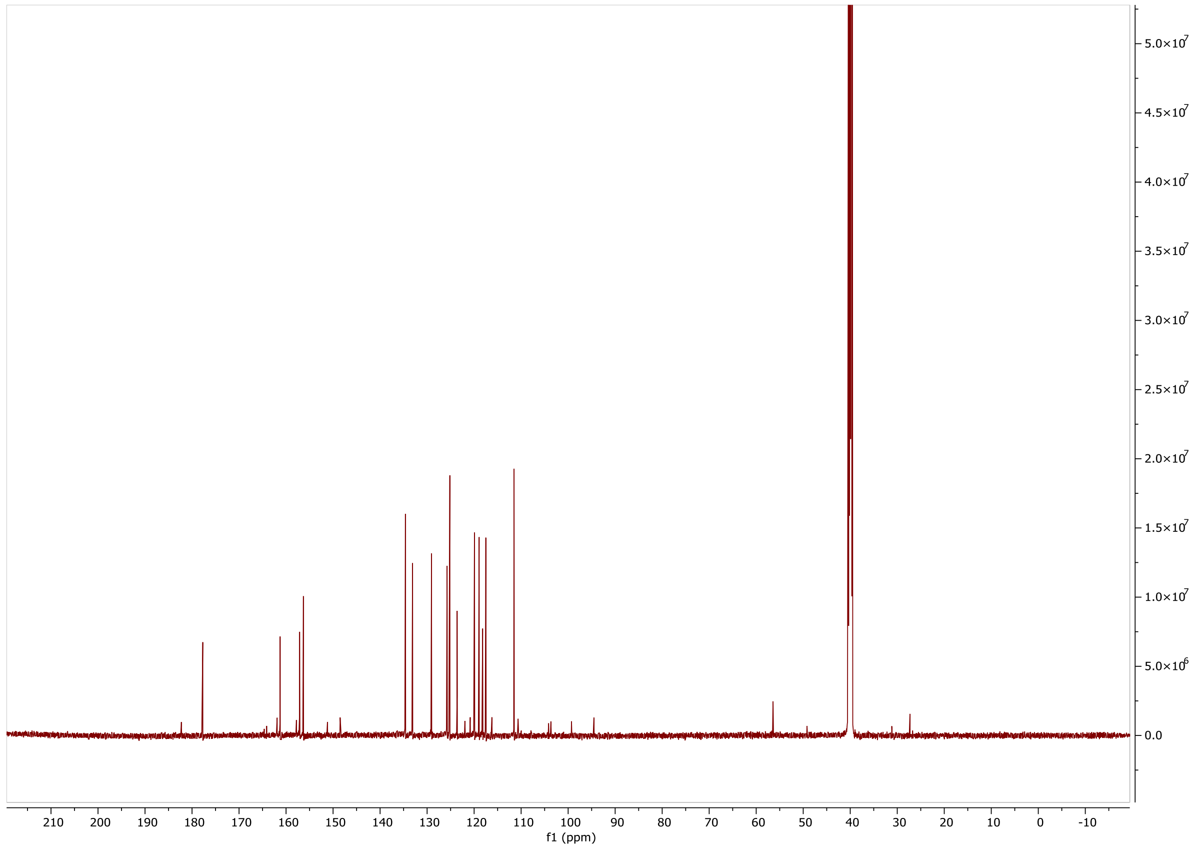

Figure S40. ^{13}C-NMR (150.91 MHz, DMSO-d_6) of compound 10.

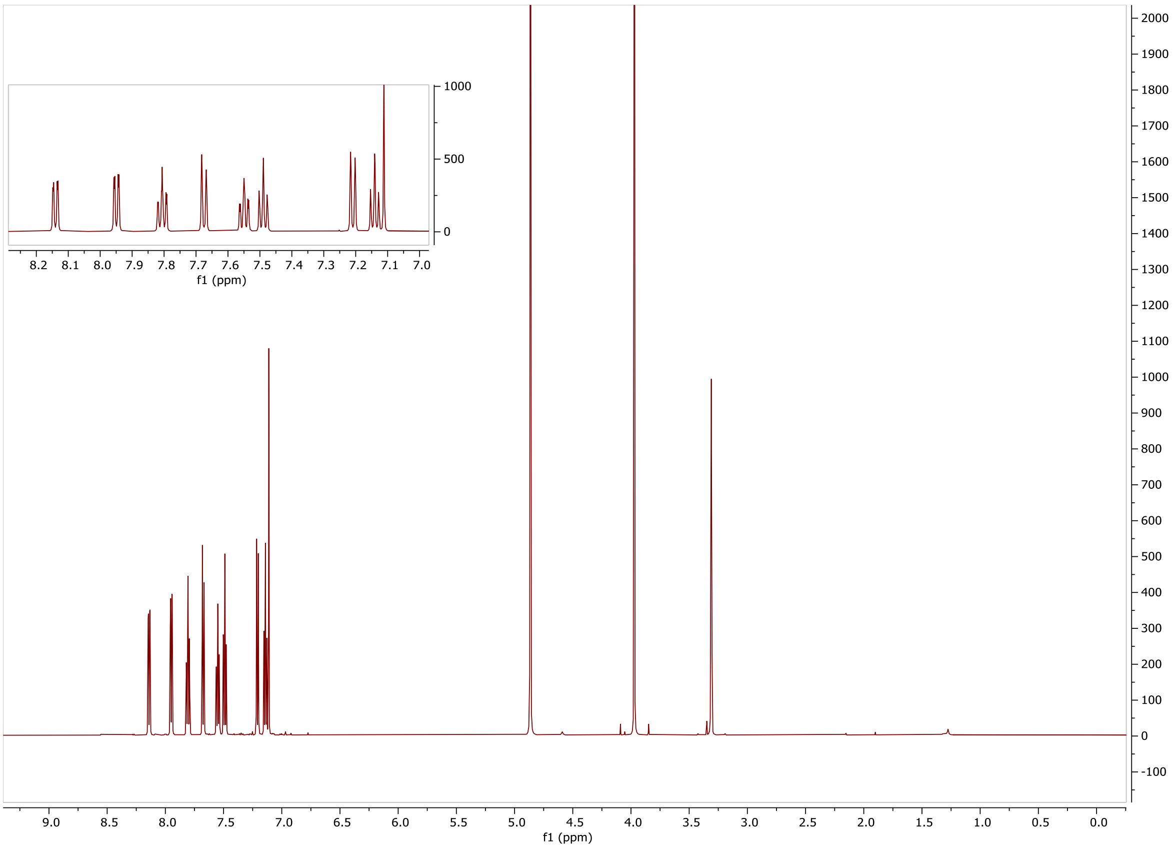

Figure S41. ^{1}H-NMR (600.19 MHz, MeOH-d_4) of compound 11.

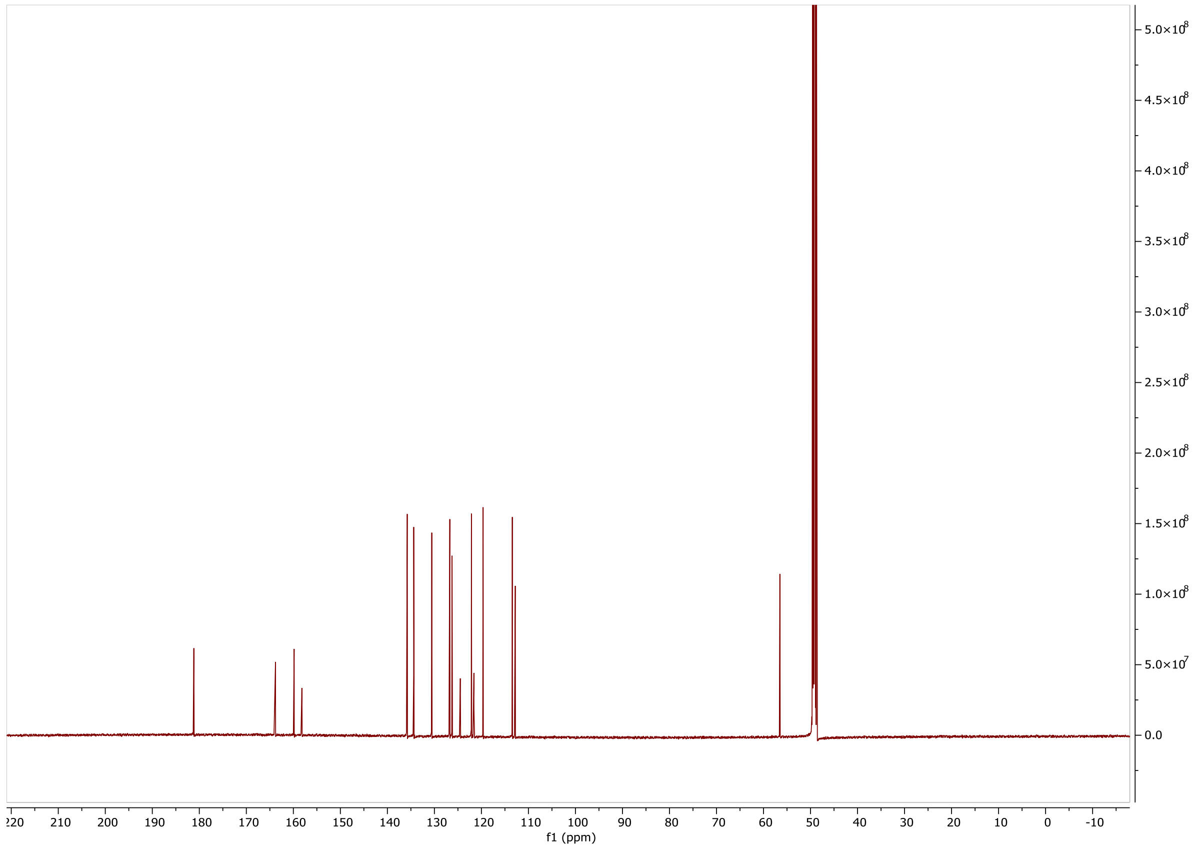

Figure S42. ^{13}**C-NMR (150.91 MHz, MeOH-*d*$_4$) of compound 11.**

Figure S43. ¹H-NMR (600.19 MHz, MeOH-d_4) of compound 12.

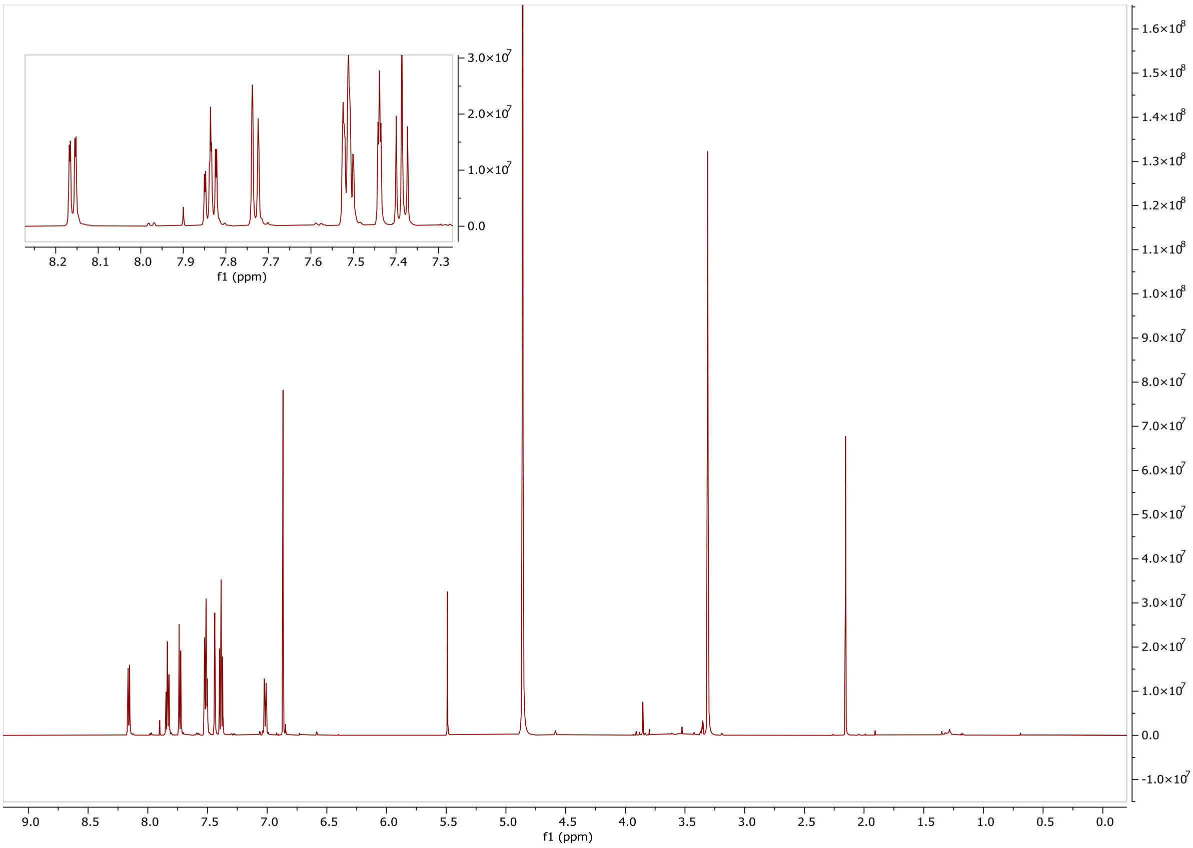

Figure S44. ^{13}**C-NMR (150.91 MHz, MeOH-*d*$_4$) of compound 12.**

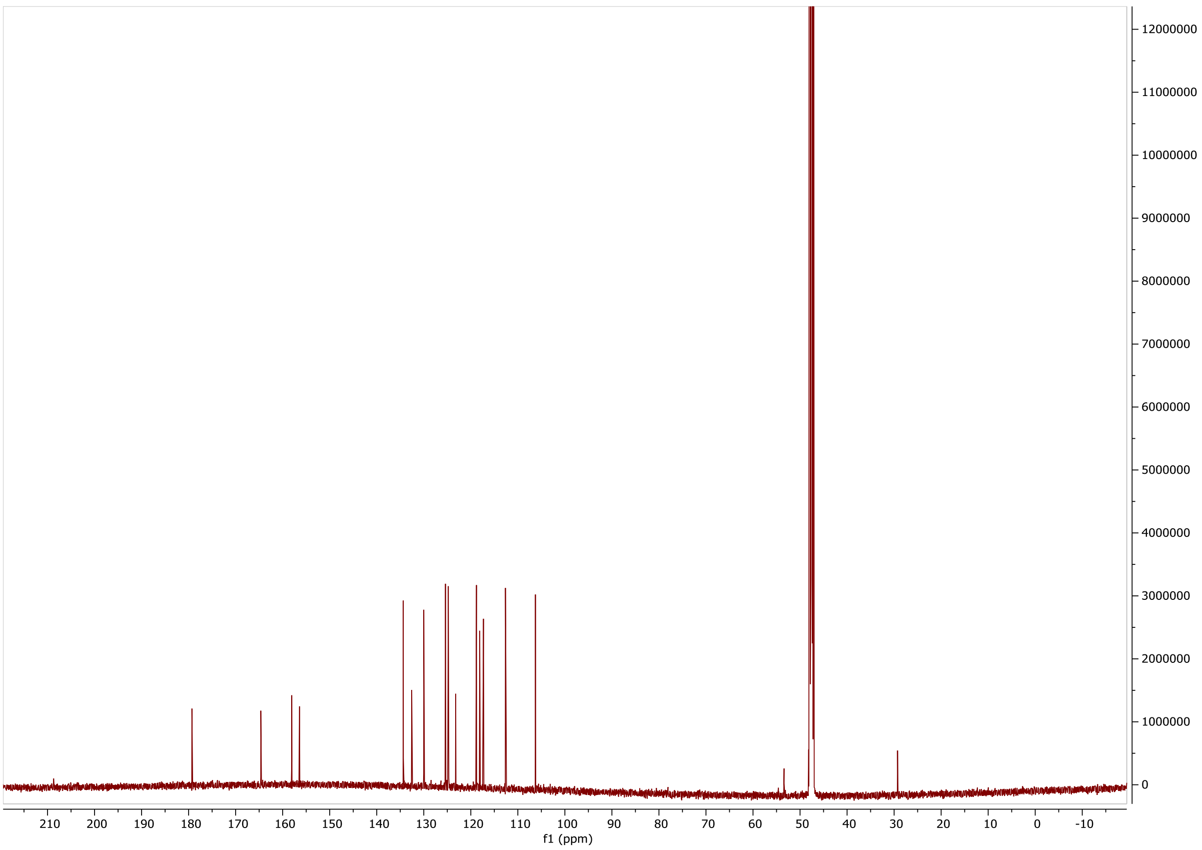

Figure S45. ^{1}H-NMR (600.19 MHz, MeOH-d_4) of compound 13.

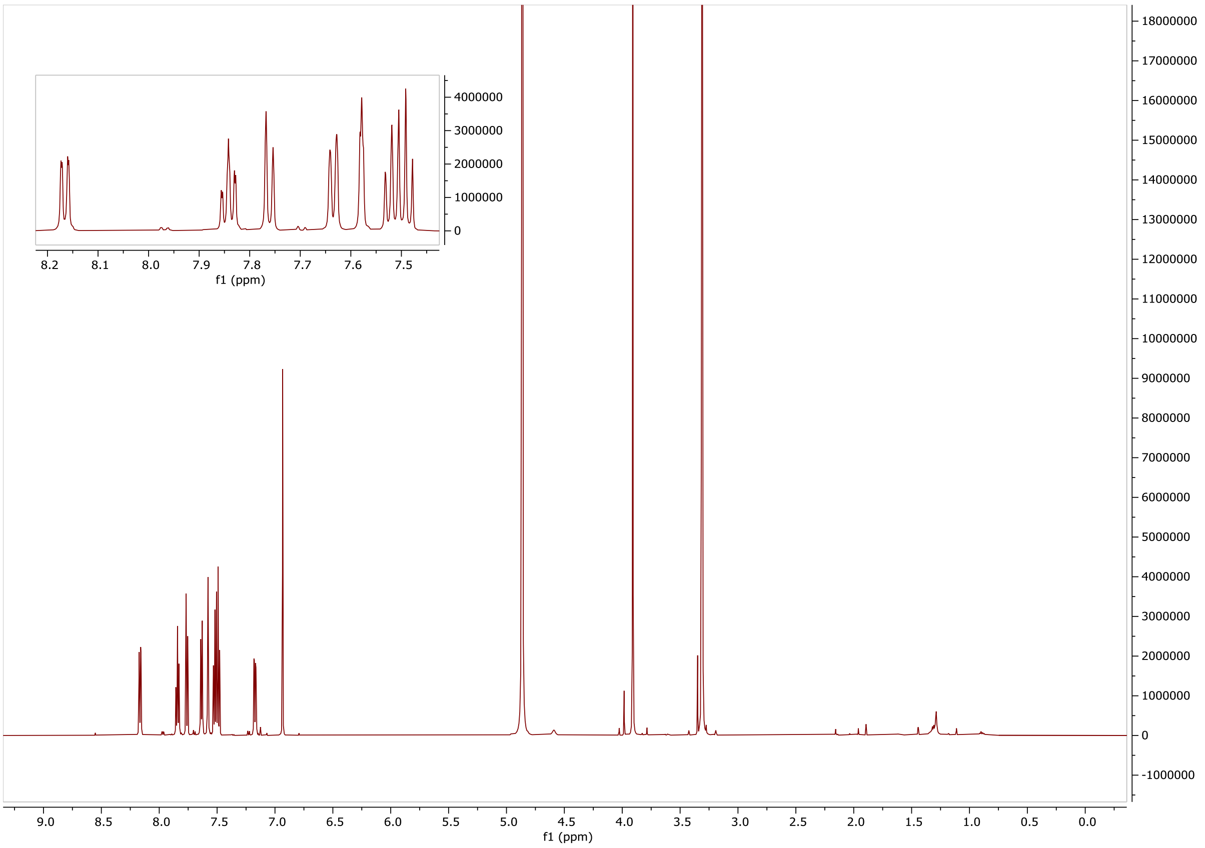

Figure S46. ^{13}C-NMR (150.91 MHz, MeOH-d_4) of compound 13.

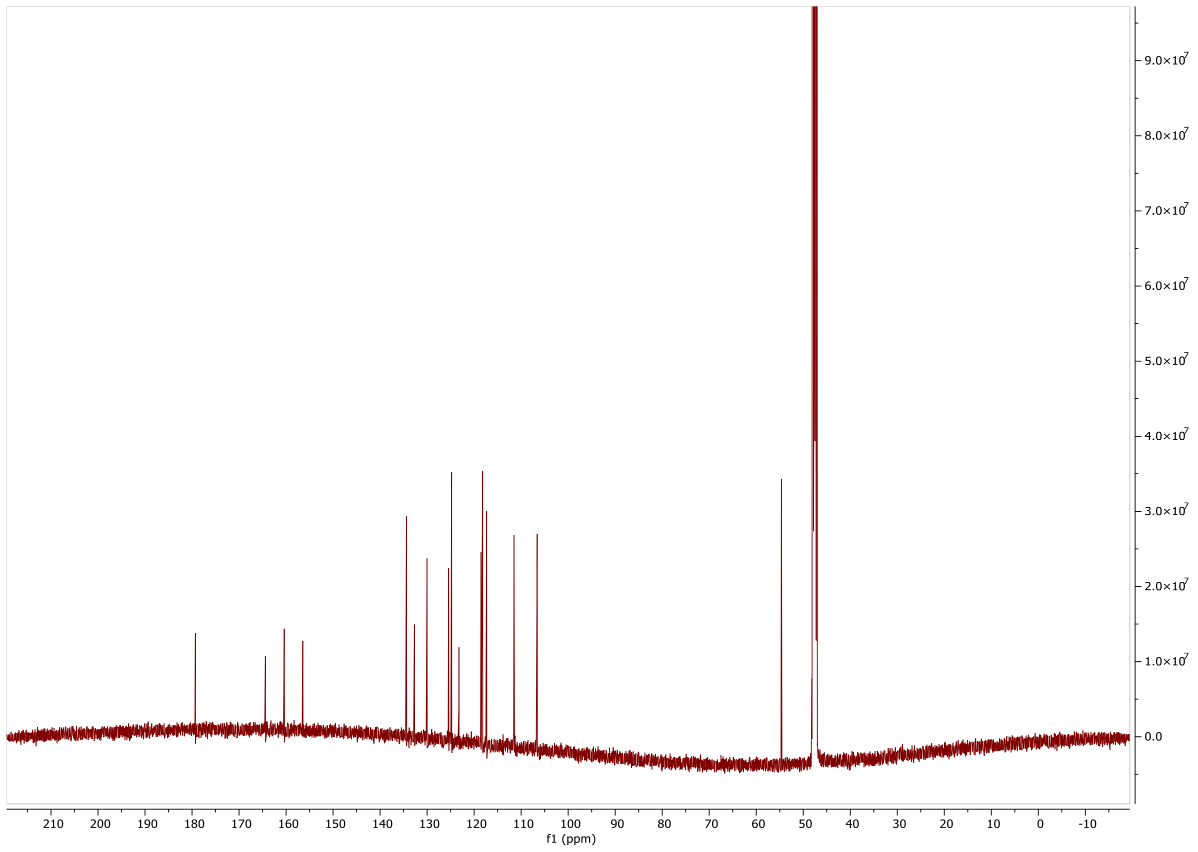

Figure S47. ^{1}H-NMR (600.19 MHz, Acetone-d_6) of compound 14.

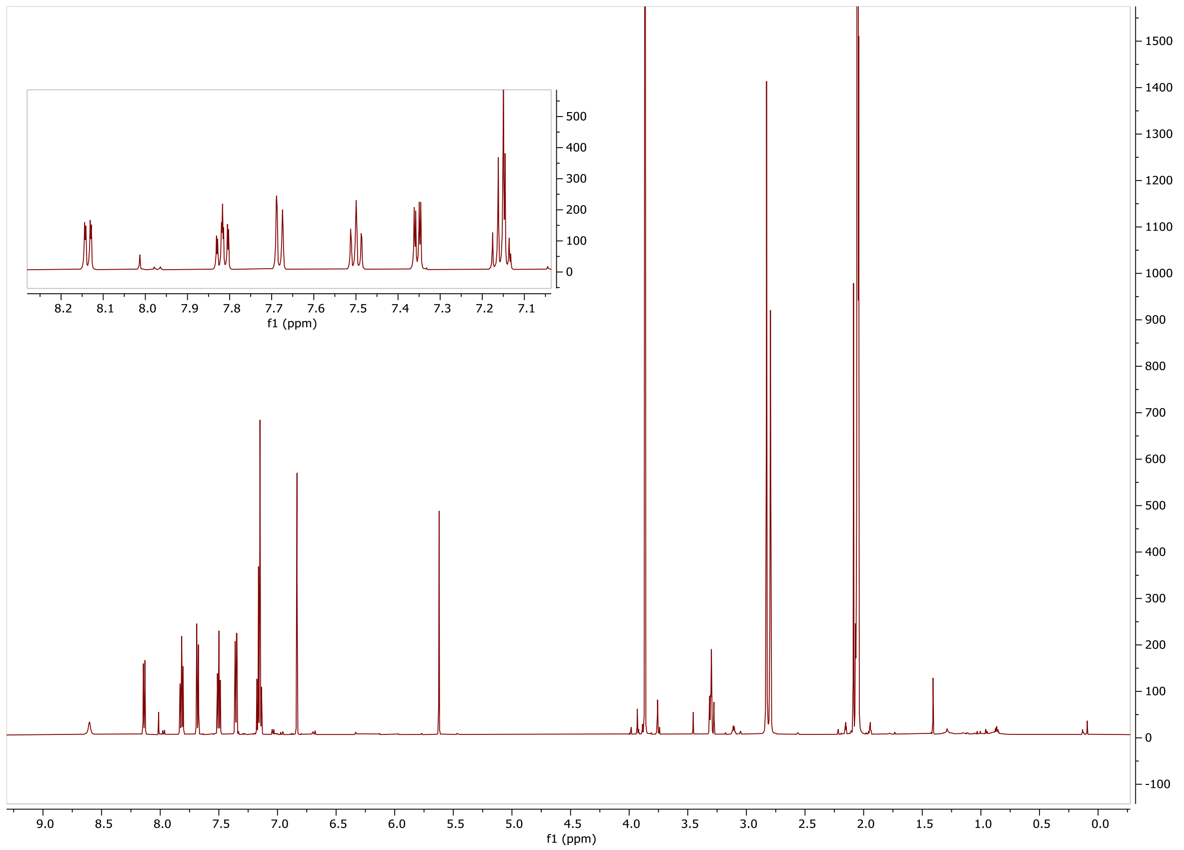

Figure S48. ^{13}C-NMR (150.91 MHz, Acetone-d_6) of compound 14.

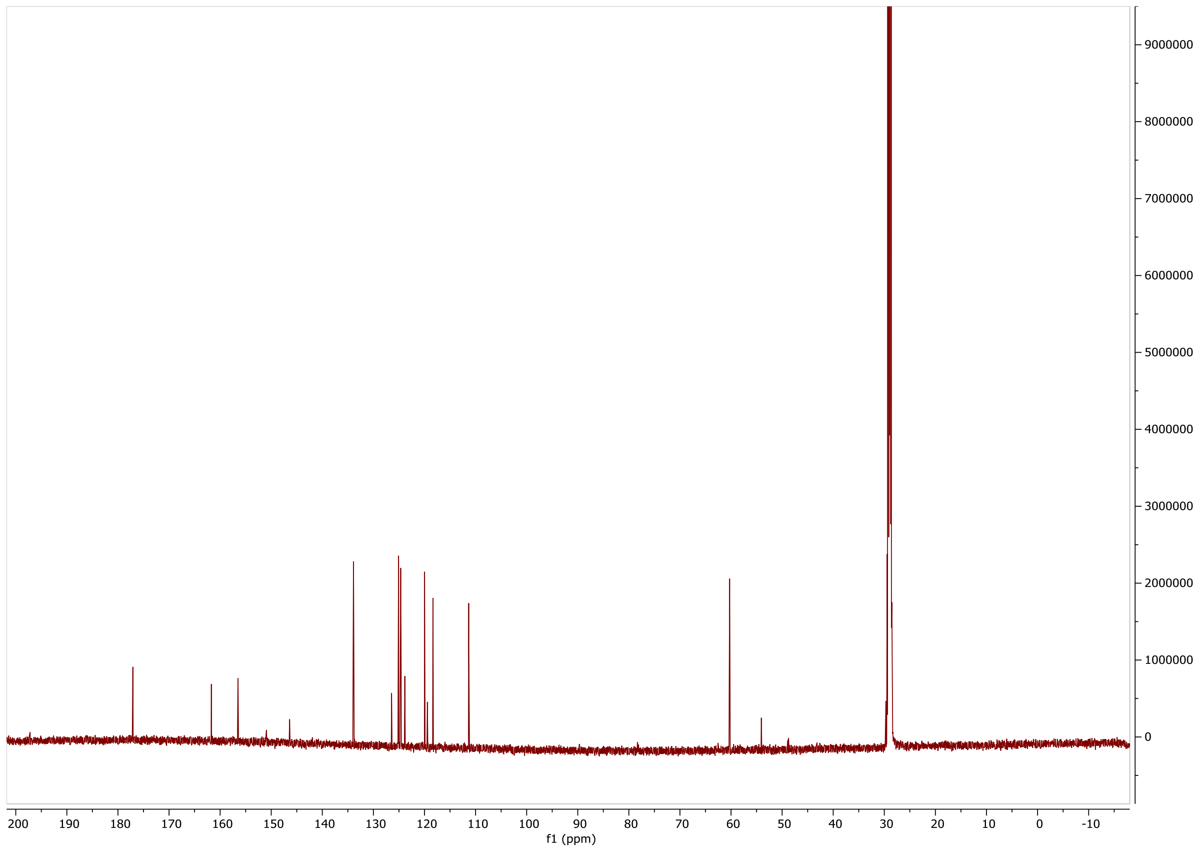

Figure S49. ^{1}H-NMR (600.19 MHz, DMSO-d_6) of compound 15.

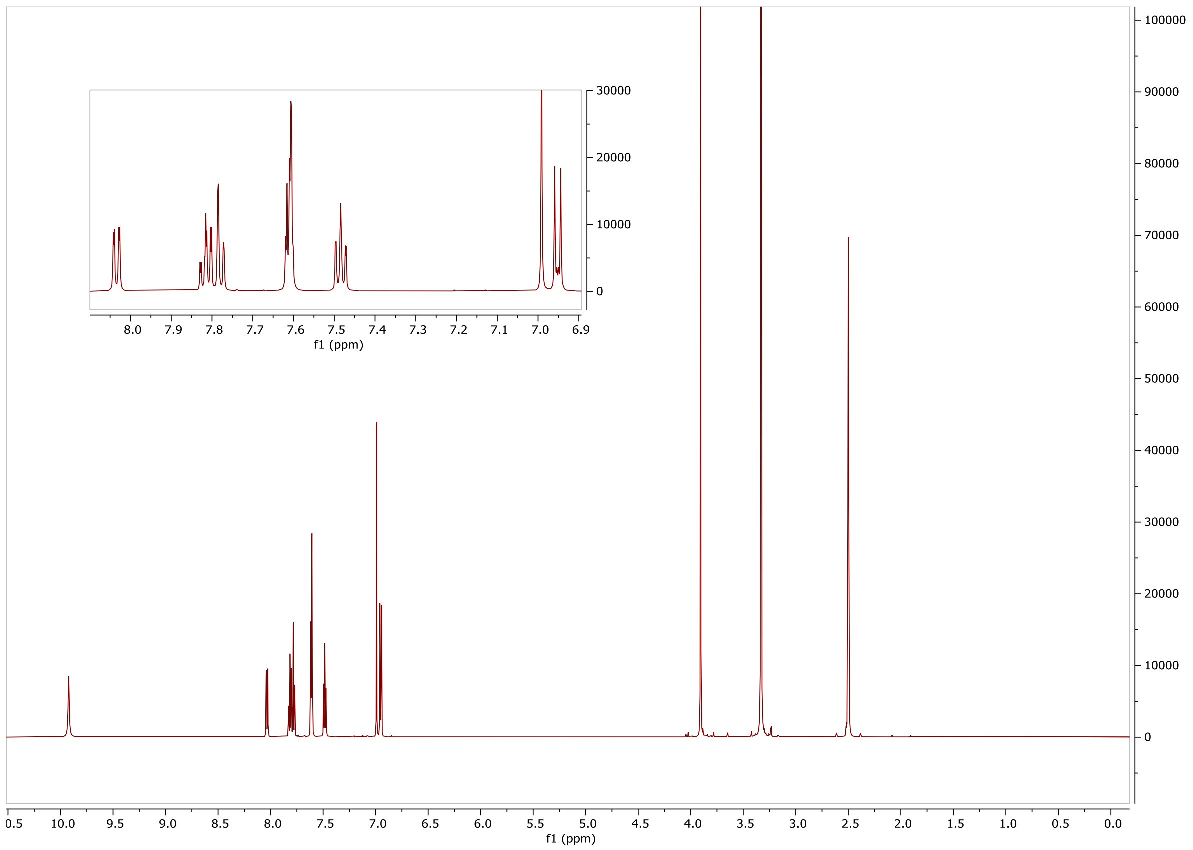

Figure S50. ^{13}C-NMR (150.91 MHz, DMSO-d_6) of compound 15.

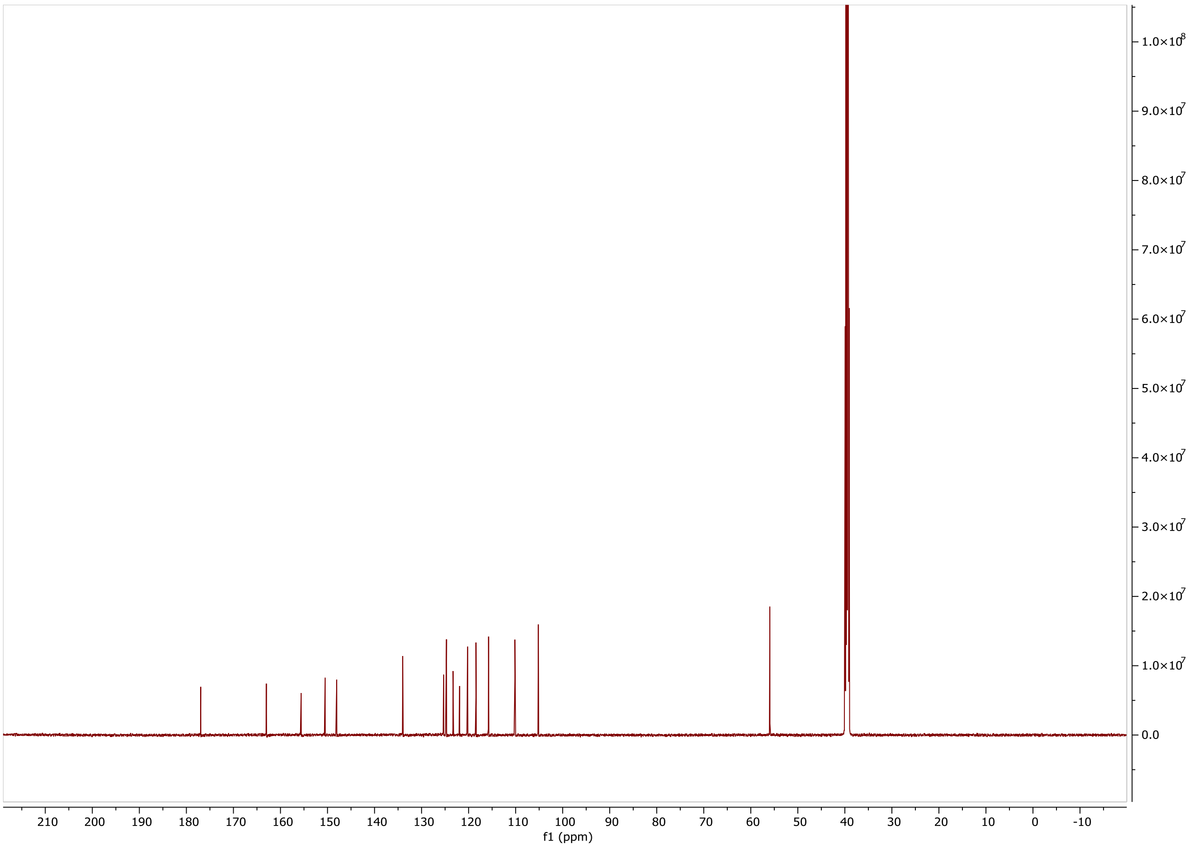

Figure S51. ¹H-NMR (600.19 MHz, DMSO-d_6) of compound 16.

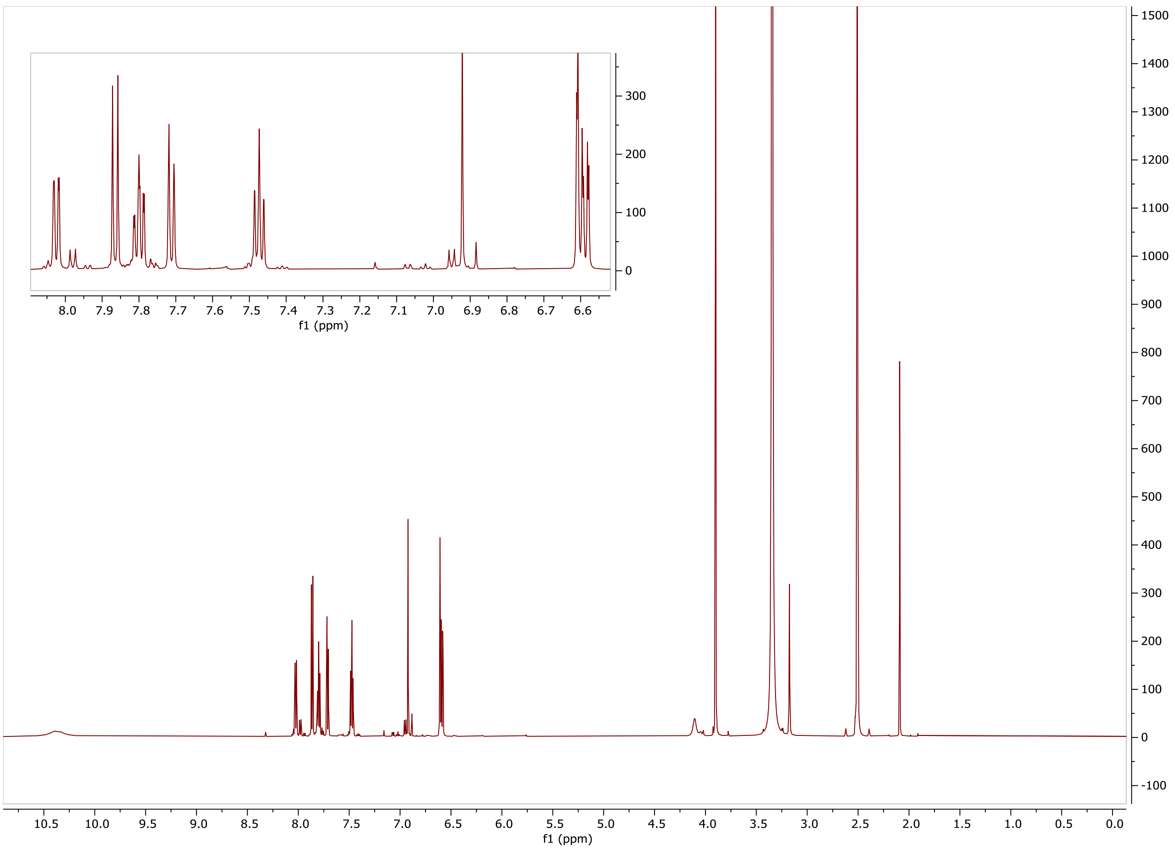

Figure S52. ^{13}C-NMR (150.91 MHz, DMSO-d_6) of compound 16.

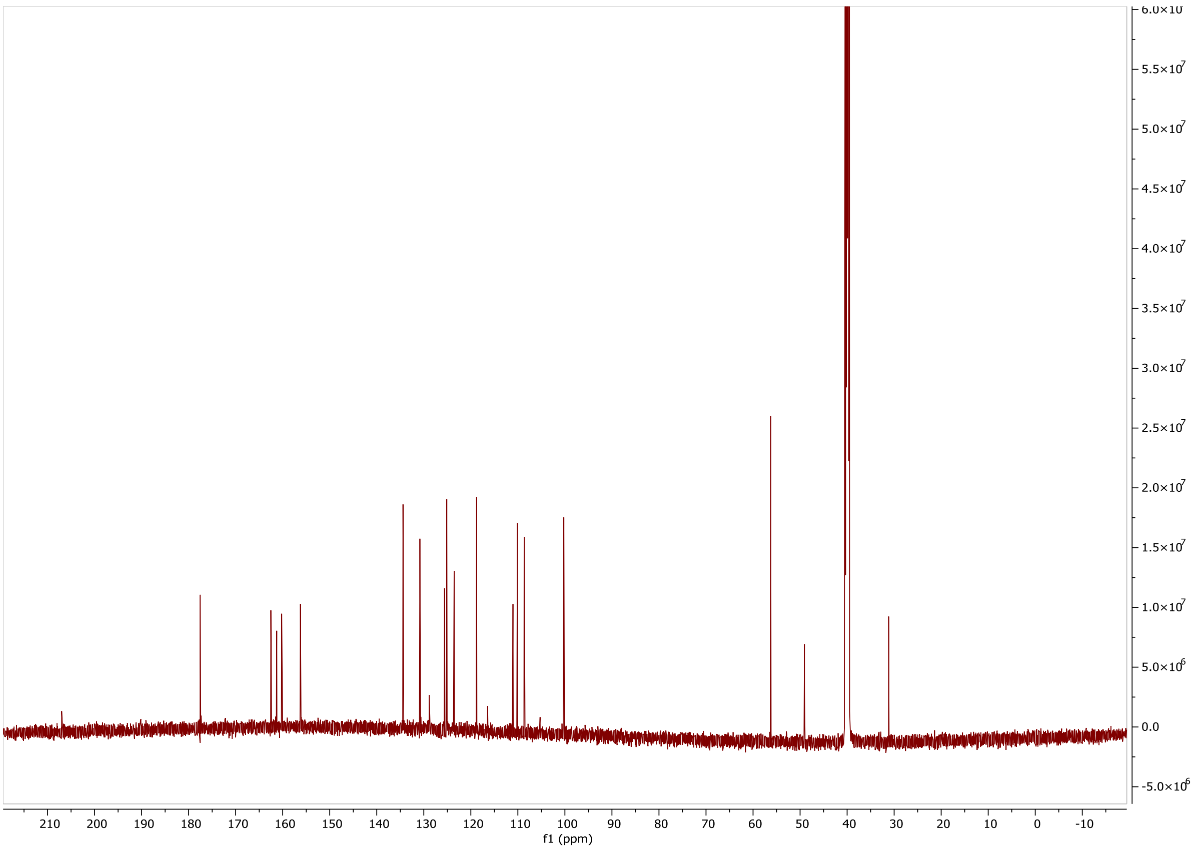

Figure S53. ¹H-NMR (600.19 MHz, $CHCl_3$-*d*) of compound 17.

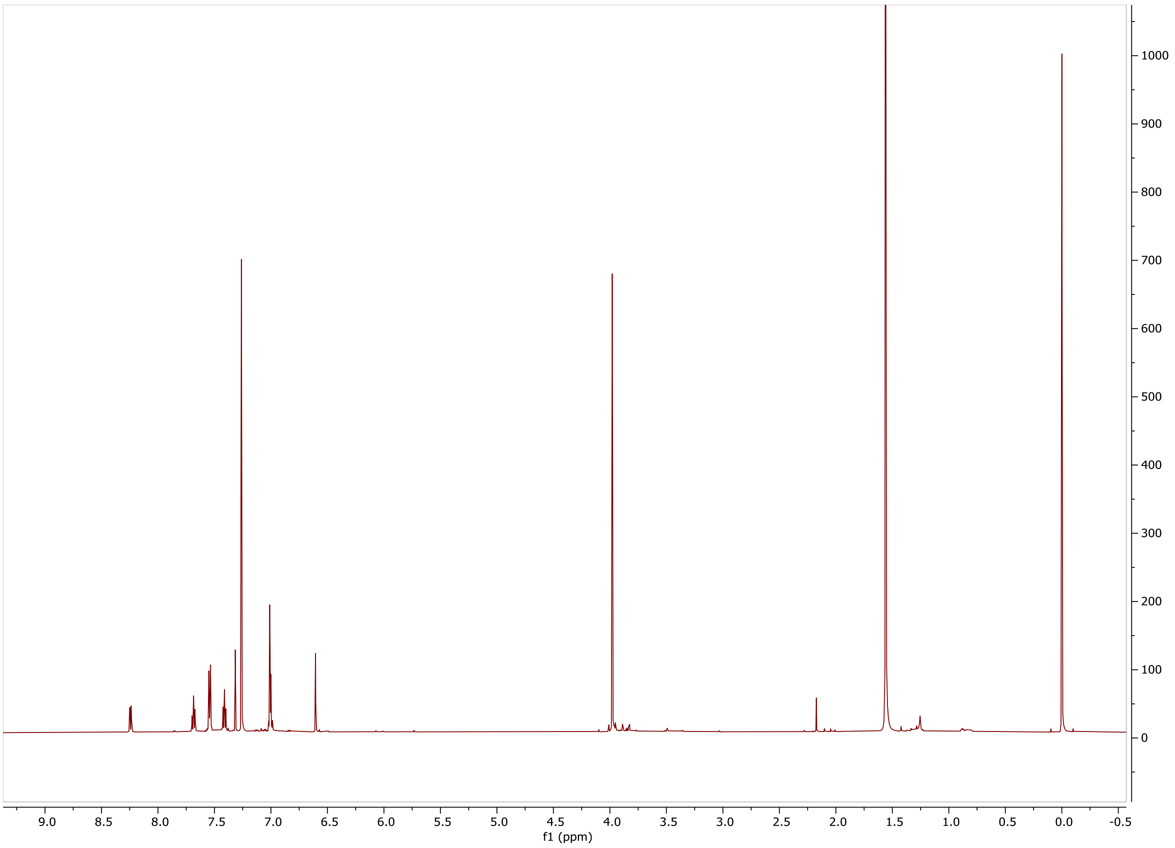

Figure S54. ^{13}**C-NMR (150.91 MHz, CHCl**$_3$**-*d*) of compound 17.**

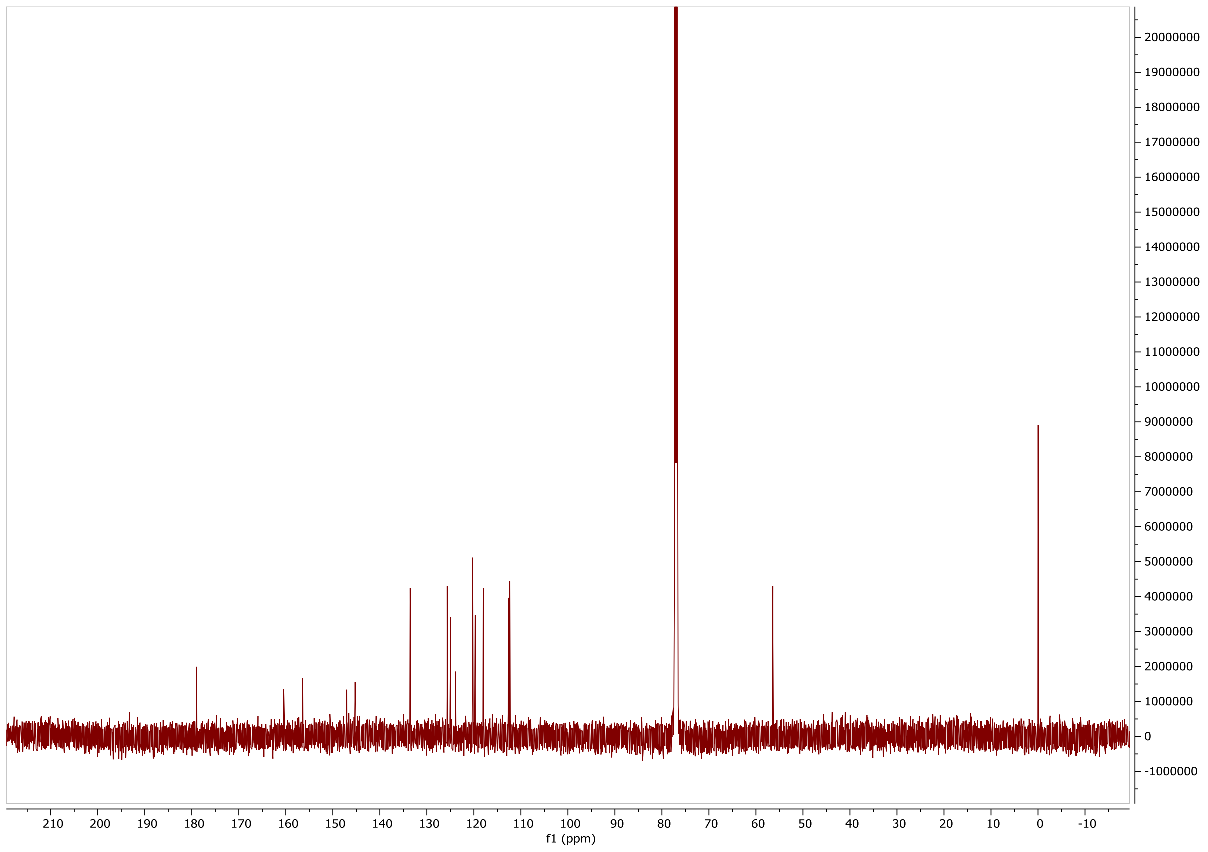

Figure S55. ^{1}H-NMR (600.19 MHz, $CHCl_3$-*d*) of compound 18.

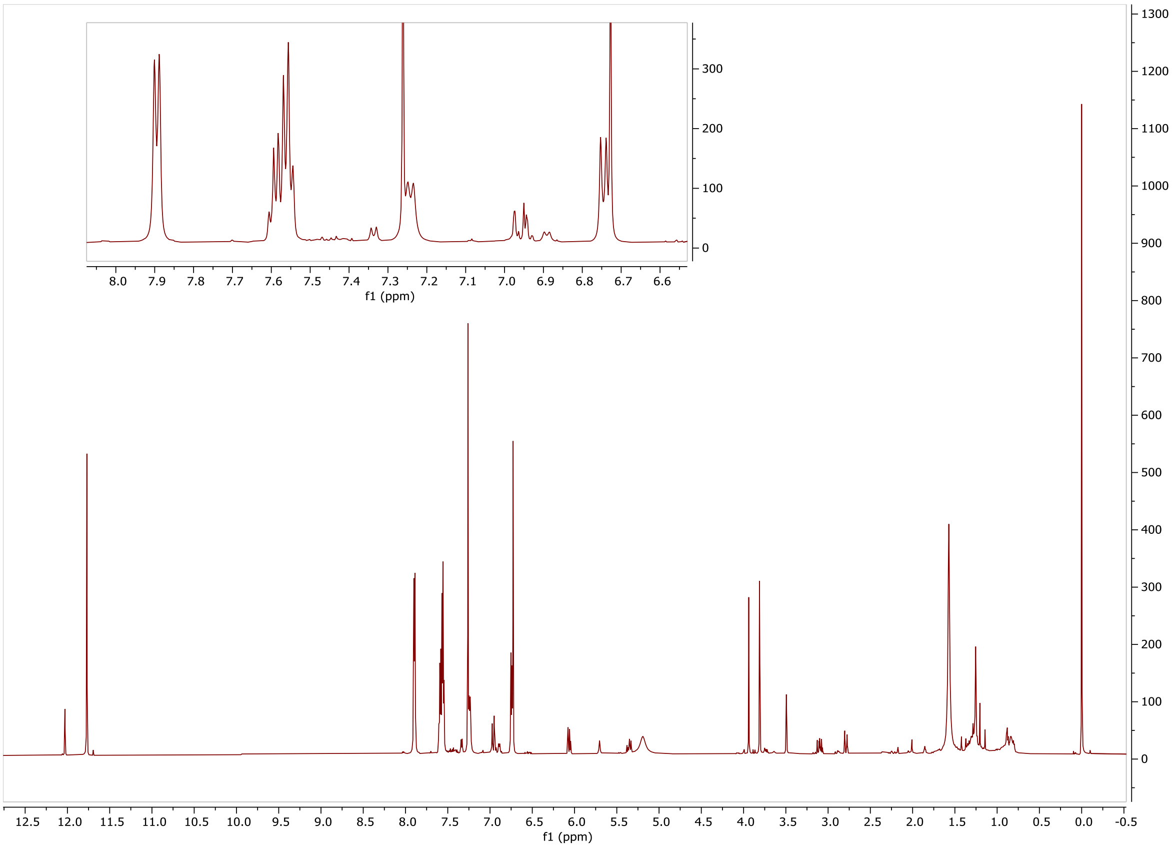

Figure S56. ^{13}**C-NMR (150.91 MHz, CHCl**$_3$**-*d*) of compound 18.**

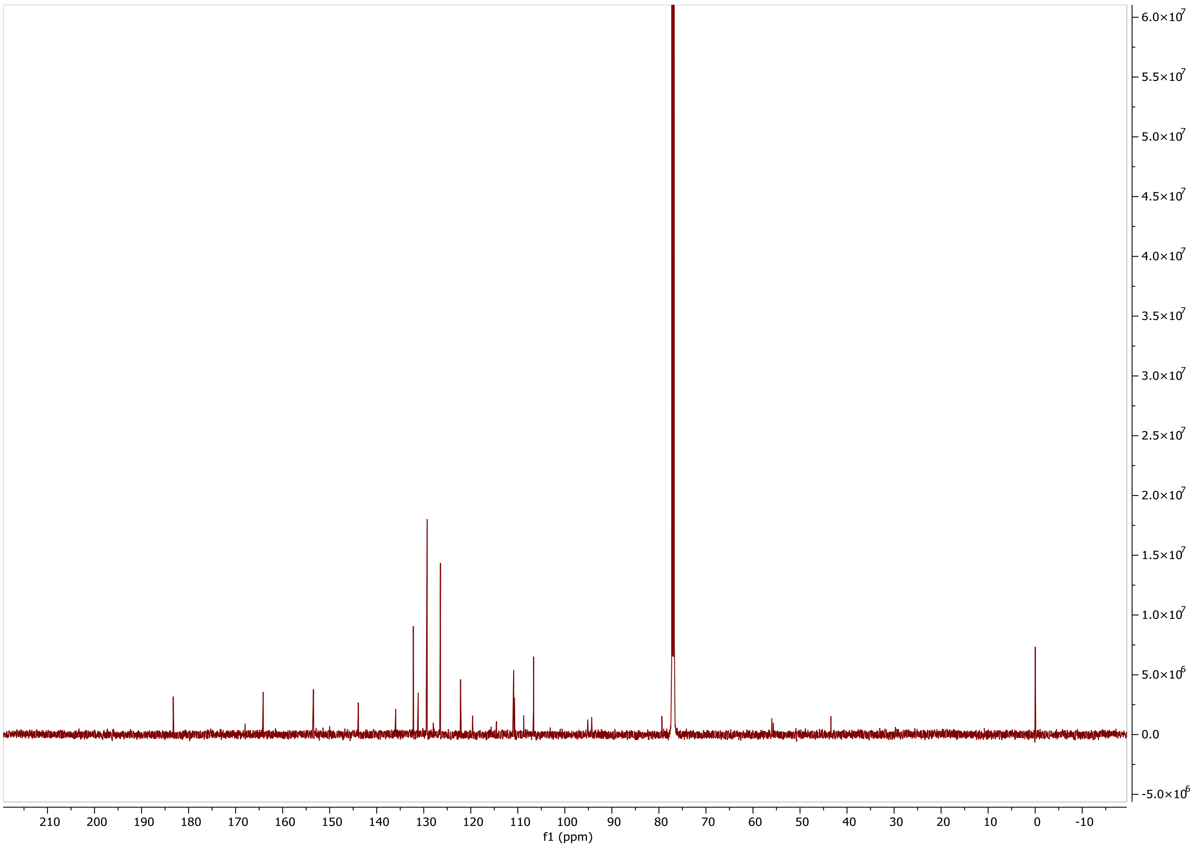

Figure S57. ^{1}H-NMR (600.19 MHz, DMSO-d_6) of compound 19.

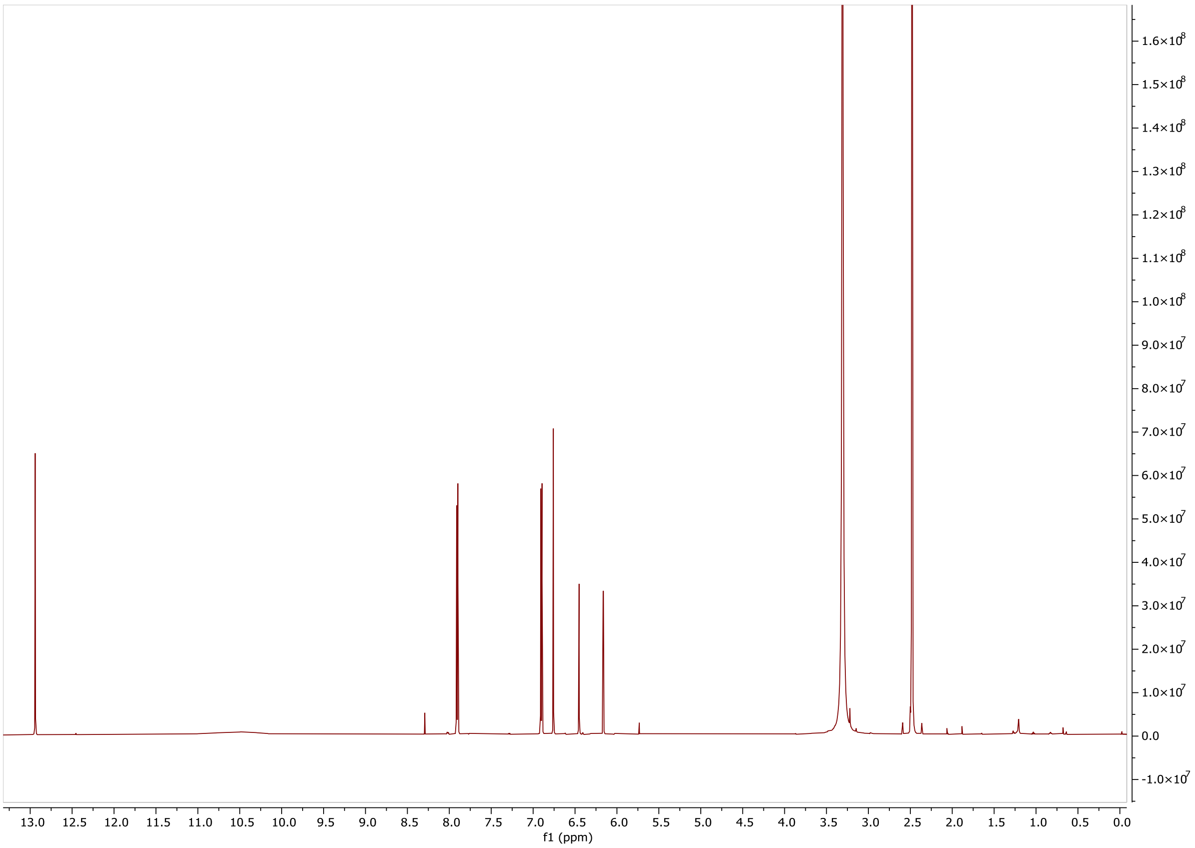

Figure S58. ^{13}C-NMR (150.91 MHz, DMSO-d_6) of compound 19.

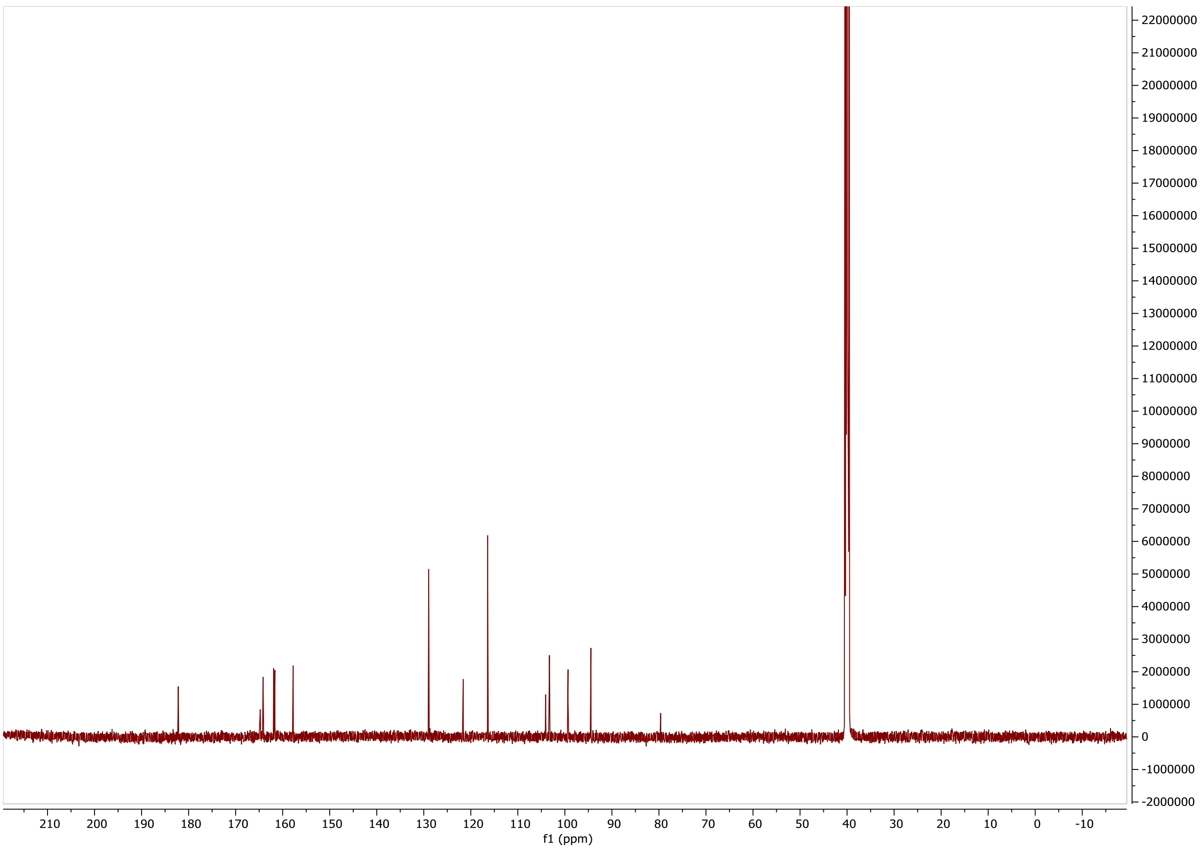

Figure S59. ^{1}H-NMR (600.19 MHz, DMSO-d_6) of compound 20.

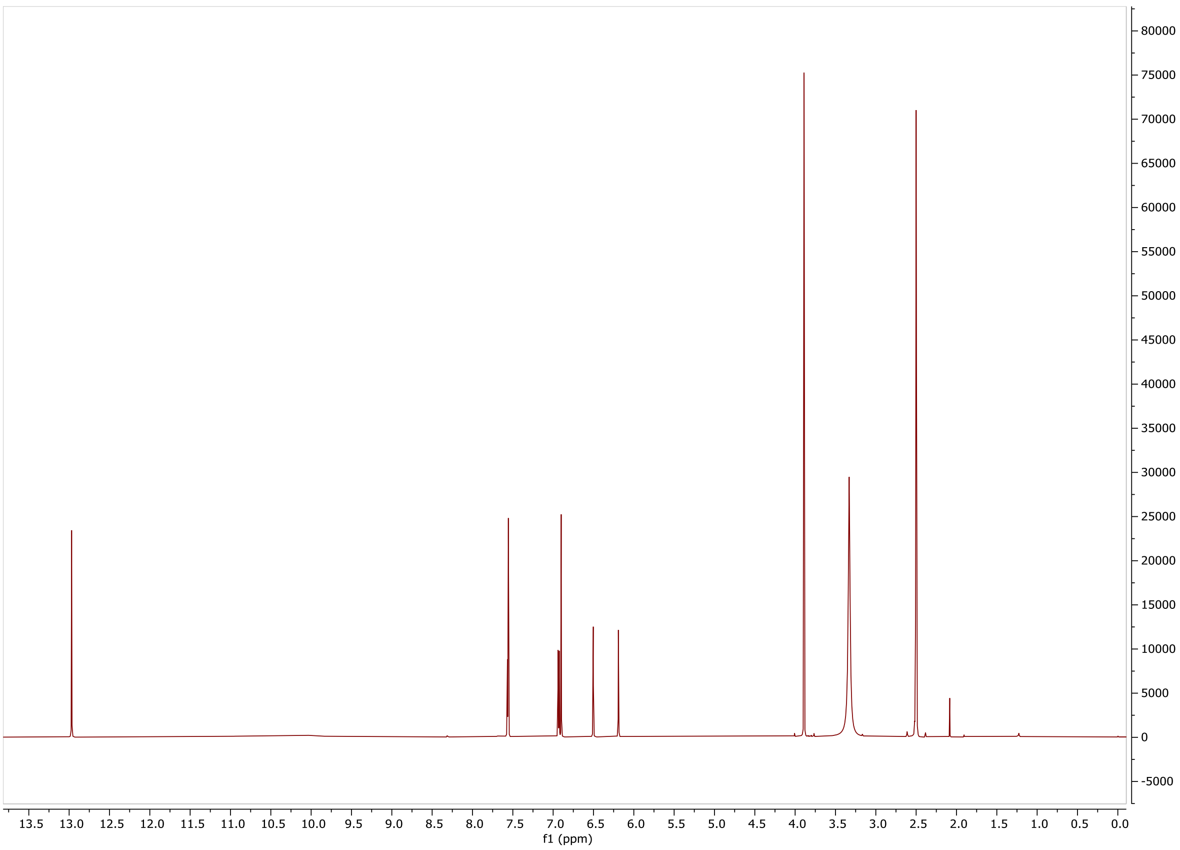

Figure S60. ^{13}C-NMR (150.91 MHz, DMSO-d_6) of compound 20.

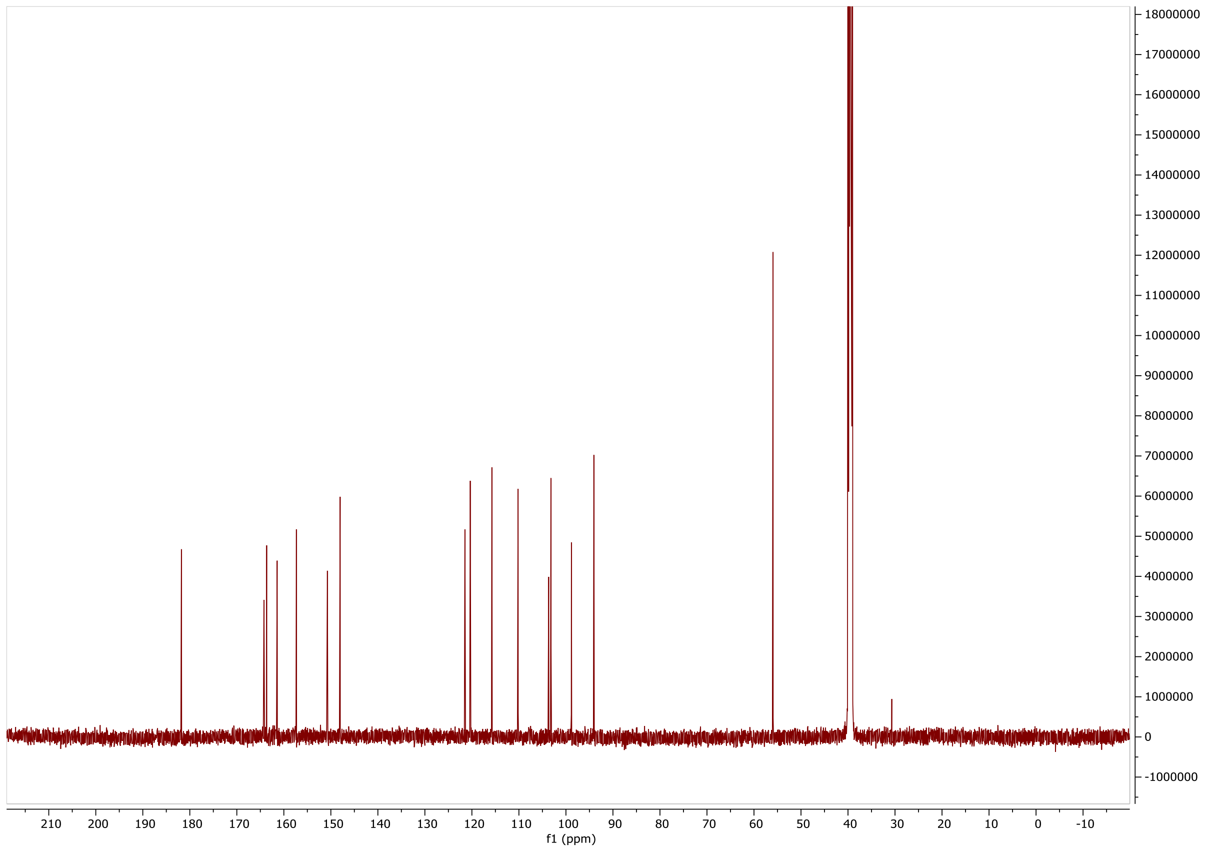

Figure S61. ^{1}H-NMR (600.19 MHz, MeOH-d_4) of compound 21.

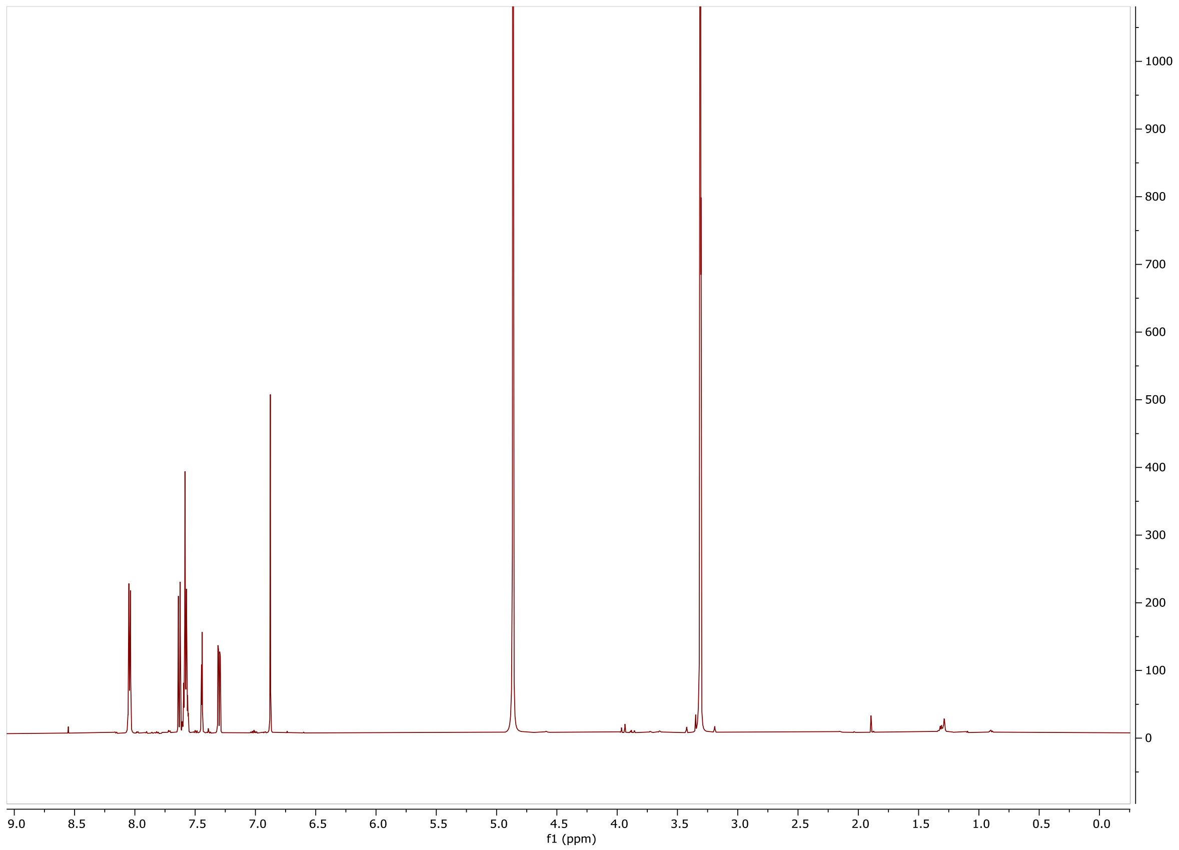

Figure S62. ^{13}**C-NMR (150.91 MHz, MeOH-*d*$_4$) of compound 21.**

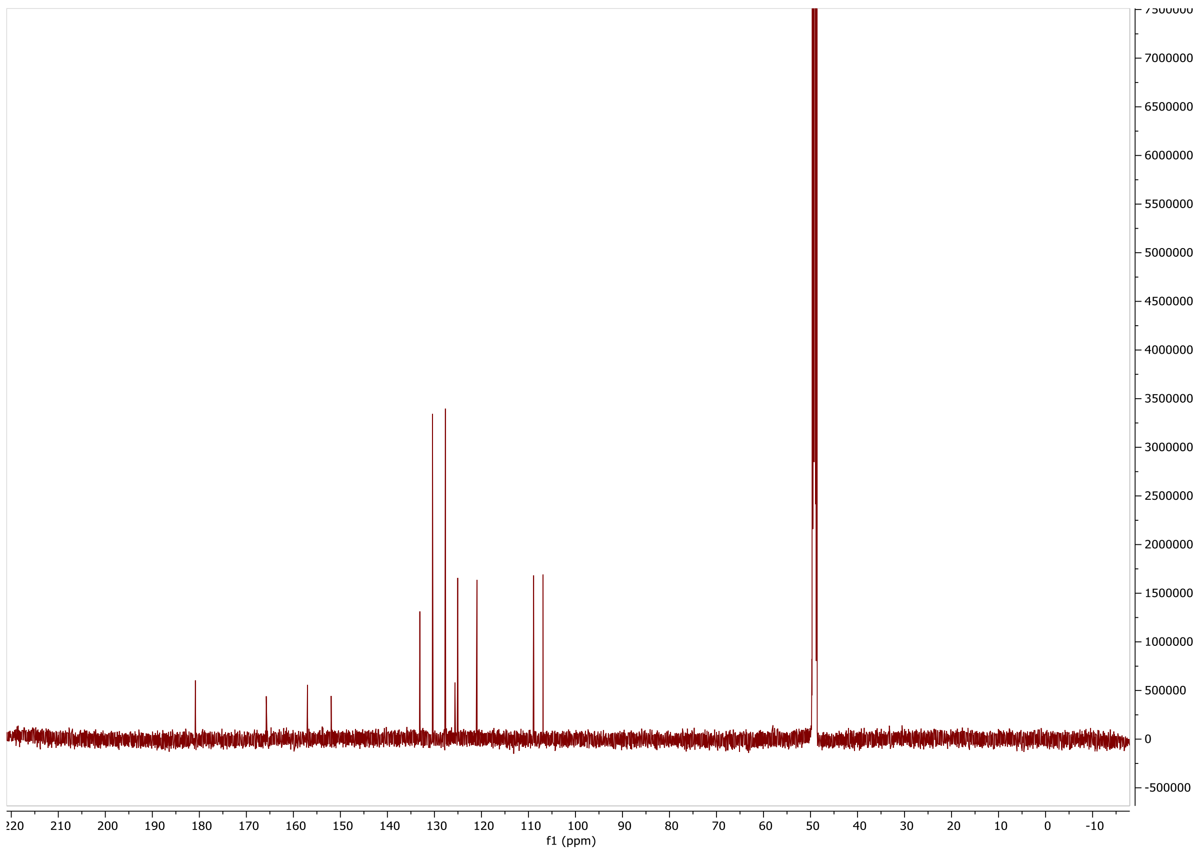

Figure S63. ^{1}H-NMR (600.19 MHz, $CHCl_3$-*d*) of compound 22.

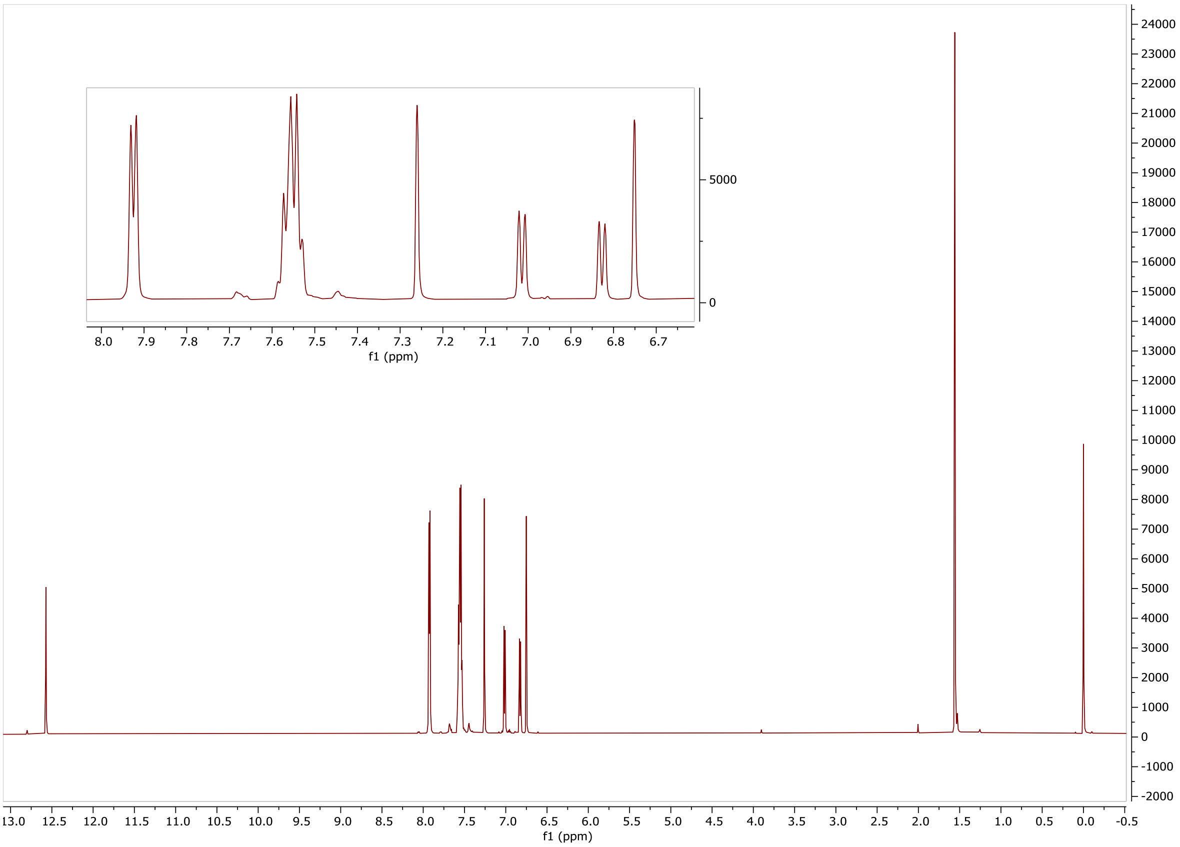

Figure S64. ^{13}**C-NMR (150.91 MHz, CHCl**$_3$**-*d*) of compound 22.**

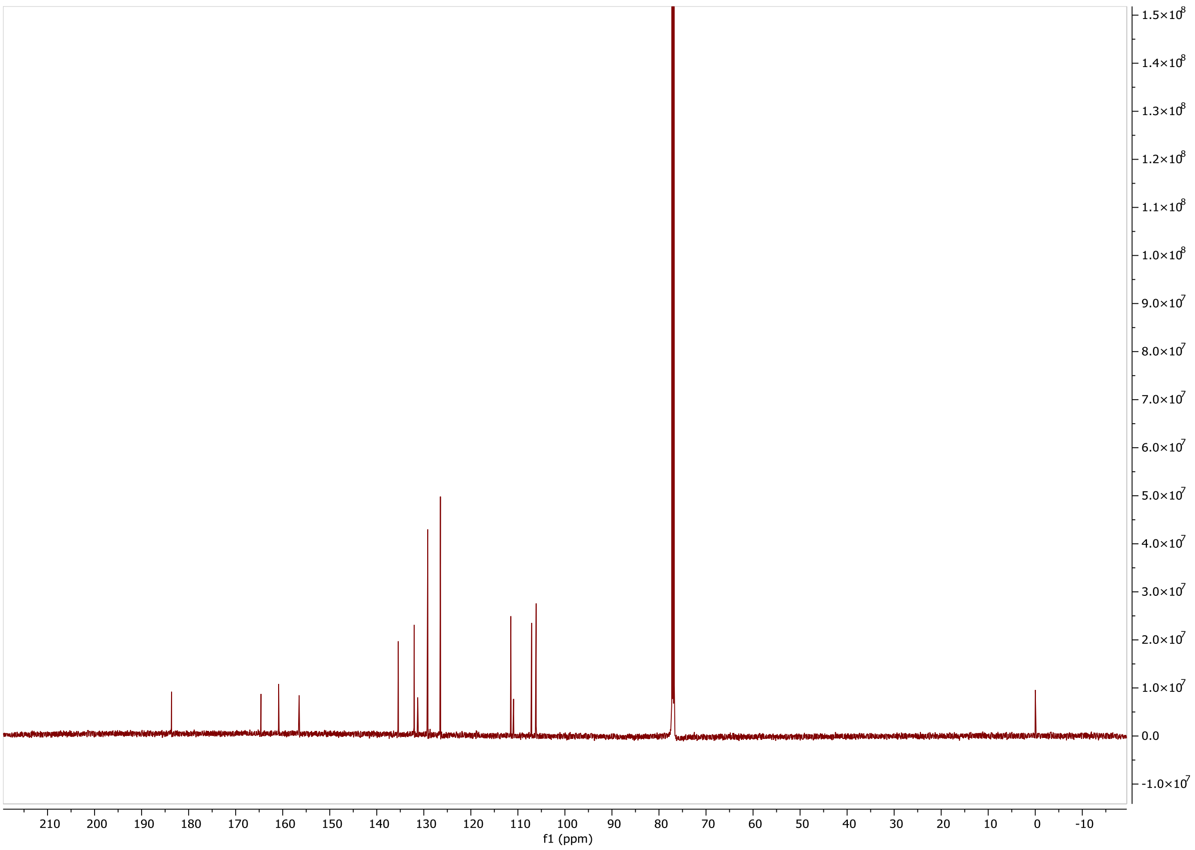

Figure S65. ^{1}H-NMR (600.19 MHz, Acetone-d_6) of compound 23.

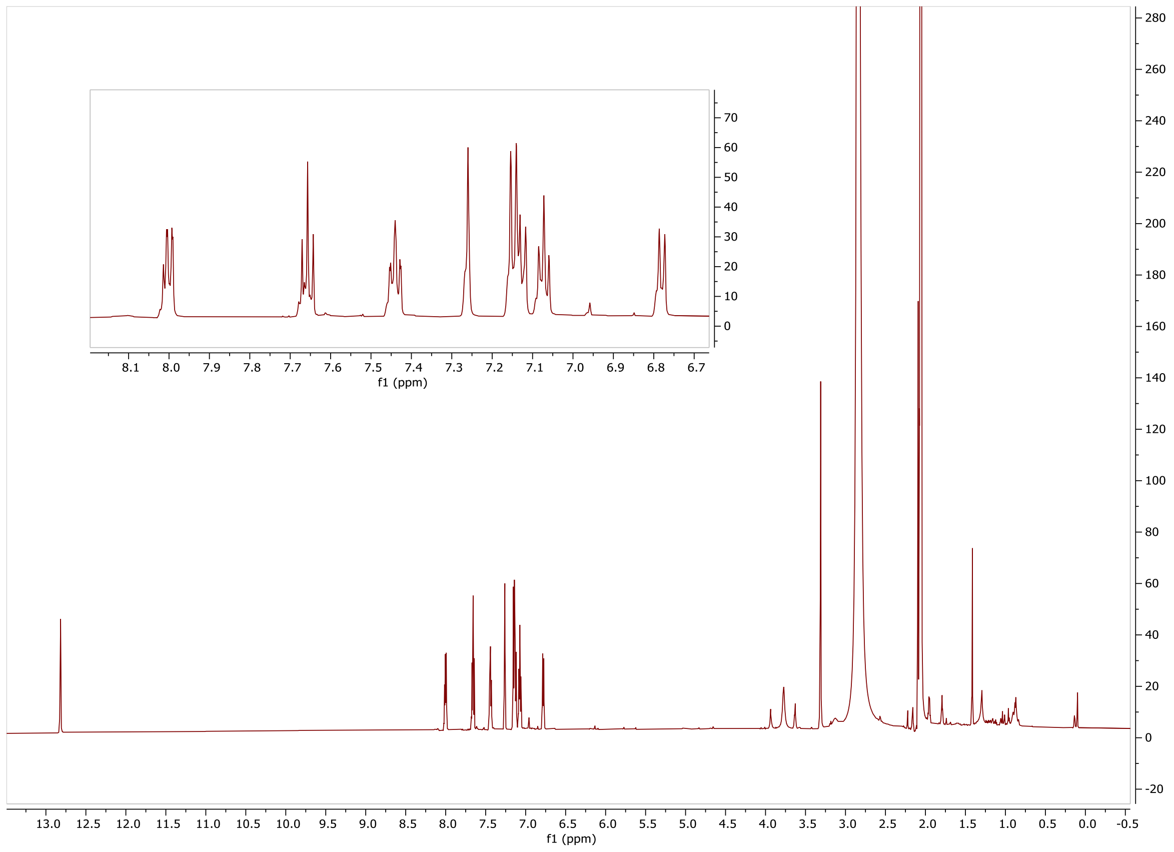

Figure S66. ^{13}C-NMR (150.91 MHz, Acetone-d_6) of compound 23.

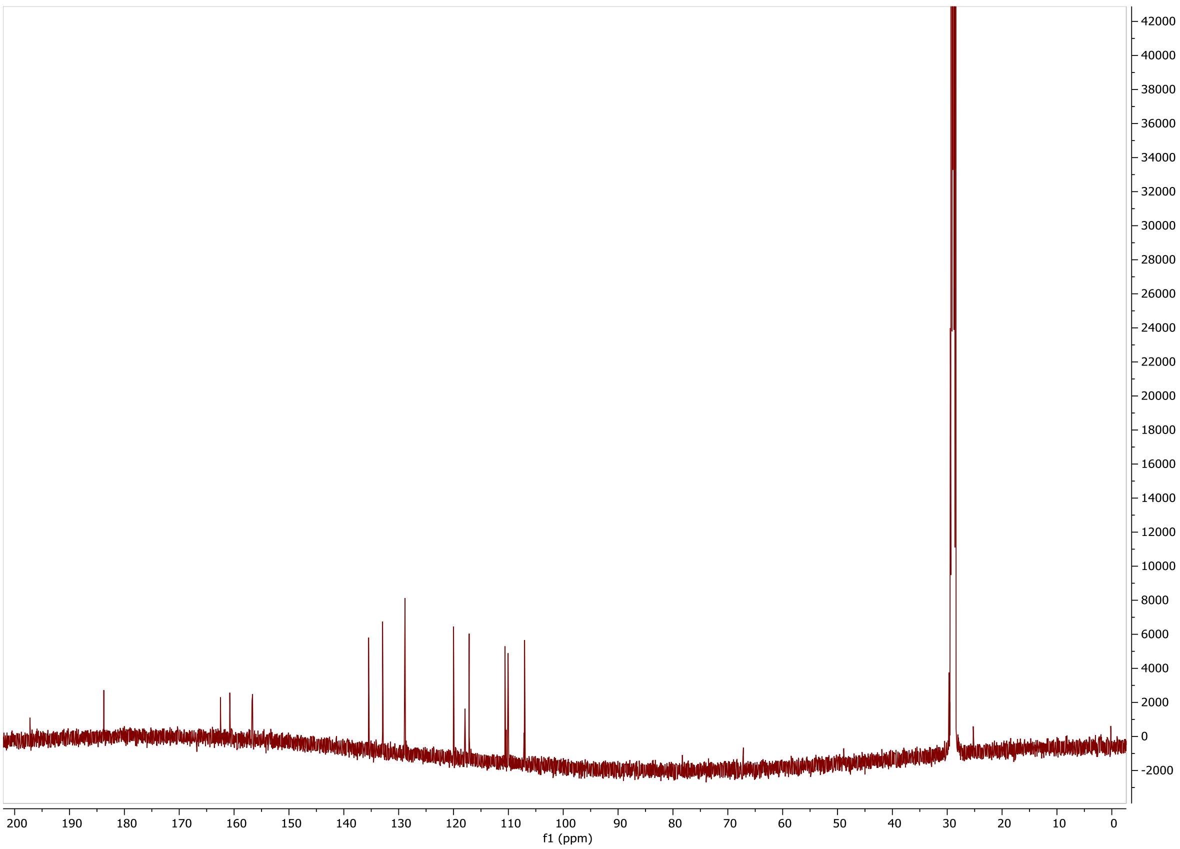

Figure S67. ^{1}**H-NMR (600.19 MHz, MeOH-*d*$_4$) of compound 24.**

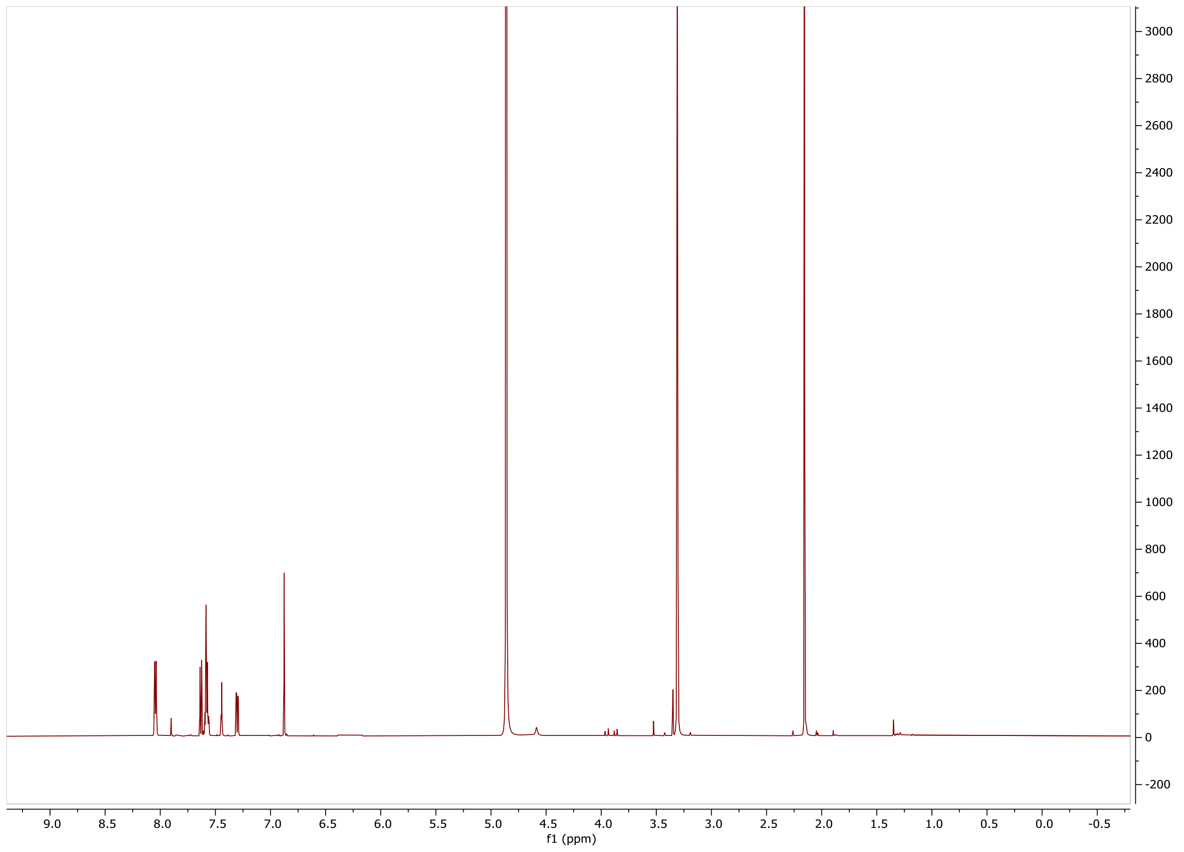

Figure S68. ^{13}**C-NMR (150.91 MHz, MeOH-*d*$_4$) of compound 24.**

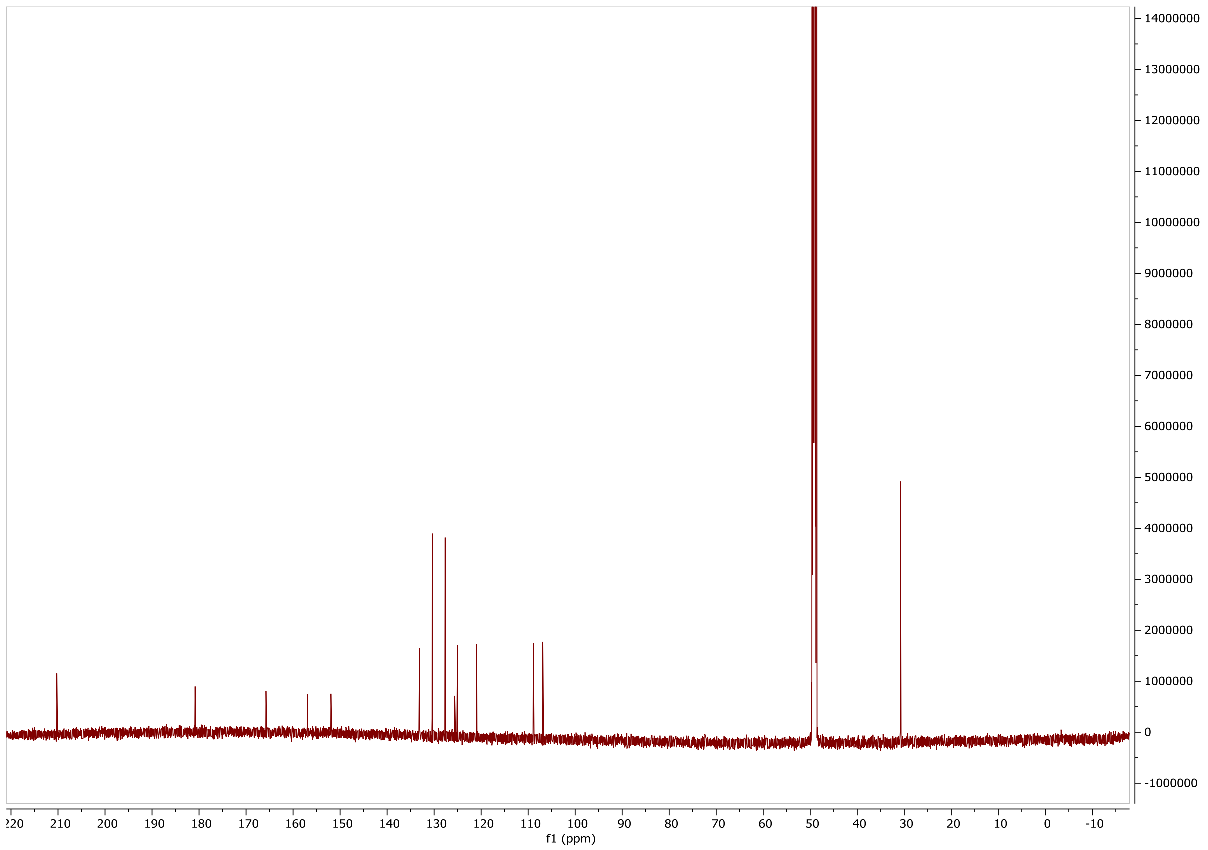

Figure S69. ^{1}H-NMR (600.19 MHz, DMSO-d_6) of compound 25.

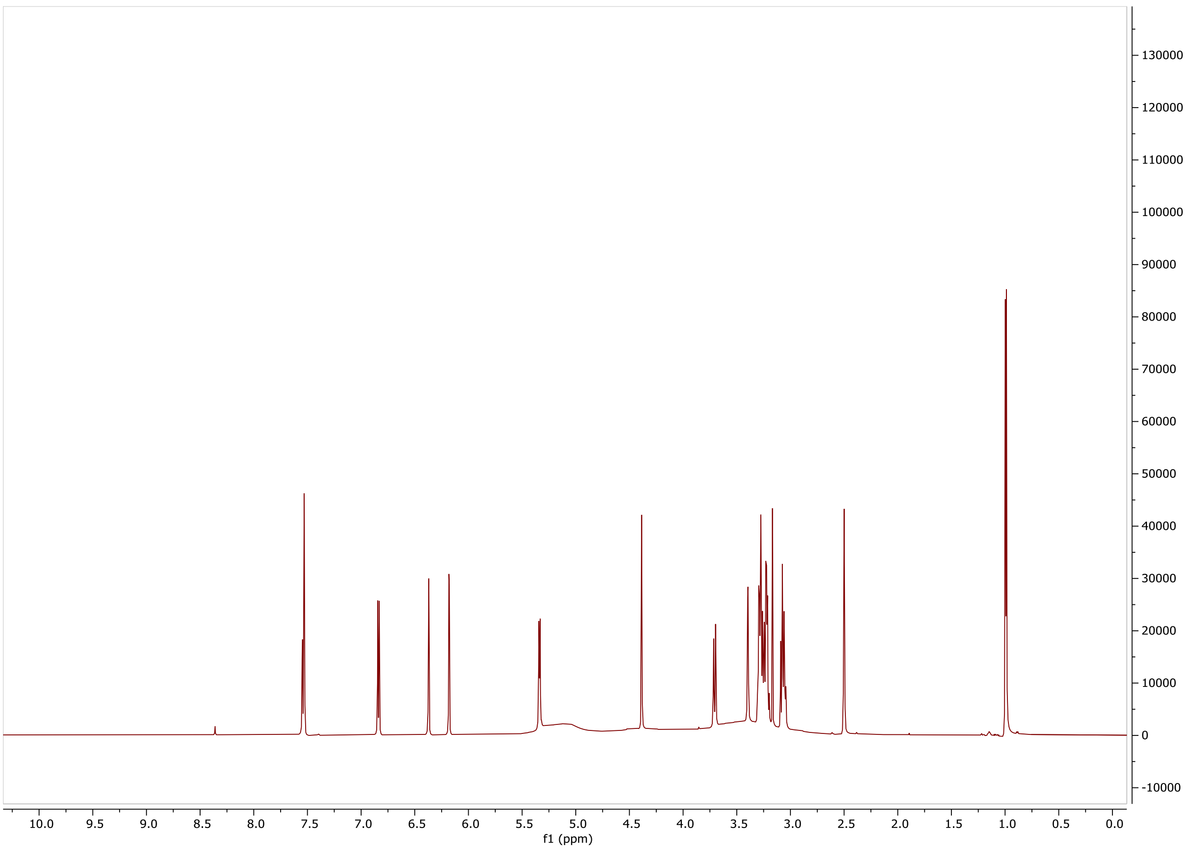

Figure S70. ^{13}C-NMR (150.91 MHz, DMSO-d_6) of compound 25.

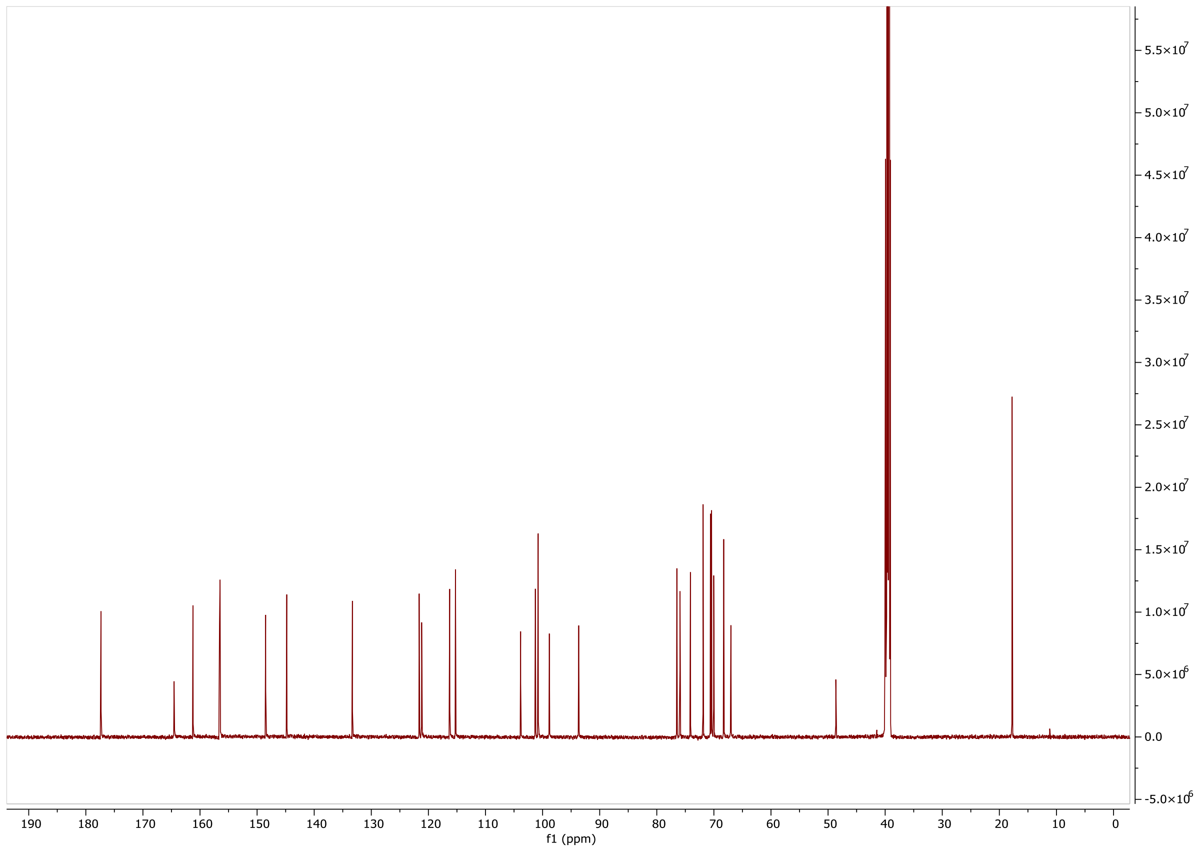

Table S1. Anti-proliferative activity of selected compounds isolated from diethyl ether subfraction of the methanolic extract of *D. diapensifolia* in MTT assay after 24 h shown as % cellular inhibition (MEAN±SEM) of n = 3. ***, **, * denote $p < 0.001$, $p < 0.01$, $p < 0.05$ vs. control.

**Compound**	**50 ~~µg/mL~~µM**	**10 ~~µg/mL~~µM**	**5 ~~µg/mL~~µM**
**1**	0.00±0.00	0.67±0.67	3.33±2.40
**2**	0.13±0.13	0.00±0.00	4.72±2.48
**3**	2.40±2.40	2.27±2.27	0.50±0.50
**4**	1.93±1.93	0.03±0.03	0.70±0.70
**6**	69.43±0.18***	0.00±0.00	0.00±0.00
**8**	0.36±0.36	0.66±0.66	1.40±0.91
**9**	12.63±0.24*	6.23±3.24	3.80±3.10
**10**	40.33±0.88	1.00±0.58	0.00±0.00
**11**	3.90±1.17	6.73±1.34	8.03±1.14

Figure S71. HPLC-DAD chromatogram of *D. diapensifolia n*-butanol subfraction and rutin standard.

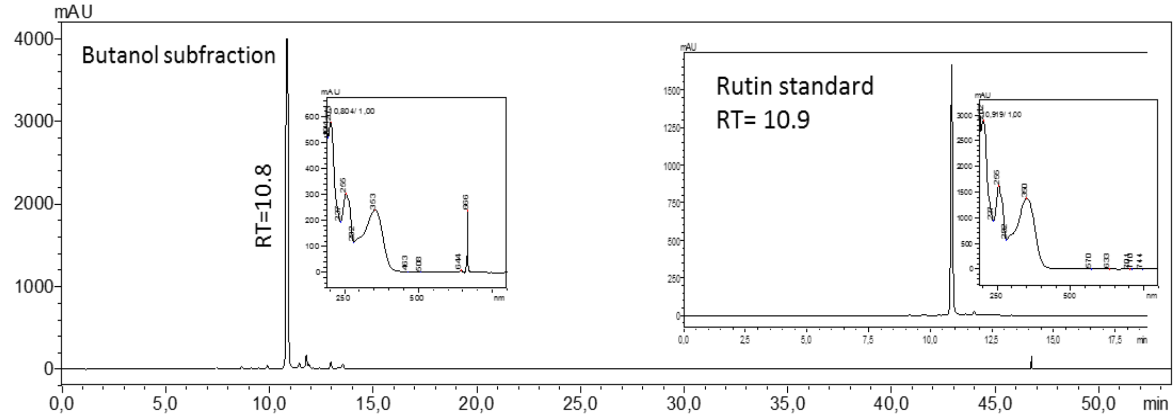

Analysis condition: stationary phase: Phenomenex Aqua C18 5µm, 150 × 4.6 mm; mobile Phase: A = H_2O + 0.02% TFA, B = acetonitrile; gradient: 0 min: B=2%; 20 min: B=50%; 40 min: B=98%, 50 min B=98%; temp.: 35°C; flow: 1 mL/min; butanol subfraction: 1 mg/mL, inj. vol. 10 µL; rutin standard: 2 mg/mL, inj. vol. 10 µL.

Figure S72. Low energy conformers of compound 1. Conformer generation was done on MacroModel 09 (Schrödinger Ltd.), using OPLS-3 as forcefield in gas phase. Conformers occurring in energy window of 5 kcal.mol^{-1} were further optimized in DFT/6-31G(d,p) level in the gas phase using Gaussian 16 v. A3 software [1].

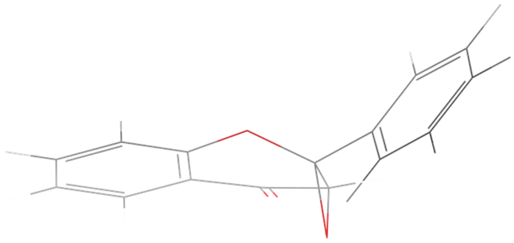

Isomer 1,
C1: 50%

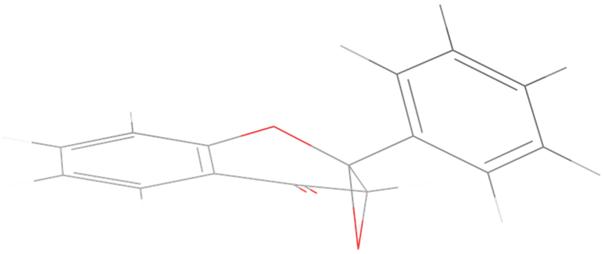

Isomer 1,
C2: 50%

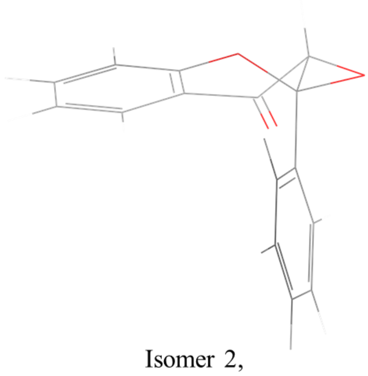

Isomer 2,
C1: 50%

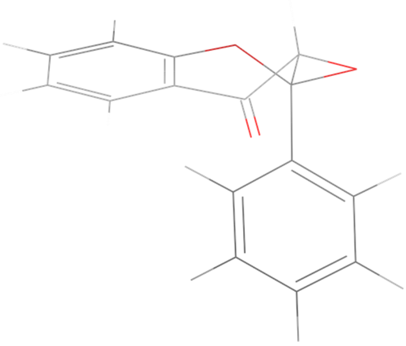

Isomer 2,
C2: 50%

Table S2. Experimental chemical shifts and Boltzmann-averaged shielding tensors of two diastereomers of **1**, used for DP4+ chemical shift calculation.

Atom	*δ*exp (ppm)	Isomer 1	Isomer 2
C	119	53.423	52.492
C	121.6	62.683	54.439
C	159	23.372	13.689
C	126.7	63.755	64.914
C	136.7	44.001	44.863
C	122	58.970	58.636
C	187.5	-9.164	0.477
C	56.2	117.895	114.574
C	84.8	97.934	89.100
C	140.8	47.319	51.255
C	125.9	55.482	52.460
C	129.1	52.892	53.108
C	129.2	51.508	50.935
C	129.1	52.892	53.111
C	125.9	55.481	52.463
H	4.22	28.148	23.798
H	6.74	23.319	24.089
H	8.00	24.147	23.760
H	7.35	23.703	24.298
H	7.00	24.193	27.547
H	7.58	23.723	23.978
H	7.39	23.883	24.077
H	7.35	23.875	24.012
H	7.39	23.883	24.077
H	7.58	23.723	23.978

Figure S73. The result sheet of DP4+ chemical shift probability calculation of two diastereomers of compound 1. Calculation of shift tensors were done using GIAO/mpw1pw91/6-311+(d,p)/CPCM in $CHCl_3$ in Gaussian 16 A.3 [Ref]. DP4+ probability calculation was done using the method originally published by Grimblat et. al [2].

1	Functional	Solvent?		Basis Set		Type of Data	
2	mPW1PW91	PCM		6-311+G(d,p)		Shielding Tensors	
3							
4		Isomer 1	Isomer 2	Isomer 3	Isomer 4	Isomer 5	Isomer 6
5	sDP4+ (H data)	100.00%	0.00%	-	-	-	-
6	sDP4+ (C data)	99.99%	0.01%	-	-	-	-
7	sDP4+ (all data)	100.00%	0.00%	-	-	-	-
8	uDP4+ (H data)	79.58%	20.42%	-	-	-	-
9	uDP4+ (C data)	100.00%	0.00%	-	-	-	-
10	uDP4+ (all data)	100.00%	0.00%	-	-	-	-
11	DP4+ (H data)	100.00%	0.00%	-	-	-	-
12	DP4+ (C data)	100.00%	0.00%	-	-	-	-
13	DP4+ (all data)	100.00%	0.00%	-	-	-	-

References

(1) Frisch, M. J.; Trucks, G. W.; Schlegel, H. B.; Scuseria, G. E.; Robb, M. A.; Cheeseman, J. R.; Scalmani, G.; Barone, V.; Petersson, G. A.; Nakatsuji, H.; Li, X.; Caricato, M.; Marenich, A.; Bloino, J.; Janesko, B. G.; Gomperts, R.; Mennucci, B.; Hratchian, H. P.; Ortiz, J. V.; Izmaylov, A. F.; Sonnenberg, J. L.; Williams-Young, D.; Ding, F.; Lipparini, F.; Egidi, F.; Goings, J.; Peng, B.; Petrone, A.; Henderson, T.; Ranasinghe, D.; Zakrzewski, V. G.; Gao, J.; Rega, N.; Zheng, G.; Liang, W.; Hada, M.; Ehara, M.; Toyota, K.; Fukuda, R.; Hasegawa, J.; Ishida, M.; Nakajima, T.; Honda, Y.; Kitao, O.; Nakai, H.; Vreven, T.; Throssell, K.; Montgomery, J. A. J.; Peralta, J. E.; Ogliaro, F.; Bearpark, M.; Heyd, J. J.; Brothers, E.; Kudin, K. N.; Staroverov, V. N.; Keith, T.; Kobayashi, R.; Normand, J.; Raghavachari, K.; Rendell, A.; Burant, J. C.; Iyengar, S. S.; Tomasi, J.; Cossi, M.; Millam, J. M.; Klene, M.; Adamo, C.; Cammi, R.; Ochterski, J. W.; Martin, R. L.; Morokuma, K.; Farkas, O.; Foresman, J. B.; Fox, D. J. Gaussian 16, Revision A.03, Gaussian, Inc.: Wallingford, CT, USA 2016.

(2) Grimblat, N.; Zanardi, M. M.; Sarotti, A. M. Beyond DP4: An Improved Probability for the Stereochemical Assignment of Isomeric Compounds Using Quantum Chemical Calculations of NMR Shifts. *J. Org. Chem.* **2015**, *80* (24), 12526–12534.https://doi.org/10.1021/acs.joc.5b02396.
